# Water-promoted dehydrative coupling of 2-aminopyridines in heptane *via* a borrowing hydrogen strategy†

**DOI:** 10.1039/d1ra04118e

**Published:** 2021-07-01

**Authors:** Taku Nakayama, Hidemasa Hikawa, Shoko Kikkawa, Isao Azumaya

**Affiliations:** Faculty of Pharmaceutical Sciences, Toho University 2-2-1 Miyama, Funabashi Chiba 274-8510 Japan hidemasa.hikawa@phar.toho-u.ac.jp isao.azumaya@phar.toho-u.ac.jp

## Abstract

A synthetic method for dehydrative *N*-benzylation promoted by water molecules in heptane using a π-benzylpalladium system has been developed. The presence of water significantly accelerates carbon–nitrogen bond formation, which is accomplished in an atom-economical process to afford the corresponding *N*-monobenzylated products. A crossover experiment afforded H/D scrambled products, which is consistent with a borrowing hydrogen mechanism. Kinetic isotope effect measurements revealed that benzylic carbon–hydrogen bond cleavage was the rate-determining step.

## Abstract 2

We describe a novel strategy for the water-promoted dehydrative coupling reaction in heptane, which offers a sustainable direct amination of alcohols.

## Graphical abstract

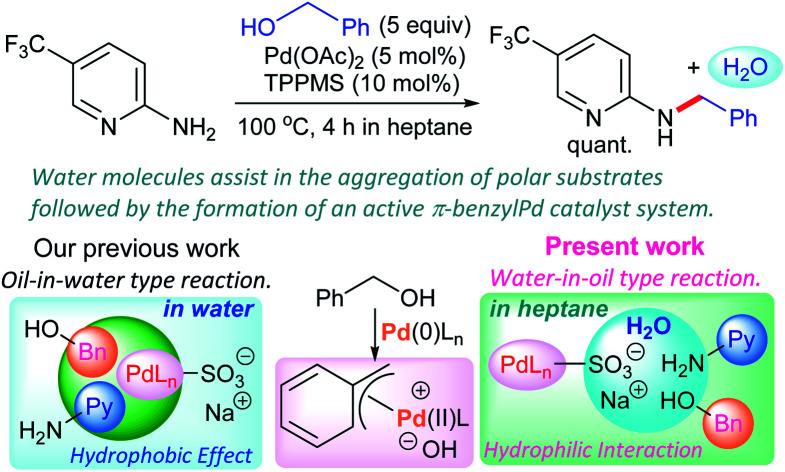

## Introduction

The borrowing hydrogen methodology has emerged as a promising greener synthetic strategy for straightforward carbon–nitrogen bond formation utilizing readily available and low-toxicity benzyl alcohols instead of benzyl halides as coupling partners.^1^ Various catalyst systems using iridium(iii),^2^ ruthenium(ii),^3^ or other metals^4^ have been developed. Although this strategy affords an attractive atom-economical pathway and is an appealing synthetic shortcut for constructing valuable products, the reactions generally require high temperatures, organic solvents and strong bases. Thus, an efficient system possessing greater catalytic activity that can be performed at lower temperatures in greener solvents without any additives is highly desirable for sustainable carbon–nitrogen bond forming reactions.^5^ Recently, our group has been exploring a greener borrowing hydrogen methodology involving π-benzylpalladium(ii) complexes^6^ in water.^7^

Recent studies disclosed that hydrophilic interactions play an important role in controlling self-assembly in biological processes.^8^ In 2008, McLain *et al.* showed that charge-based interactions play an important role in dipeptide association in aqueous solution.^8*b*^ In non-polar organic solvents, reverse micelles (RMs), with the polar groups concentrated in the interior of the aggregate, are formed *via* the self-assembly of surfactant molecules.^9^ Notably, the presence of water molecules is the driving force for reverse micelle formation in nanosized water pools.^9*b*^ In 2014, Zhao *et al.* prepared gold cluster catalysts within interfacially cross-linked reverse micelles *via* extraction of HAuCl_4_ in the hydrophilic core for intramolecular alkyne carboxylation.^9*c*^

**Scheme 1:**
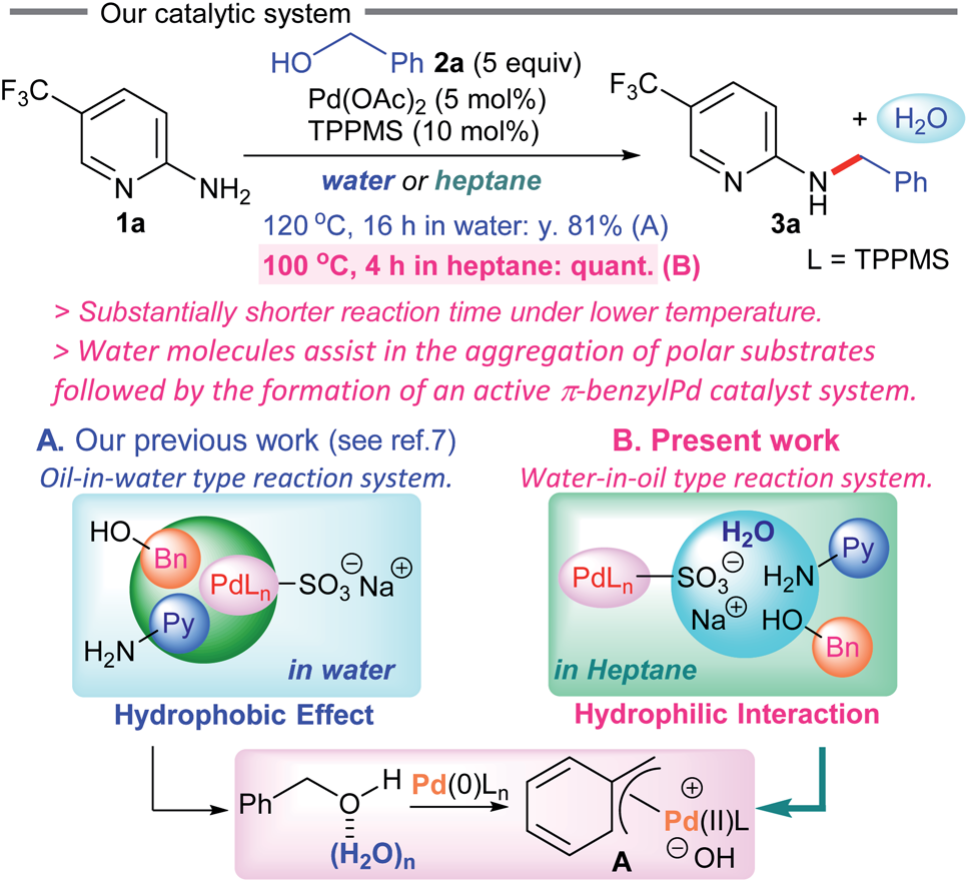
Pd-catalyzed dehydrative coupling between aminopyridine 1a and alcohol 2a.

Inspired by these reports, we became interested in designing a new catalytic system *via* water-assisted self-assembly of polar substrates with a palladium catalyst in non-polar solvents (Scheme 1). We herein present the dehydrative coupling of 2-aminopyridines with benzylic alcohols in heptane using a π-benzylpalladium system, furnishing the corresponding *N*-benzyl-2-aminopyridines.^10^ As the reaction begins, *in situ* generated water accelerates the aggregation of polar substrates with a Pd(0)/TPPMS catalyst in heptane, forming an active π-benzylpalladium catalyst system. The oxidative addition of alcohols to palladium(0) complexes is generally difficult due to the poor performance of the hydroxyl moiety as a leaving group. In the present work, the first example of direct conversion of non-activated benzyl alcohols to the π-benzylPd(ii) system has been developed in organic solvents, and applied to the dehydrative cross-coupling reaction. This water-in-oil reaction system is substantially different from our previous method (an oil-in-water type reaction system) concerning the self-assembly between palladium catalysts and polar substrates (Scheme 1A *vs.* B), and shows a significant advancement over our previous synthetic protocol for more sustainable chemistry.

Achieving the direct introduction of various functionalities to aminopyridines, which are found in a wide variety of pharmaceuticals, is of enormous interest.^11^ Notably, our π-benzylPd(ii) system can be applied to a variety of 2-aminopyridine substrates and benzylic alcohols under mild conditions.

## Results and discussion

### 1. Optimization of *N*-benzylation

We initially examined the Pd-catalyzed reaction between 5-(trifluoromethyl)pyridin-2-amine (1a) and benzyl alcohol (2a) in heptane (Table 1). The reaction was completed in 4 h at 100 °C under air, furnishing the desired product 3a (entry 1). When using 3 equiv. of alcohol 2, the reaction did not proceed after 4 h, but was completed in 16 h (entry 2). Screening of other palladium catalysts found that palladium(ii) trifluoroacetate resulted in an almost quantitative yield, whereas palladium(ii) dichloride was not applicable (entries 3 and 4). The reaction using tris(dibenzylideneacetone)dipalladium(0) at 120 °C for 16 h gave 3a in 77% yield (entry 5). Although the reaction proceeded to completion using octane instead of heptane (entry 6), the use of other low-polarity organic solvents resulted in lower yields (entries 7–11). Furthermore, little reaction occurred under neat conditions (entry 12). Other water-soluble phosphine ligands such as P(C_6_H_4_SO_3_Na)_3_L2 (TPPTS) and (diphenylphosphino)benzoic acids L3–4 were less effective than TPPMS (entries 13–15), and a lower yield was also obtained without a phosphine ligand (entry 16). Since the reaction was completed in 4 h under an Ar atmosphere, oxygen was not essential to the borrowing hydrogen reaction (entry 17).

**Table 1:** Reaction optimization^a^

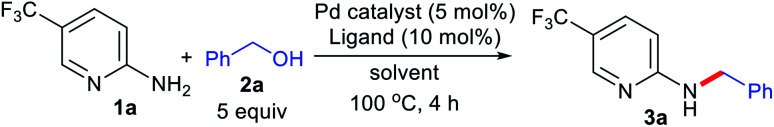				
Entry	Catalyst	Ligand	Solvent	NMR yield (%)
1	Pd(OAc)_2_	L1	Heptane	Quant
2	Pd(OAc)_2_	L1	Heptane	0^b^ (quant)^c^
3	Pd(TFA)_2_	L1	Heptane	97
4	PdCl_2_	L1	Heptane	0
5^d^	Pd_2_(dba)_3_·CHCl_3_^e^	L1	Heptane	77
6	Pd(OAc)_2_	L1	Octane	Quant
7	Pd(OAc)_2_	L1	Hexane	2
8	Pd(OAc)_2_	L1	MCH^f^	38
9	Pd(OAc)_2_	L1	Toluene	4
10	Pd(OAc)_2_	L1	(CHCl_2_)_2_	0
11	Pd(OAc)_2_	L1	1,4-Dioxane	20
12	Pd(OAc)_2_	L1	None	8
13	Pd(OAc)_2_	L2	Heptane	5
14	Pd(OAc)_2_	L3	Heptane	0
15	Pd(OAc)_2_	L4	Heptane	6
16	Pd(OAc)_2_	None	Heptane	30
17^g^	Pd(OAc)_2_	L1	Heptane	99
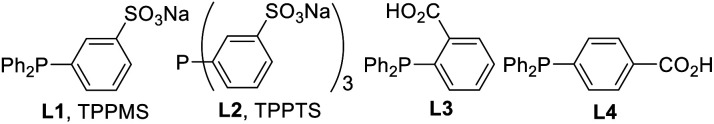				

a Optimizations were performed with 1 mmol of 1a and 5 mmol of 2a in the presence of 5 mol% Pd catalyst and 10 mol% TPPMS (10 mol%) in solvent (4 mL) at 100 °C for 4 h under air.

b 3 equiv. of alcohol 2a.

c For 16 h.

d Conducted at 120 °C for 16 h.

e 2.5 mol%.

f Methylcyclohexane.

g Under Ar.

### 2. Reaction scope

**Scheme 2:**
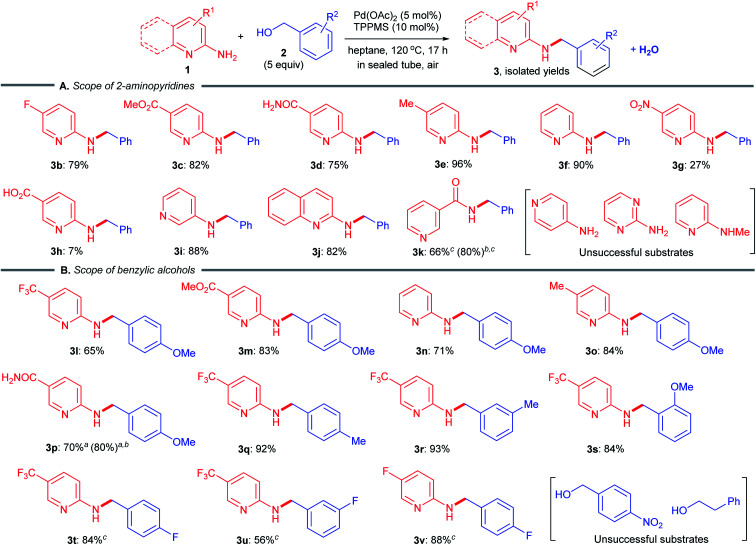
Substrate scope of *N*-benzylation. Yield of isolated products. ^*a*^3 equiv. of alcohol. ^*b*^NMR yield. ^*c*^10 mol% Pd(OAc)_2_, 20 mol% TPPMS, 150 °C, 24 h in octane.

Next, we explored the scope of 2-aminopyridines 1 and benzylic alcohols 2 capable of undergoing a borrowing hydrogen reaction (Scheme 2). A variety of 2-aminopyridines 1 were well tolerated, giving the desired products 3b–e in 75–96% yields (Scheme 2A). The reaction of simple 2-aminopyridine, bearing no substituent at the 5-position, also proceeded smoothly (3f, 90%). Advantageously, a substrate containing a sensitive reducible nitro group could be utilized, furnishing the corresponding desired product 3g, albeit in low yield. The reaction of 6-aminonicotinic acid gave an extremely poor result (only 7% isolated yield of *N*-benzylated product 3h). To our delight, the utilization of benzyl alcohol (2a) directly as a coupling partner could be applied to 3-aminopyridine, 2-aminoquinoline and nicotinamide (niacinamide) as amine substrates, furnishing the corresponding pharmaceutically active *N*-benzylated motifs in moderate to excellent yields (3i 88%; 3j, 82%; 3k, 66%).^12^ Unfortunately, no reactions occurred when using 4-aminopyridine, 2-aminopyrimidine and 2-(methylamino)pyridine since the amino groups had poor nucleophilicity. Benzylic alcohols with electron-donating groups were effective coupling partners (3j–s, 65–93%) (Scheme 2B). A sterically hindered 2-methoxybenzyl alcohol (2s) was well tolerated to obtain the *N*-benzylated product 3s in 84% yield. Furthermore, the reactions of electron-deficient fluorobenzyl alcohols afforded the desired products 3t–v in 56–88% yields, although in octane, higher temperatures were required to achieve better yields. In contrast, no reaction proceeded when using 4-nitrobenzyl alcohol and 2-phenylethyl alcohol, likely because the corresponding π-benzyl Pd(ii) cation species was not generated.

### 3. Effect of water

**Fig. 1:**
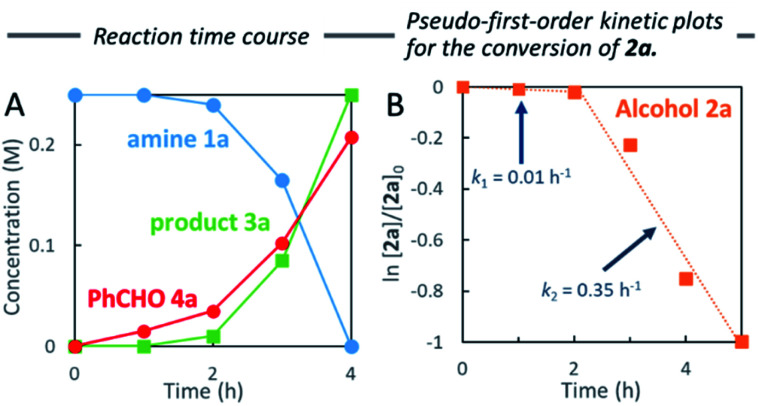
Monitoring the reaction.

To measure the full reaction profile, dehydrative coupling between 2-aminopyridine 1a and alcohol 2a at 100 °C in heptane was monitored by ^1^H NMR spectroscopy. The formation of *N*-benzylated product 3a along with benzaldehyde (4a) was observed (Fig. 1A). No imine intermediate was detected, suggesting its rapid hydrogenation to the corresponding desired product 3a. The consumption of alcohol 2a followed pseudo-first-order kinetics including the presence of two mechanistic periods (0–2 h: *k*_1_ = 0.01 h^−1^; 2–4 h: *k*_2_ = 0.35 h^−1^) (Fig. 1B). Based on these results, we hypothesized that *in situ* generated water molecules might promote the dehydrogenation of 2a. Therefore, we investigated the detailed reaction mechanism, especially the role of water molecules in heptane.

**Scheme 3:**
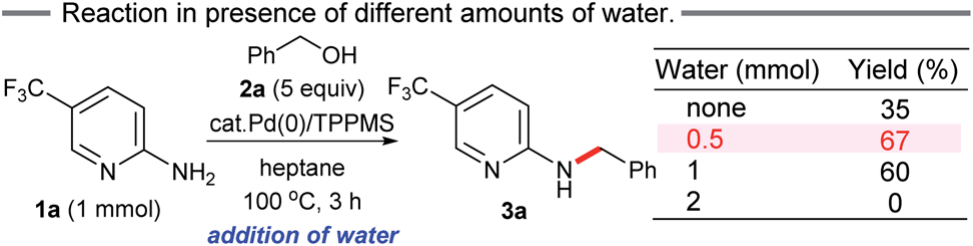
*N*-Benzylation in the presence of different amounts of water.

Thus, we conducted the reaction while adding different amounts of water (0.5, 1 and 2 mmol) (Scheme 3). As expected, the initial reaction rate in heptane dramatically increased upon addition of 0.5 mmol water compared to no water. The addition of 1 mmol water resulted in almost the same outcome (60%). In contrast, no benzylation was observed in the presence of excess water (2 mmol). When changing the additional water ratio, the reaction was accelerated in the following order: 0.5 mmol ≈ 1 mmol > no addition of water (*in situ* generated 0.35 mmol of water molecules after 3 h) ≫ 2 mmol, suggesting that traces of water present *in situ* (≤1 mmol) promoted aggregation between the polar substrates and the Pd catalyst in heptane, forming the active π-benzylpalladium(ii) catalyst system. Since the formation of reverse micelles *via* aggregation of polar substrates did not occur by adding excess water, the water-in-oil type reaction employing the π-benzylpalladium species did not take place. Indeed, the addition of water (1 mmol) was not effective without stirring or addition of *n*-Bu_4_NBr.

**Fig. 2:**
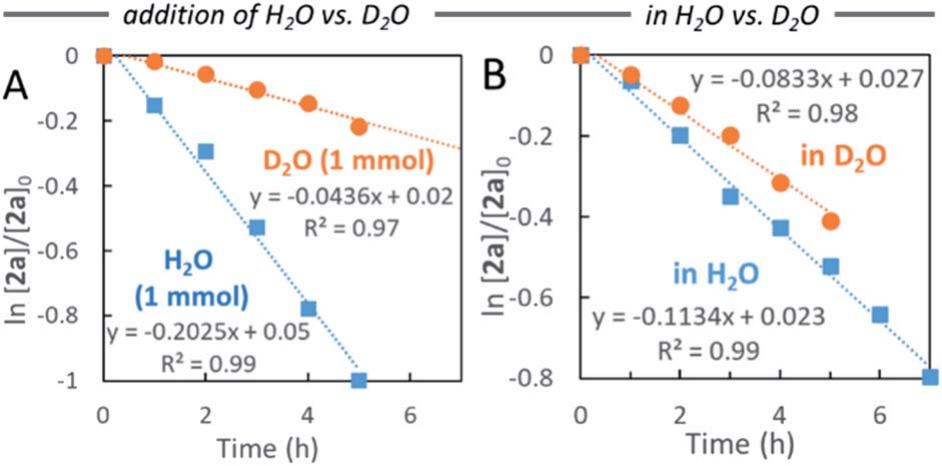
Comparison of pseudo-first-order kinetic plots for reaction of 2a in heptane (A) with addition of H_2_O *vs.* D_2_O, and (B) in H_2_O *vs.* D_2_O.

Water-promoted dehydrogenation of 2a in heptane exhibited pseudo-first-order plots with a higher primary kinetic solvent isotope effect (KSIE = 4.6). This value was much larger than the KSIE value of 1.4 measured in water reactions (Fig. 2A and B),^7*b*^ suggesting that hydrogen bonds play a significant role in the dehydrative coupling reaction in heptane.

### 4. Deuterium labeling experiment

**Scheme 4:**
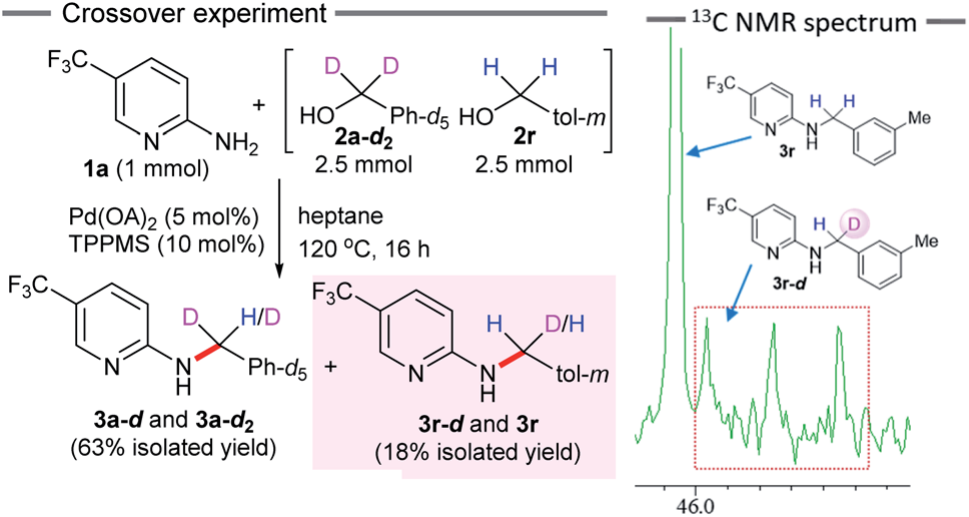
Deuterium labeling experiment.

We performed a crossover experiment by subjecting an equimolar amount of deuterated benzyl alcohol 2a-*d*_2_ (5 mmol) and 3-methylbenzyl alcohol 2r (5 mmol) to the standard reaction conditions (Scheme 4). If the nucleophilic substitution of alcohol 2r (dehydrative Tsuji–Trost type reaction) proceeds, deuterium labeled product 3r-*d* would not be obtained. In contrast, a borrowing hydrogen reaction would afford the H/D scrambled *N*-benzylated product 3r-*d*. As expected, a mixture of crossover product 3r-*d* and non-crossover product 3r was obtained in 18% yield. The results obtained from a ^13^C NMR analysis showed that the carbon of the methylene group exhibited a 1 : 1 : 1 triplet for 3r-*d*. Furthermore, the ratio of the integrated values in the ^1^H NMR spectrum showed 18% incorporation of deuterium at the benzylic position of 3r-*d* (see ESI†). This experimental evidence clearly indicates a borrowing hydrogen reaction and rules out an alternative Pd-catalyzed S_N_2-type substitution reaction.

### 5. Mechanistic studies

**Scheme 5:**
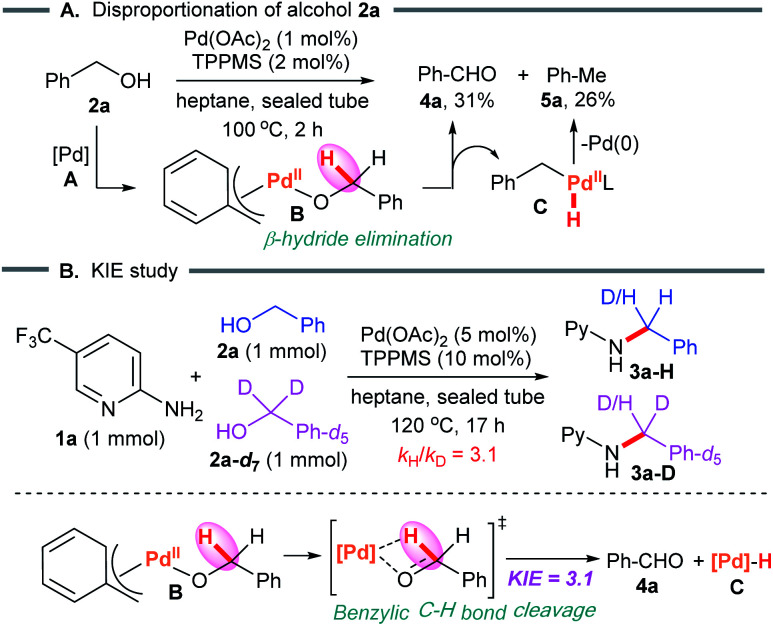
Mechanistic studies.

First, we examined the Pd-catalyzed disproportionation of alcohol 2a (Scheme 5A). When the alcohol 2a was converted to aldehyde 4a, the generation of toluene (5a) was clearly observed by ^1^H NMR spectroscopy. This result suggested that β-hydride elimination of benzylPd(ii)-alkoxide complex B led to the aldehyde 4a, which then reacted with benzylPd(ii) hydride species C*via* reductive elimination to form toluene 5a with regenerated Pd(0)L_*n*_.

We next studied the kinetic isotope effect in an intermolecular competition experiment using benzyl alcohol and its deuterium-labeled analog. As expected, a kinetic isotope effect (KIE) of 3.1 was observed for the reaction with substrate 1a (Scheme 5B). A KIE of this magnitude is in agreement with the benzylic C–H bond cleavage featured in the turnover limiting step.

### 6. Mechanistic considerations

**Scheme 6:**
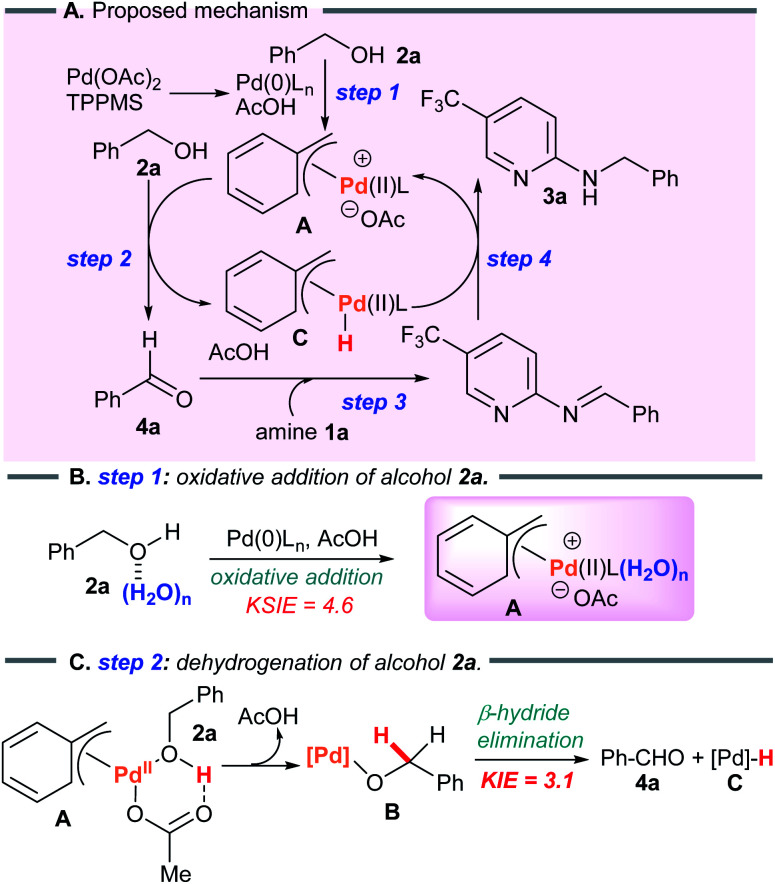
Proposed catalytic cycle.

Considering the results above, a proposed catalytic cycle for the borrowing hydrogen reaction in heptane is illustrated in Scheme 6.

#### Step 1

Initially, oxidative addition of alcohol 2a to the Pd(0) species^13^ generates the π-benzylPd(ii) intermediate A.^14^ In this step, polar substrates with water molecules activate the hydroxy group of 2a through a hydrogen bonding network to generate complex A.^15,16^ This mechanism is consistent with the observed KSIE (*k*H_2_O/*k*D_2_O = 4.6).

#### Step 2

Next, β-hydride elimination affords the key intermediate aldehyde 4a.^17^ Since the alcohol–Pd(ii) complex is deprotonated by the acetoxy anion,^17*b*,18^ strong bases are not required. The C–H bond cleavage of alcohol 2a*via* β-hydride elimination is the rate-determining step (KIE value of 3.1 in Scheme 5B). Notably, water molecules promote the aggregation of polar substrates with palladium catalysts in heptane, furnishing the active π-benzylpalladium system smoothly in the rate-determining step. Furthermore, benzyl alcohol (2a) disproportionates to benzaldehyde with toluene (Scheme 5A), which provides support for the proposed π-benzylpalladium catalyst mechanism.

#### Steps 3 and 4

Finally, reductive amination between aldehyde 4a and the amine substrate catalyzed by Pd(ii) hydride C proceeds to generate the desired product 3a and regenerates the benzylPd(ii) A.

**Scheme 7:**
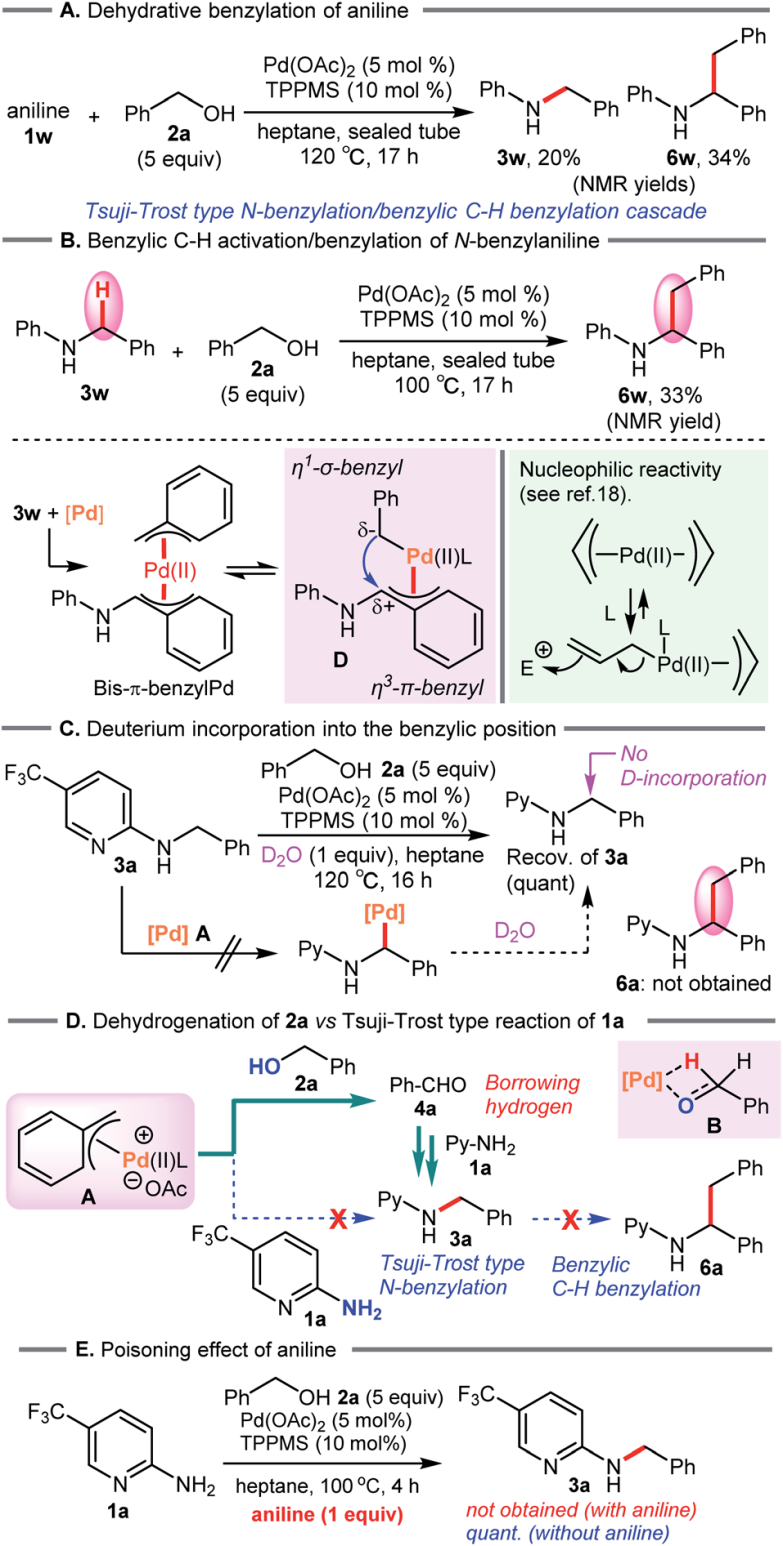
(A) and (B) Pd-catalyzed benzylation of aniline. (C) Deuterium labeling study. (D) Proposed reaction pathway. (E) *N*-Benzylation of 2-aminopyridine 1a in the presence of aniline.

To verify the formation of the π-benzylpalladium catalyst system, we examined the Tsuji–Trost type reaction of aniline (1w) in heptane (Scheme 7A). An electron-sufficient amine substrate should react with the π-benzylPd(ii) species by nucleophilic substitution. As expected, the reactivity of 1w was significantly different from that of 2-aminopyridine 1a, furnishing not only *N*-benzylaniline (3w) but also dibenzylated product 6w (3w, 20%; 6w, 34%). Furthermore, when using 3w as a starting material, the corresponding dibenzylated product 6w was formed *via* the condensation of an alcohol with a benzylic C–H bond (Scheme 7B). These results provide convincing evidence supporting the formation of the π-benzylPd(ii) species. This catalyst system enables the dehydrative *N*-benzylation C–H bond activation cascade reaction of electron-rich analog 3w. The η^1^-σ-benzyl nucleophile attacks an electrophilic η^3^-π-benzyl moiety in the bis-benzylPd(ii) complex D, generating a new benzylic C–C bond. This proposed mechanism is consistent with the nucleophilic reactivity of bis-π-allylpalladium complexes reported by Yamamoto *et al.*^19^ Indeed, Pd-catalyzed benzylic C–H benzylation of electron-sufficient *N*-benzylpyridine substrate 3a did not occur (Scheme 7C). Furthermore, the treatment of 3a with D_2_O (1 equiv.) as the D-atom source in heptane showed no deuterium incorporation at the benzylic position of 3a (The reaction in D_2_O as a solvent also gave the same result, see ESI†). These results clearly demonstrated that Pd-catalyzed benzylic C–H bond cleavage of 3a did not occur, and therefore rule out D/H exchange between *N*-benzylated product 3a-*d*_2_ and 3r in our crossover experiment (see Scheme 4).

On the basis of these results, dehydrogenation of benzyl alcohol (2a) by π-benzylPd(ii) complex A proceeds faster than nucleophilic substitution of species A, and leaves the weak 2-aminopyridine nucleophile 1a intact (Scheme 7D). Subsequently, reductive amination of 1a with the resulting aldehyde 4a leads to 3a*via* the borrowing hydrogen pathway. This is consistent with the result of the crossover experiment (see Scheme 4). When adding aniline (1w), *N*-benzylated product 3a was not obtained due to the poisoning effect of aniline (Scheme 7E).

**Scheme 8:**
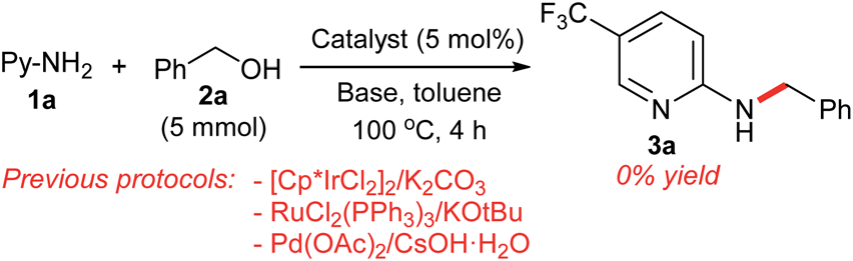
Comparison of the catalytic activity.

Having established a successful dehydrative *N*-benzylation strategy, we compared the catalytic activity of our π-benzylpalladium catalyst system with other efficient systems (Scheme 8). While the Pd(0)/TPPMS-catalyzed reaction in heptane proceeded to completion within 4 h (see Table 1), the previous borrowing hydrogen protocols^10*c*,20^ resulted in no reaction. Furthermore, the Pd-catalyzed reaction in water (our previous work)^7*b*^ was not effective (only 40% yield), clearly demonstrating the superiority of the present catalytic strategy using heptane.

To highlight the synthetic utility, we performed the *N*-benzylation on a gram scale (see ESI†). *N*-Benzylated product 3a (1.56 g) was facilely isolated in 88% yield from pyridine substrate 1a with alcohol 2a. The developed simple operations also avoided the use of column chromatography for a gram scale reaction.

## Conclusions

In summary, we report an efficient palladium-catalyzed dehydrative *N*-benzylation in heptane *via* the borrowing hydrogen methodology. The strategy provides an efficient method for the facile synthesis of benzylaminopyridines, which are found in a wide variety of pharmaceuticals. Notably, the use of a Pd(0)/TPPMS catalyst in a non-polar heptane solvent with a trace amount of water is critical for our catalytic system. Water molecules assist in the aggregation of polar substrates followed by the formation of an active π-benzylpalladium catalyst system in heptane, which significantly boosts the borrowing hydrogen reaction. We expect that this study will aid in the design of new catalytic systems using non-polar solvents, and will serve as an entry point for reaction discovery.

## Experimental

### General procedure

To a sealed tube were added 2-aminopyridines 1 (1 mmol), Pd catalyst (0.05 mmol), phosphine ligand (0.1 mmol), alcohols 2 (5 mmol) and heptane (4 mL). The resulting reaction mixture was heated at 120 °C for 17 h under air. After completion of the reaction, the mixture was cooled to room temperature. The reaction mixture was diluted with water, and then extracted with EtOAc. The combined organic phases were washed with brine, dried over MgSO_4_, and filtered and concentrated to dryness under reduced pressure. The resulting residue was purified by flash column chromatography on silica gel (eluent: *n*-hexane/EtOAc) to obtain desired products 3.

## Conflicts of interest

There are no conflicts to declare.

## Supplementary Material

RA-011-D1RA04118E-s001

## References

[cit1] Irrgang T., Kempe R. (2019). Chem. Rev..

[cit2] Onoda M., Fujita K.-i. (2020). Org. Lett..

[cit3] Biswas N., Sharma R., Srimani D. (2020). Adv. Synth. Catal..

[cit4] Hofmann N., Homberg L., Hultzsch K. C. (2020). Org. Lett..

[cit5] Risi C., Calamante M., Cini E., Faltoni V., Petricci E., Rosati F., Taddei M. (2020). Green Chem..

[cit6] Le Bras J., Muzart J. (2016). Eur. J. Org. Chem..

[cit7] Hikawa H., Tan R., Tazawa A., Kikkawa S., Azumaya I. (2020). Eur. J. Org. Chem..

[cit8] Rhys N. H., Soper A. K. (2015). J. Phys. Chem. B.

[cit9] Lee L.-C., Xing X., Zhao Y. (2017). ACS Appl. Mater. Interfaces.

[cit10] Shan S. P., Seayad A. M. (2013). ACS Catal..

[cit11] Hiranaka S., Tega Y., Higuchi K., Kurosawa T., Deguchi Y., Arata M., Ito A., Yoshida M., Nagaoka Y., Sumiyoshi T. (2018). ACS Med. Chem. Lett..

[cit12] Taylor S. J., Soleymanzadeh F., Eldrup A. B., Farrow N. A., Muegge I., Kukulka A., Kabcenell A. K., De Lombaert S. (2009). Bioorg. Med. Chem. Lett..

[cit13] Amatore C., El Kaim L., Grimaud L., Jutand A., Meignie A., Romanov G. (2014). Eur. J. Org. Chem..

[cit14] Le Bras J., Muzart J. (2017). Eur. J. Org. Chem..

[cit15] Ma X., Yu J., Han C., Zhou Q., Ren M., Li L., Tang L. (2019). Adv. Synth. Catal..

[cit16] Lu L.-H., Wang Z., Xia W., Cheng P., Zhang B., Cao Z., He W.-M. (2019). Chin. Chem. Lett..

[cit17] Privalov T., Linde C., Zetterberg K., Moberg C. (2005). Organometallics.

[cit18] Wang L.-M., Kobayashi K., Arisawa M., Saito S., Naka H. (2019). Org. Lett..

[cit19] Nakamura H., Iwama H., Yamamoto Y. (1996). Chem. Commun..

[cit20] Fujita K., Li Z., Ozeki N., Yamaguchi R. (2003). Tetrahedron Lett..

