# Supplementary material for: Water-promoted dehydrative coupling of 2-aminopyridines in heptane via a borrowing hydrogen strategy
Source: RSC Adv. 2021 Jul 1;11(37):23144–50. doi: 10.1039/d1ra04118e (PMC9034306; doi:10.1039/d1ra04118e)

Electronic Supplementary Material (ESI) for RSC Advances.
This journal is © The Royal Society of Chemistry 2021

Supporting Information

Water-promoted dehydrative N-benzylation of 2-aminopyridines in heptane via borrowing hydrogen methodology

Taku Nakayama, Hidemasa Hikawa*, Shoko Kikkawa and Isao Azumaya*

Faculty of Pharmaceutical Sciences, Toho University, Funabashi, Chiba 274-8510, Japan
hidemasa.hikawa@phar.toho-u.ac.jp and isao.azumaya@phar.toho-u.ac.jp

**Table of contents**	**S1**
**General methods**	**S2**
**Scheme S1. General procedure and characterization of all compounds.**	**S2-8**
**Table S1. Effects of catalysts and solvents.**	**S9**
**Scheme S2. Reaction time course.**	**S10**
**Scheme S3. Comparison of reaction rates in heptane with the addition of H_2O (1 mmol) *vs.* D_2O (1 mmol).**	**S11**
**Scheme S4. Comparison of reaction rates in H_2O *vs.* in D_2O.**	**S12**
**Scheme S5. Crossover experiment.**	**S13-15**
**Scheme S6. Kinetic isotope effect.**	**S16**
**Scheme S7 Dehydrative benzylation of aniline.**	**S17**
**Scheme S8. Deuterium incorporation into the benzylic position.**	**S18-19**
**Scheme S9. Scale-up experiment.**	**S20**
**Copies of ^{1}H and ^{13}C NMR spectra of all compounds**	**S18-S42**

General Methods.

All of the starting materials and solvents were purchased from Sigma–Aldrich Japan, FUJIFILM Wako Pure Chemical Co., Nacalai Tesque, Inc., and TCI Co., Ltd. All commercially available reagents and solvents (guaranteed reagents) were used without further purification. CHROMATOREX Q-PACK SI50 (Fuji Silysia Chemical Ltd, Japan) was used for flash column chromatography. All melting points were determined using a Yanako micro melting point apparatus without correction. ^{1}H NMR (400 MHz) and ^{13}C NMR (100 MHz) spectra were recorded on a JEOL ECS400 spectrometer. IR spectra were measured with a JASCO FT/IR-4100 spectrometer. Mass spectra were obtained using a JEOL the JMS-700 MStation Mass Spectrometer.

Scheme S1. General procedures.

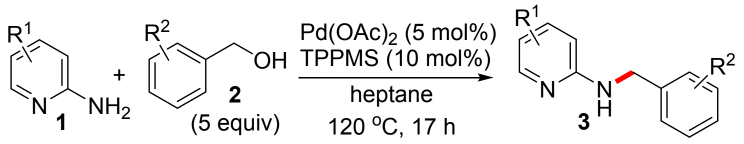

General procedure I: A mixture of 2-aminopyridines **1** (1 mmol), palladium(II) acetate (12 mg, 0.05 mmol), sodium diphenylphosphinobenzene-3-sulfonate (TPPMS, 36 mg, 0.1 mmol) and benzylic alcohols **2** (5 mmol) in heptane (4 mL) was heated at 120 °C for 17 h in a sealed tube under air. After cooling, the reaction mixture was poured into water and extracted with EtOAc. The organic layer was washed with brine, dried over $MgSO_4$ and concentrated in vacuo. The residue was purified by flash column chromatography (silica gel, hexane/EtOAc) to give desired product **3**.

General procedure II: A mixture of 2-aminopyridines **1** (1 mmol), palladium(II) acetate (24 mg, 0.1 mmol), sodium diphenylphosphinobenzene-3-sulfonate (TPPMS, 72 mg, 0.2 mmol) and benzylic alcohols **2** (5 mmol) in octane (4 mL) was heated at 150 °C for 24 h in a sealed tube under air. After cooling, the reaction mixture was poured into water and extracted with EtOAc. The organic layer was washed with brine, dried over $MgSO_4$ and concentrated in vacuo. The residue was purified by flash column chromatography (silica gel, hexane/EtOAc) to give desired product **3**.

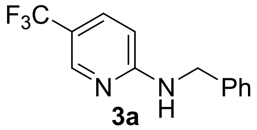

***N*-Benzyl-5-(trifluoromethyl)pyridin-2-amine 3a** [1]

A mixture of 2-amino-5-(trifluoromethyl)pyridine (**1a**) (1.13 g, 7.0 mmol), palladium(II) acetate (78.6 mg, 0.35 mmol), sodium diphenylphosphinobenzene-3-sulfonate (TPPMS, 254.7 mg, 0.7 mmol) and benzyl alcohol (**2a**) (5 mmol) in heptane (28 mL) was heated at 120 °C for 17 h in a sealed tube under air. After cooling, the reaction mixture was poured into water and extracted with EtOAc. The organic layer was washed with brine, dried over $MgSO_4$ and concentrated in vacuo. The residue was triturated with hexane to give desired product **3a** (1.56 g, 6.2 mmol, 88%) as a white solid.

mp 157–159 °C; IR (KBr) (cm^{-1}) 3230, 3030, 1618; ^{1}H-NMR (400 MHz, $CDCl_3$): δ 4.56 (d, J = 7.7 Hz, 2H), 5.26 (s, 1H), 6.40 (d, J = 8.9 Hz, 1H), 7.27–7.38 (m, 5H), 7.57 (dd, J = 8.8, 2.3 Hz, 4H), 8.35 (s, 1H); ^{13}C-NMR (100 MHz, $CDCl_3$) δ 46.1, 106.1, 115.8 (q, J_{CF} = 32.6 Hz), 124.5 (q, J_{CF} = 270.3 Hz), 127.4, 127.6, 128.8, 134.5 (q, J_{CF} = 2.9 Hz), 138.2, 146.1 (q, J_{CF} = 3.8 Hz), 160.2; MS (FAB): *m/z* 253 $[M+H]^+$.

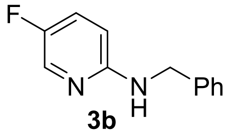

3b

***N*-Benzyl-5-fluoropyridin-2-amine 3b** [1]

Following the general procedure I, **3b** was obtained as a white solid. Yield 161 mg (79%); mp 101–102 °C; IR (KBr) (cm^{-1}) 3245, 3030, 1537, 1455 ; ^{1}H-NMR (400 MHz, $CDCl_3$): δ 4.47 (d, J = 5.7 Hz, 2H), 4.80 (s, 1H), 6.33 (dd, J = 8.9, 3.4 Hz, 1H), 7.18 (ddd, J = 8.5, 3.0, 0.9 Hz, 1H), 7.27–7.37 (m, 5H), 7.97 (d, J = 3.0 Hz, 1H); ^{13}C-NMR (100 MHz, $CDCl_3$) δ 46.8, 107.1 (d, J_{CF} = 3.8 Hz), 125.2 (d, J_{CF} = 20.1 Hz) 127.3 (d,J_{CF} = 7.7 Hz), 128.6, 134.6, 134.9, 139.0, 153.5 (d, J_{CF} = 241.5 Hz), 155.3 ; MS (FAB): *m/z* 203 $[M+H]^+$.

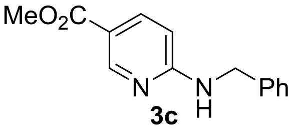

3c

Methyl-6-(benzylamino)nicotinate 3c [1]

Following the general procedure I, **3c** was obtained as a white solid. Yield 199 mg (82%); mp 156–158 °C; IR (KBr) (cm^{-1}) 3224, 2993, 1704, 1607; ^{1}H-NMR (400 MHz, $CDCl_3$): δ 3.87 (s, 3H), 4.58 (d, J = 6.0 Hz, 2H), 5.36 (s, 1H), 6.36 (dd, J = 0.7, 8.7 Hz, 1H), 7.27–7.38 (m, 5H), 7.99 (dd, J = 2.3, 8.7 Hz, 1H), 8.77 (dd, J = 0.5, 2.3 Hz, 1H); ^{13}C-NMR (100 MHz, $CDCl_3$) δ 46.1, 51.7, 105.7, 115.5, 127.4, 127.6, 128.8, 138.1, 138.6, 151.5, 160.7, 166.4; MS (FAB): *m/z* 243 $[M+H]^+$.

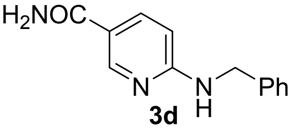

3d

6-(Benzylamino)nicotinamide 3d [1]

Following the general procedure I, **3d** was obtained as a white solid. Yield 170 mg (75%); mp 169–171 °C; IR (KBr) (cm^{-1}) 3399, 3203, 1644, 1604 ; ^{1}H-NMR (400 MHz, Methanol-d_4): δ 4.57 (s, 2H), 6.54 (dd, J = 0.7, 8.9 Hz, 1H), 7.20–7.25 (m, 1H), 7.28–7.35 (m, 4H), 7.87 (dd, J = 2.5, 8.8 Hz, 1H), 8.53 (dd, J = 0.7, 2.5 Hz, 1H); ^{13}C-NMR (100 MHz, Methanol-d_4) δ 46.1, 108.8, 118.7, 128.1, 128.4, 129.5, 137.7, 140.6, 149.8, 162.0, 171.1; MS (FAB): *m/z* 228 $[M+H]^+$.

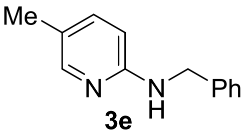

3e

***N*-Benzyl-5-methylpyridin-2-amine 3e** [1]

Following the general procedure I, **3e** was obtained as a white solid. Yield 191 mg (96%); mp 108–110 °C; IR (KBr) (cm^{-1}) 3235, 3027, 1611, 1536 ; ^{1}H-NMR (400 MHz, $CDCl_3$): δ 2.17 (s, 3H), 4.48 (d, J = 6.0 Hz, 1H), 4.74 (s, 1H), 6.32 (d, J = 8.5 Hz, 1H), 7.22–7.37 (m, 6H), 7.93–7.94 (m, 1H); ^{13}C-NMR (100 MHz, $CDCl_3$) δ 17.4, 46.5, 106.4, 121.9, 127.1, 127.3, 128.6, 138.5, 139.4, 147.7, 156.9; MS (FAB): *m/z* 199 $[M+H]^+$.

N-Benzylpyridin-2-amine 3f [1]

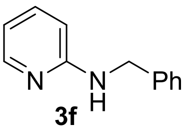

3f

Following the general procedure I, **3f** was obtained as a white solid. Yield 167 mg (90%); mp 91–93 °C; IR (KBr) (cm^{-1}) 3229, 3028, 1599, 1574 ; ^{1}H-NMR (400 MHz, $CDCl_3$): 4.51 (d, *J* = 5.7 Hz 2H), 4.88 (brs, 1H), 6.38 (d, *J* = 8.2 Hz, 1H), 6.59 (ddd, *J* = 0.9, 5.0, 7.1 Hz, 1H), 7.25-7.43 (m, 6H), 8.11 (ddd, *J* = 0.7, 1.8, 5.0 Hz, 1H); ^{13}C-NMR (100 MHz, $CDCl_3$) δ 46.3, 106.8, 113.1, 127.2, 127.4, 128.6, 137.5, 139.1, 148.2, 158.6; MS (FAB): *m/z* 185 $[M+H]^+$.

N-Benzyl-5-nitropyridin-2-amine 3g [2]

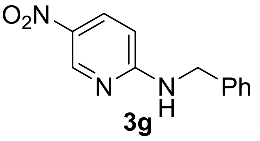

3g

Following the general procedure I, **3g** was obtained as a white solid. Yield 62 mg (27%); mp 129−131 °C; IR (KBr) (cm^{-1}) 3210, 3068, 2984, 1608 ; ^{1}H-NMR (400 MHz, $CDCl_3$): δ 4.64 (d, *J* = 5.3 Hz, 2H), 5.68 (s, 1H), 6.37 (dd, *J* = 9.2, 0.5 Hz, 3H), 7.30−7.39 (m, 5H), 8.20 (dd, *J* =9.3, 2.5 Hz, 1H), 9.03 (d, *J* = 2.5 Hz, 1H); ^{13}C-NMR (100 MHz, $CDCl_3$) δ 46.3, 127.5, 127.9, 128.9, 133.1, 136.1, 146.9, 161.0; MS (FAB): *m/z* 198 $[M+H]^+$.

6-Benzylaminonicotinicacid 3h [3]

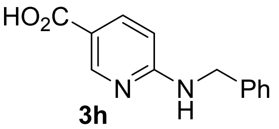

3h

Following the general procedure I, **3h** was obtained as a white solid. Yield 16 mg (7%) as a white solid; mp 214–222 °C; IR (KBr) (cm^{-1}) 3278, 1614, 1555 ; ^{1}H-NMR (400 MHz, $CDCl_3$): δ 4.58 (s, 2H), 6.55 (d, *J* = 8.9 Hz, 1H), 7.21 – 7.36 (m, 5H), 7.92 (dd, *J* = 8.9, 2.3Hz, 1H), 8.61 (dd, *J* = 2.2, 0.5 Hz, 1H); ^{13}C-NMR (100 MHz, $CDCl_3$) 46.1, 116.1, 128.1, 128.4, 129.5, 139.3, 140.4, 152.0, 162.4, 169.3 ; MS (FAB): *m/z* 229 $[M+H]^+$.

N-Benzylpyridin-3-amine 3i [1]

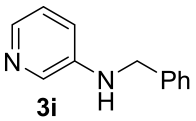

3i

Following the general procedure I, **3i** was obtained as a white solid. Yield 168 mg (88%); mp 87-89 °C; IR (KBr) (cm^{-1}) 3264, 3032, 1591, 1529 ; ^{1}H-NMR (400 MHz, $CDCl_3$): 4.15 (brs, 1H), 4.34 (s, 2H), 6.87 (ddd, *J* = 8.2, 3.0, 1.4 Hz, 1H), 7.07 (ddd, *J* = 8.5, 4.8, 0.7 Hz, 1H), 7.27-7.36 (m, 5H), 7.97 (dd, *J* = 4.7, 1.4 Hz, 1H), 8.07 (d, *J* = 2.8 Hz, 1H), ; ^{13}C-NMR (100 MHz, $CDCl_3$) δ 47.9, 118.6, 123.7, 127.4, 127.5, 128.8, 136.1, 138.5, 138.9, 144.0; MS (FAB): *m/z* 185 $[M+H]^+$.

N-Benzylquinolin-2-amine 3j [4]

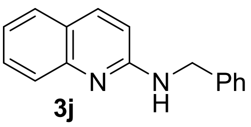

3j

Following the general procedure I, **3j** was obtained as a yellow solid. Yield 191 mg (82%); mp 98–100 °C; IR (KBr) (cm^{-1}) 3272, 3061, 1622, 1572 ; ^{1}H-NMR (400 MHz, $CDCl_3$): δ 4.73 (d, *J* = 5.5 Hz, 2H), 5.00 (brs, 1H), 6.63 (d, *J* = 8.9 Hz, 1H), 7.22 (ddd, *J* = 8.0, 6.9, 1.1 Hz, 1H), 7.26-7.43 (m, 5H), 7.54 (ddd, *J* = 8.5, 6.9, 1.6 Hz, 1H) 7.59 (dd, *J* = 8.0, 1.6 Hz, 1H), 7.71 (d, *J* = 8.5 Hz, 1H), 7.82 (d, *J* = 8.9 Hz, 1H); ^{13}C-NMR (100 MHz, $CDCl_3$) δ 45.9, 111.3, 122.2, 123.6, 126.2, 127.3, 127.4, 127.8, 128.6, 129.6, 137.4, 139.3, 148.0, 156.7; MS (FAB): *m/z* 235 $[M+H]^+$.

***N*-Benzylnicotinamide 3k** [5]

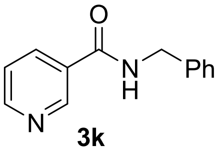

3k

Following the general procedure II, **3k** was obtained as a white solid. Yield 140.1 mg (66%); mp 70−72 ℃; IR (KBr) (cm^{-1}) 3286, 3030, 1634 ; ^{1}H-NMR (400 MHz, Methanol-d_4): δ 4.59 (s, 2H), 7.24 – 7.38 (m, 5H), 7.55 (ddd, *J* = 0.7, 4.8, 8.0 Hz, 1H), 8.27 (ddd, *J* = 3.9, 7.8, 8.0 Hz, 1H), 8.68 (dd, *J* = 1.6, 4.9 Hz, 1H), 9.00 (d, *J* = 1.6 Hz, 1H); ^{13}C-NMR (100 MHz, Methanol-d_4) δ 44.6, 125.2, 128.3, 128.7, 129.6, 132.0, 137.1, 139.8, 149.2, 152.7, 167.7; MS (FAB): *m/z* 213 [M+H]$^+$.

***N*-(4-Methoxybenzyl)-5-(trifluoromethyl)pyridin-2-amine 3l** [5]

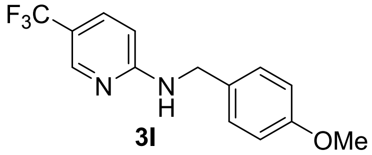

3l

Following the general procedure I, **3l** was obtained as a white solid. Yield 184 mg (65%); mp 173–175 ℃; IR (KBr) (cm^{-1}) 3229, 2910, 1620, 1579 ; ^{1}H-NMR (400 MHz, $CDCl_3$): δ 3.81 (s, 3H), 4.48 (d, *J* = 5.7 Hz, 2H), 5.19 (s, 1H), 6.39 (d, *J* = 8.7 Hz, 3H), 6.89 (d, *J* = 8.7 Hz, 2H), 7.27 (d, *J* = 8.7 Hz, 2H), 7.57 (dd, *J* = 2.3, 8.8 Hz, 1H), 8.34 (s, 1H); ^{13}C-NMR (100 MHz, $CDCl_3$) δ 45.6, 55.3, 106.0, 114.1, 115.7 (q, J_{CF} = 32.6 Hz), 124.6 (q, J_{CF} = 270.3 Hz), 128.8, 130.1, 134.5 (q, J_{CF} = 2.9 Hz), 146.1 (q, J_{CF} = 3.8 Hz), 159.1, 160.1; MS (FAB): *m/z* 283 [M+H]$^+$.

Methyl-6-(4-methoxybenzylamino)nicotinate 3m

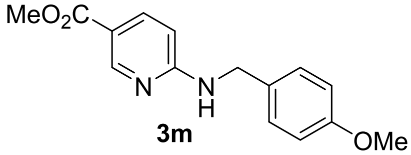

3m

Following the general procedure I, **3m** was obtained as a white solid. Yield 225 mg (83%); mp 137–139 ℃; IR (KBr) (cm^{-1}) 3227, 2854, 1714, 1615 ; ^{1}H-NMR (400 MHz, $CDCl_3$): δ 3.80 (s, 3H), 3.87 (s, 3H), 4.50 (d, *J* = 5.5 Hz, 2H), 5.26 (s, 1H), 6.36 (dd, *J* = 8.8, 0.7 Hz, 3H), 6.88 (d, *J* = 8.7 Hz, 2H), 7.27 (d, *J* = 8.7 Hz, 3H), 7.98 (dd, *J* = 8.7, 2.3, Hz, 1H), 8.77 (d, *J* = 1.8 Hz, 1H); ^{13}C-NMR (100 MHz, $CDCl_3$) δ 45.6, 51.6, 56.3, 105.7, 114.1, 115.4, 128.8, 130.1, 138.6, 151.5, 159.1, 160.7, 166.4; MS (FAB): *m/z* 273 [M+H]$^+$, HRMS (FAB): *m/z* [M+H]$^+$ calcd for $C_{15}H_{17}N_2O_3$ 273.1239; found 273.1239.

***N*-(4-Methoxybenzyl)pyridin-2-amine 3n** [1]

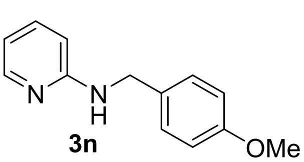

3n

Following the general procedure I, **3n** was obtained as a white solid. Yield 151 mg (70%); mp 126–128 ℃; IR (KBr) (cm^{-1}) 3234, 2951, 2835, 1603, 1573; ^{1}H-NMR (400 MHz, $CDCl_3$); δ 3.80 (s, 3H), 4.43 (d, *J* = 5.7 Hz, 2H), 4.79 (s, 1H), 6.37 (d, *J* = 8.2 Hz, 1H), 6.59 (ddd, *J* = 7.1, 5.0, 0.9 Hz, 1H), 6.88 (d, *J* = 8.7 Hz, 2H), 7.29 (d, *J* = 8.7 Hz, 3H), 7.40 (ddd, *J* = 8.6, 7.7, 2.1 Hz, 1H), 8.11 (s, 1H); ^{13}C-NMR (100 MHz, $CDCl_3$) 45.9, 55.4, 106.9, 113.2, 114.1, 128.8, 131.2, 137.5, 148.3, 158.7, 158.9 ; MS (FAB): *m/z* 215 [M+H]$^+$.

N-(4-Methoxybezyl)-5-methylpyridin-2-amine 3o [6]

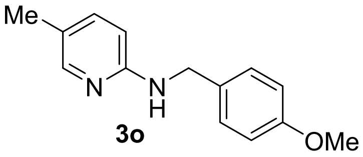
3o

Following the general procedure I, **3o** was obtained as a white solid. Yield 191 mg (84%); mp 144–146 ℃; IR (KBr) (cm^{-1}) 3235, 3007, 1614, 1534 ; ^{1}H-NMR (400 MHz, $CDCl_3$): δ 2.17 (s, 3H), 3.80 (s, 3H), 4.40 (d, J = 5.7 Hz, 2H), 4.66 (s, 1H), 6.31 (d, J = 8.5 Hz, 1H), 6.87 (d, J = 8.7 Hz, 2H), 7.24 (dd, J = 8.5, 2.3 Hz, 1H), 7.28 (d, J = 8.7 Hz, 2H), 7.93 (dd, J = 1.6, 0.7 Hz, 1H); ^{13}C-NMR (100 MHz, $CDCl_3$) δ 17.4, 46.0, 56.3, 106.4, 114.0, 121.8, 128.6, 131.4, 138.5, 147.7, 156.9, 158.8; MS (FAB): *m/z* 229 $[M+H]^+$.

6-(4-Methoxybenzylamino)nicotinamide 3p [8]

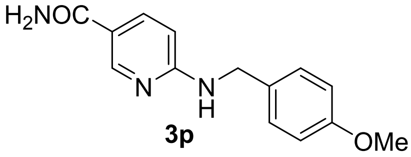
3p

Following the general procedure I (3 mmol of 4-methoxybenzyl alcohol was used), **3p** was obtained as a white solid. Yield 179 mg (70%); mp 214−216 ℃; IR (KBr) (cm^{-1}) 3481, 3413, 3192, 1668, 1651, 1606 ; ^{1}H-NMR (400 MHz, Methanol-d_4): δ 3.77 (s, 3H), 4.48 (s, 2H), 6.52 (d, J = 8.9, 1H), 6.87 (d, J = 8.7 Hz, 2H), 7.26 (d, J = 8.5, 2H), 7.86 (dd, J = 2.3, 8.9 Hz, 1H), 8.53 (d, J = 2.5 Hz, 1H); ^{13}C-NMR (100 MHz, Methanol-d_4) δ 45.6, 55.7, 108.7, 114.9, 118.6, 129.7, 132.4, 137.7, 149.8, 160.4, 162.0, 171.1; MS (FAB): *m/z* 258 $[M+H]^+$.

N-(4-Methylbenzyl)-5-(trifluoromethyl)pyridin-2-amine 3q [9]

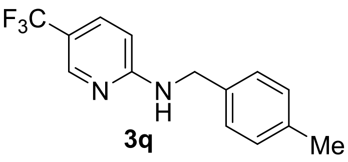
3q

Following the general procedure I, **3q** was obtained as a white solid. Yield 245 mg (92%); mp 190–193 ℃; IR (KBr) (cm^{-1}) 3236, 3050, 1620 ; ^{1}H-NMR (400 MHz, $CDCl_3$): δ 2.35 (s, 3H), 4.50 (d, J = 5.7 Hz, 2H), 5.25 (s, 1H), 6.39 (d, J = 8.7 Hz, 1H), 7.16 (d, J = 8.0 Hz, 2H), 7.22 (d, J = 8.2 Hz, 1H), 7.57 (dd, J = 8.9, 2.5 Hz, 1H) 8.33 (s, 1H); ^{13}C-NMR (100 MHz, $CDCl_3$) δ 21.1, 45.9, 106.0, 115.7 (q, J_{CF} = 32.6 Hz), 124.6 (q, J_{CF} = 270.3 Hz), 127.4, 129.5, 134.5 (q, J_{CF} = 2.9 Hz), 135.1, 137.3, 146.1 (q, J_{CF} = 4.8 Hz), 160.2; MS (FAB): *m/z* 267 $[M+H]^+$.

N-(3-Methylbenzyl)-5-(trifluoromethyl)pyridin-2-amine 3r [1]

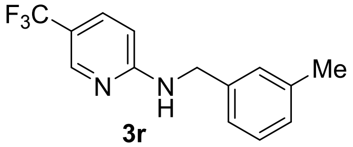
3r

Following the general procedure I, **3r** was obtained as a white solid. Yield 247 mg (93%); mp 113–115 ℃; IR (KBr) (cm^{-1}) 3244, 2855, 1621, 1573; ^{1}H-NMR (400 MHz, $CDCl_3$): δ 2.35 (s, 3H), 4.51 (d, J = 5.7 Hz, 2H), 5.26 (s, 1H), 6.40 (d, J = 8.7 Hz, 1H), 7.10–7.16 (m, 3H), 7.23 (d, J = 7.6 Hz, 1H), 7.57 (dd, J = 8.8, 2.5 Hz, 1H) 8.34 (s, 1H); ^{13}C-NMR (100 MHz, $CDCl_3$) δ 21.4, 46.1, 106.0, 115.7 (q, J_{CF} = 33.6 Hz), 124.4, 124.6 (q, J_{CF} = 270.3), 128.1, 128.3, 128.7, 134.5 (q, J_{CF} = 2.9 Hz), 138.1, 138.5, 146.1 (q, J_{CF} = 3.8 Hz), 160.2; MS (FAB): *m/z* 267 $[M+H]^+$

N-(2-Methoxybenzyl)-5-(trifluoromethyl)pyridin-2-amine 3s

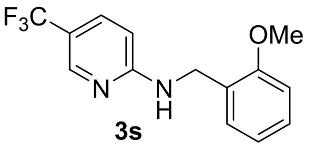

3s

Following the general procedure I, **3s** was obtained as a white solid. Yield 238 mg (84%); mp 116–118 ℃; IR (KBr) (cm^{-1}) 3242, 2941, 1617; ^{1}H-NMR (400 MHz, $CDCl_3$): δ 3.87 (s, 3H), 4.54 (d, J = 6.0 Hz, 2H), 5.38 (s, 1H), 6.41 (d, J = 8.9 Hz, 1H), 6.89–6.94 (m, 2H), 7.28 (d, J = 7.6 Hz, 2H), 7.55 (dd, J = 8.7, 2.5 Hz, 1H), 8.33 (s, 1H); ^{13}C-NMR (100 MHz, $CDCl_3$) δ 41.6, 56.3, 106.0, 110.3, 115.3 (q, J_{CF} = 32.6 Hz), 120.5, 124.6 (q, J_{CF} = 270.3 Hz), 126.1, 128.8, 128.9, 134.3 (q, J_{CF} = 3.8 Hz), 146.1 (q, J_{CF} = 4.8 Hz), 157.5, 160.4; MS (FAB): m/z 283 $[M+H]^+$, HRMS (FAB): m/z $[M+H]^+$ calcd for $C_{14}H_{14}F_3N_2O$ 283.1058; found 283.1057.

N-(4-Fluorobenzyl)-5-(trifluoromethyl)pyridin-2-amine 3t

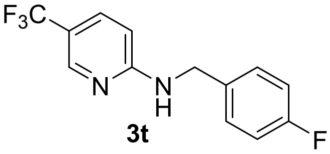

3t

Following the general procedure II, **3t** was obtained as a white solid. Yield 228 mg (84%); mp 119–120 °C; IR (KBr) (cm^{-1}): 3229, 1614, 1511; ^{1}H-NMR (400 MHz, $CDCl_3$): δ 4.54 (d, J = 5.7 Hz, 2H), 5.28 (brs, 1H), 6.39 (d, J = 8.7 Hz, 1H), 7.03 (tdd, J = 8.7, 3.0, 2.1 Hz, 2H), 7.27–7.34 (m, 2H), 7.58 (dd, J = 8.8, 2.3 Hz, 1H), 8.33–8.34 (m, 1H); ^{13}C-NMR (100 MHz, $CDCl_3$): δ 45.3, 106.3, 115.6 (d, J_{CF} = 22.0 Hz), 116.0 (q, J_{CF} = 32.6 Hz), 124.5 (q, J_{CF} = 270.3 Hz), 129.0 (d, J_{CF} = 7.7 Hz), 134.0 (d, J_{CF} = 3.8 Hz), 134.5 (q, J_{CF} = 2.9 Hz), 146.1 (q, J_{CF} = 4.8 Hz), 160.0, 162.2 (d, J_{CF} = 245.4 Hz); HRMS (FAB): m/z $[M+H]^+$ calcd for $C_{13}H_{11}F_4N_2$ 271.0858; found 271.0858.

N-(3-Fluorobenzyl)-5-(trifluoromethyl)pyridin-2-amine 3u

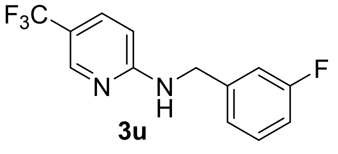

3u

Following the general procedure II, **3u** was obtained as a white solid. Yield 153 mg (56%); mp 103–104 °C; IR (KBr) (cm^{-1}): 3235, 1618, 1329; ^{1}H-NMR (400 MHz, $CDCl_3$): δ 4.58 (d, J = 6.0 Hz, 2H), 5.32 (brs, 1H), 6.40 (d, J = 8.9 Hz, 1H), 6.97 (dddd, J = 8.7, 8.2, 2.5, 0.5 Hz, 1H), 7.05 (ddd, J = 9.6, 2.3, 1.6 Hz, 1H), 7.12 (dd, J = 7.6, 0.7 Hz, 1H), 7.31 (ddd, J = 8.0, 8.0, 6.0 Hz, 1H), 7.58 (dd, J = 8.8, 2.5 Hz, 1H), 8.34-8.35 (m, 1H); ^{13}C-NMR (100 MHz, $CDCl_3$): δ 45.4, 106.3, 114.2 (d, J_{CF} = 22.0 Hz), 114.4 (d, J_{CF} = 21.1 Hz), 116.1 (q, J_{CF} = 33.6 Hz), 122.8 (d, J_{CF} = 2.9 Hz), 124.5 (d, J_{CF} = 270.3 Hz), 130.3 (d, J_{CF} = 8.6 Hz), 134.5 (q, J_{CF} = 2.9 Hz), 141.1 (d, J_{CF} = 6.7 Hz), 146.1 (q, J_{CF} = 3.8 Hz), 160.0, 163.1 (d, J_{CF} = 246.3 Hz); HRMS (FAB): m/z $[M+H]^+$ calcd for $C_{13}H_{11}F_4N_2$ 271.0858; found:271.0857.

5-Fluoro-N-(4-fluorobenzyl)pyridin-2-amine 3v [10]

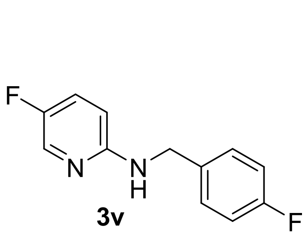

3v

Following the general procedure II, **3v** was obtained as a white solid. Yield 195 mg (88%); mp 90–91 °C; IR (KBr) (cm^{-1}) 3246, 1535, 1233; ^{1}H-NMR (400 MHz, $CDCl_3$): δ 4.45 (d, J = 6.0 Hz, 2H), 4.77 (brs, 1H), 6.32 (dd, J = 9.2, 3.4 Hz, 1H), 7.02 (tdd, J = 8.7, 3.0, 2.1 Hz, 2H), 7.19 (ddd, J = 8.9, 8.0, 3.0 Hz, 1H),

7.31 (dd, J = 8.4, 5.3 Hz, 2H), 7.97 (d, J = 3.0 Hz, 1H); ^{13}C-NMR (100 MHz, $CDCl_3$): δ 46.1, 107.2 (d, J_{CF} = 3.8 Hz), 115.5 (d, J_{CF} = 21.1 Hz), 125.3 (d, J_{CF} = 20.1 Hz), 129.0 (d, J_{CF} = 7.7 Hz), 134.8 (d, J_{CF} = 24.9 Hz), 134.8 (d, J_{CF} = 3.8 Hz), 153.6 (d, J_{CF} = 241.5 Hz), 155.1, 162.1 (d, J_{CF} = 245.4 Hz); HRMS (FAB): m/z $[M+H]^+$ calcd for $C_{12}H_{11}F_2N_2$ 221.089; found 221.089.

References

1. H. Hikawa, H. Imamura, S. Kikkawa, I. Azumaya, *Green Chem.* **2018**, *20*, 3044.
2. A. V. Gulevskaya, B. U. W. Maes, C. Meyers, W. A. Herrebout, B. J. van der Veken, *Eur. J. Org. Chem.* **2006**, 5305-5314.
3. P. J. Skinner, M. C. Cherrier, P. J. Webb, C. R. Sage, H. T. Dang, C. C. Pride, R. Chen, S. Y. Tamura, J. G. Richman, D. T. Connolly, G. Semple, *Bioorg. Med. Chem. Lett.* **2007**, *17*, 6619.
4. H. Hikawa, R. Tan, A. Tazawa, S. Kikkawa, I. Azumaya, *Eur. J. Org. Chem.* **2020**, 539.
5. J. Das, D. Banerjee, *J. Org. Chem.* **2018**, *83*, 3378-3384.
6. alsh, H. F. Sneddon, C. J. Moody, *ChemSusChem* **2013**, *6*, 1455.
7. B. E. Fink, L. Chen, P. Chen, D. S. Dodd, A. V. Gavai, S.-H. Kim, W. Vaccaro, L. H. Zhang, Y. Zhao, **2009**, WO 2009100375 20090813.
8. Y. Hoshimoto, T. Kinoshita, S. Hazra, M. Ohashi, S. Ogoshi, *J. Am. Chem. Soc.* **2018**, *140*, 7292.
9. N. Kuboyama, K. Koizumi, O. Yamashita, O. Wakabayashi, K. Tomono, T. Hattori, **1999**, JP 11246528 A 19990914.
10. Z.-W. Xi, L. Yang, D.-Y. Wang, C.-D. Pu, Y.-M. Shen, C.-D. Wu, X.-G. Peng, *J. Org. Chem.* **2018**, *83*, 11886.

Table S1. Effects of catalysts and solvents.

A mixture of 5-(trifluoromethyl)pyridin-2-amine **1a** (162 mg, 1 mmol), Pd(OAc)$_2$ (11 mg, 0.05 mmol), sodium diphenylphosphinobenzene-3-sulfonate (TPPMS, 36 mg, 0.1 mmol), and benzyl alcohol **2a** (515 µL, 5 mmol), in heptane (4 mL) was heated at 100 °C for 4 h in a sealed tube under air. After the reaction mixture was cooled, 1,3,5-trimethoxybenzene (168 mg, 1 mmol, internal standard) and EtOAc were added to the reaction mixture. The organic layer was concentrated in vacuo. The residue was analyzed by ^{1}H-NMR spectroscopy.

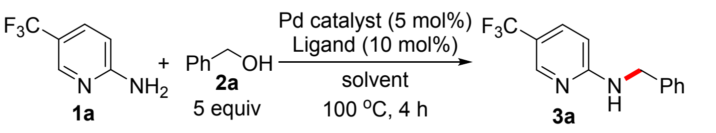

Entry	Pd catalyst	Ligand	Solvent	NMR Yield
1	Pd(OAc)$_2$	**L1**	heptane	quant
2	Pd(OAc)$_2$	**L1**	heptane	0 [b]
3	Pd(TFA)$_2$	**L1**	heptane	97
4	PdCl$_2$	**L1**	heptane	0
5 [c]	Pd$_2$(dba)$_3$·CHCl$_3$ [d]	**L1**	heptane	77
6	Pd(OAc)$_2$	**L1**	octane	quant
7	Pd(OAc)$_2$	**L1**	hexane	2
8	Pd(OAc)$_2$	**L1**	MCH [e]	38
9	Pd(OAc)$_2$	**L1**	toluene	4
10	Pd(OAc)$_2$	**L1**	(CHCl$_2$)$_2$	0
11	Pd(OAc)$_2$	**L1**	1,4-dioxane	20
12	Pd(OAc)$_2$	**L1**	none	8
13	Pd(OAc)$_2$	**L2**	heptane	5
14	Pd(OAc)$_2$	**L3**	heptane	0
15	Pd(OAc)$_2$	**L4**	heptane	6
16	Pd(OAc)$_2$	none	heptane	30
17 [f]	Pd(OAc)$_2$	**L1**	heptane	99

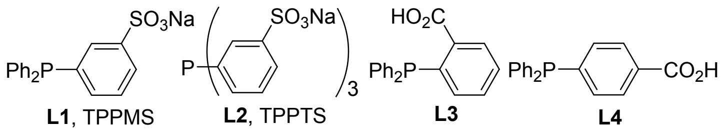

[a] Reaction conditions: **1a** (1 mmol), catalyst (5 mol%), TPPMS (10 mol%), **2a** (5 equiv), solvent (4 mL), 100 °C, 4 h under air. [b] Alcohol **2a** (3 equiv) was used. [c] Conducted at 120 °C for 16 h. [d] 2.5 mol%. [e] Methylcyclohexane. [f] Under Ar.

Scheme S2. Reaction time course.

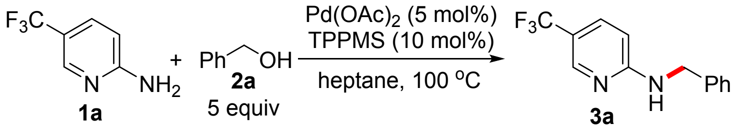

A mixture of 5-(trifluoromethyl)pyridin-2-amine **1a** (162 mg, 1 mmol), $Pd(OAc)_2$ (11 mg, 0.05 mmol), sodium diphenylphosphinobenzene-3-sulfonate (TPPMS, 36 mg, 0.1 mmol), benzyl alcohol **2a** (515 μL, 5 mmol), and 1,3,5-trimethoxybenzene (168 mg, 1 mmol, internal standard) in heptane (4 mL) was heated at 100 °C in a sealed tube under air. Every hour, a few drops of the reaction mixture were transferred into a test tube. The sample was extracted with $CDCl_3$, which was analyzed by ^{1}H-NMR spectroscopy.

Time (h)	[**1a**]	[**3a**]	[**4a**]
0	0.25	0	0
1	0.25	0	0.015
2	0.24	0.01	0.035
3	0.17	0.085	0.103
4	0	0.25	0.208

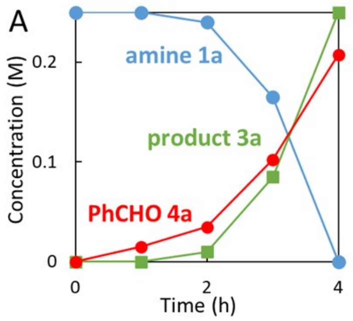

Time (h)	[**2a**]	ln [**2a**]/[**2a**]$_0$
0	1.25	0
1	1.24	-0.008032172
2	1.22625	-0.019182819
3	0.99625	-0.2269006
4	0.59	-0.750776293
5	0.46125	-0.996958635

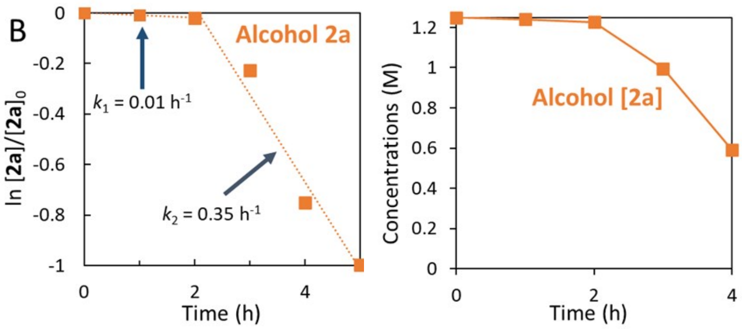

Scheme S3. Comparison of reaction rates in heptane with the addition of H_2O (1 mmol) *versus* D_2O (1 mmol).

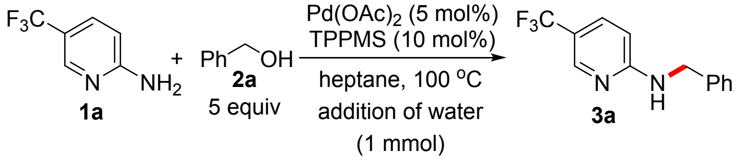

A mixture of 5-(trifluoromethyl)pyridin-2-amine **1a** (162 mg, 1 mmol), $Pd(OAc)_2$ (11 mg, 0.05 mmol), sodium diphenylphosphinobenzene-3-sulfonate (TPPMS, 36 mg, 0.1 mmol), benzyl alcohol **2a** (515 μL, 5 mmol), 1,3,5-trimethoxybenzene (168 mg, 1 mmol, internal standard), and H_2O (18 mg, 1 mmol) or D_2O (20 mg, 1 mmol) in heptane (4 mL) was heated at 100 ^{o}C in a sealed tube under air. Every hour, a few drops of the reaction mixture were transferred into a test tube. The sample was extracted with $CDCl_3$, which was analyzed by ^{1}H-NMR spectroscopy.

Addition of H_2O (1 mmol)

Time (h)	[**2a**]	ln [**2a**]/[**2a**]$_0$
0	1.25	0
1	1.0725	-0.153151179
2	0.93125	-0.294371061
3	0.7375	-0.527632742
4	0.575	-0.776528789
5	0.46125	-0.996958635

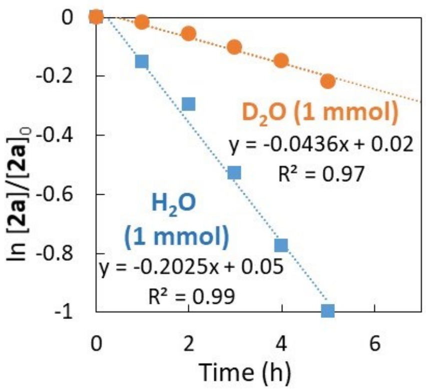

Addition of D_2O (1 mmol)

Time (h)	[**2a**]	ln [**2a**]/[**2a**]$_0$
0	1.25	0
1	1.23	-0.016129382
2	1.18125	-0.056570351
3	1.1275	-0.103140759
4	1.07875	-0.147340588
5	1.00625	-0.216913002

KSIE (k_{H_2O}/k_{D_2O}) = 0.2025/0.0436 =4.6

Scheme S4. Comparison of reaction rates in H_2O *versus* in D_2O.

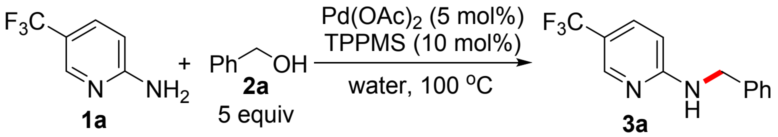

A mixture of 5-(trifluoromethyl)pyridin-2-amine **1a** (162 mg, 1 mmol), $Pd(OAc)_2$ (11 mg, 0.05 mmol), sodium diphenylphosphinobenzene-3-sulfonate (TPPMS, 36 mg, 0.1 mmol), benzyl alcohol **2a** (515 μL, 5 mmol), and 1,3,5-trimethoxybenzene (168 mg, 1 mmol, internal standard) in H_2O or D_2O (4 mL) was heated at 100 °C in a sealed tube under air. Every hour, a few drops of the reaction mixture were transferred into a test tube. The sample was extracted with $CDCl_3$, which was analyzed by ^{1}H-NMR spectroscopy.

in H_2O

Time (h)	[**2a**]	ln [**2a**]/[**2a**]$_0$
0	1.25	0
1	1.175	-0.061875404
2	1.0275	-0.196014884
3	0.88125	-0.349557476
4	0.815	-0.427710717
5	0.74125	-0.52256088
6	0.65875	-0.64055473
7	0.56375	-0.796287939

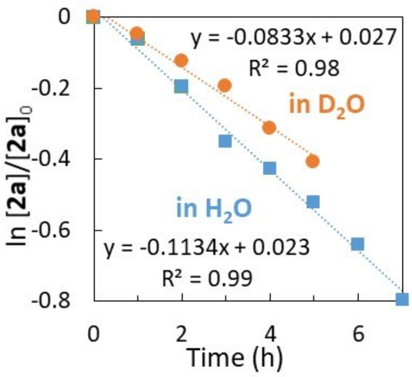

in D_2O

Time (h)	[**2a**]	ln [**2a**]/[**2a**]$_0$
0	1.25	0
1	1.19125	-0.048140375
2	1.105	-0.123298216
3	1.0275	-0.196014884
4	0.91375	-0.313341819
5	0.83	-0.40947313

KSIE (k_{H_2O}/k_{D_2O}) = 0.1134/0.0833 =1.4

Scheme S5. Crossover experiment.

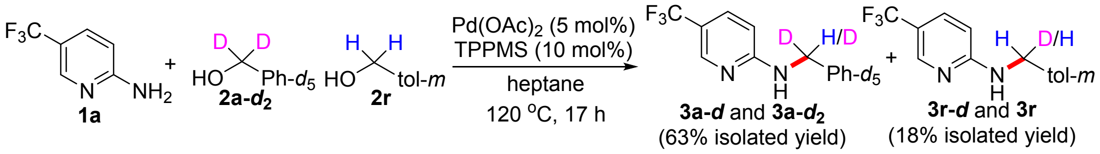

A mixture of 5-(trifluoromethyl)pyridin-2-amine **1a** (162 mg, 1 mmol), $Pd(OAc)_2$ (11 mg, 0.05 mmol), TPPMS (36 mg, 0.1 mmol), benzylalcohol-d_7 **2a-*d*₂** (290 mg, 2.5 mmol), 3-methylbenzyl alcohol **2r** (305 mg, 2.5 mmol) in heptane (4 mL) was heated at 120 °C for 17 h in a sealed tube under air. After cooling, the reaction mixture was poured into water and extracted with EtOAc. The organic layer was washed with brine, dried over $MgSO_4$ and concentrated in vacuo. The residue was purified by flash column chromatography (silica gel, hexanes/EtOAc) to give *N*-benzyl-5-(trifluoromethyl)pyridin-2-amines (**3a-*d*** and **3a-*d*₂** mixture) and *N*-(3-methylbenzyl)-5-(trifluoromethyl)pyridin-2-amines (**3r** and **3r-*d*** mixture) in 63% and 18% yield, respectively.

N-(3-Methylbenzyl)-5-(trifluoromethyl)pyridin-2-amines (**3r** and **3r-*d*** mixture)		
Signal δ	4.5 (d, *J*=5.6 Hz, 2H): methylene	6.4 (d, *J*=8.8 Hz, 1H)
Integral value	1.65	1.00
	[**3r-*d***] = x, [**3r**] = 1-x, x+ 2(1-x) = 1.65, x=0.35	

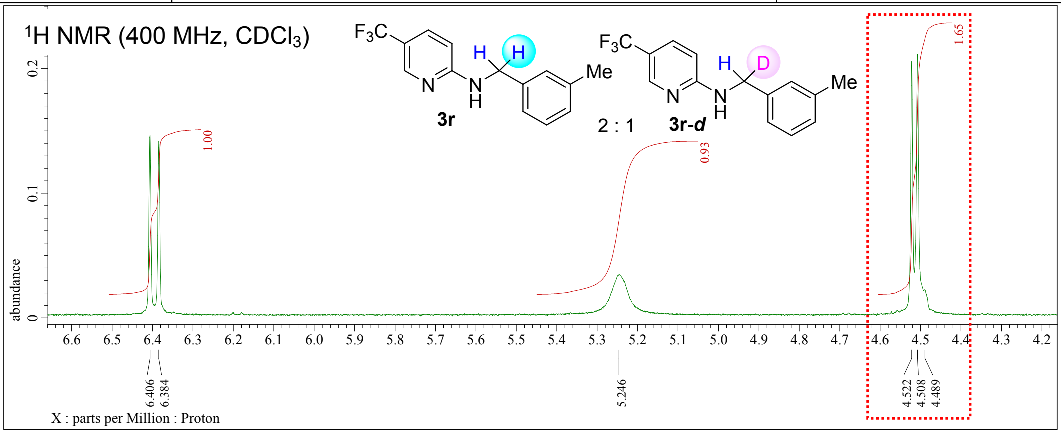

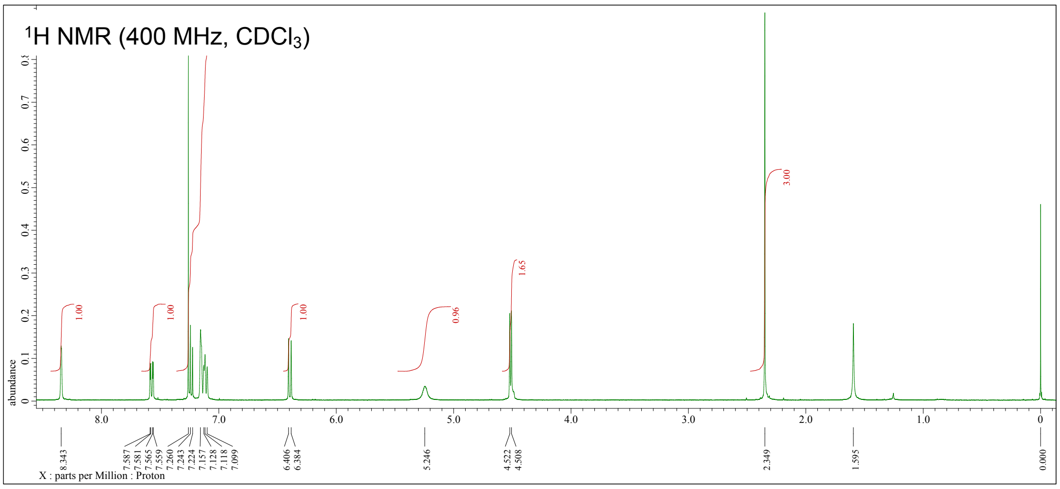

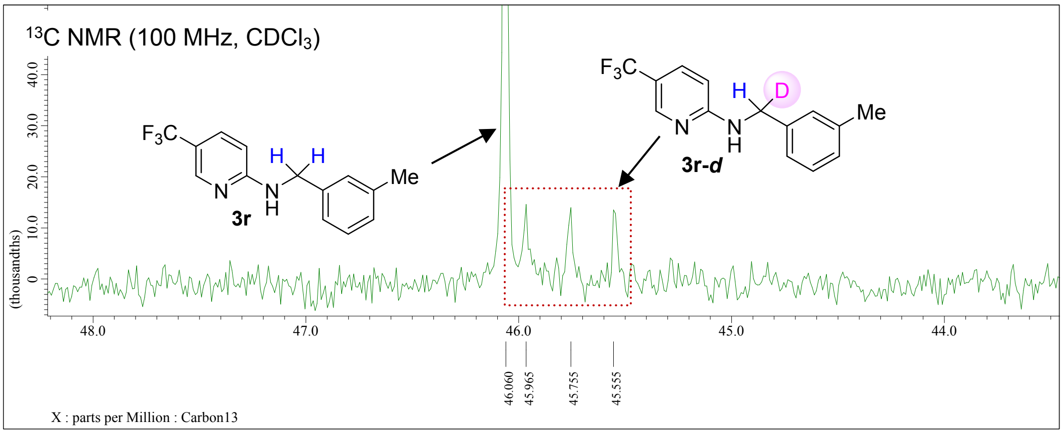

^{13}C NMR (100 MHz, CDCl$_3$)
F$_3$C
N
N
H
H
H
Me
3r
F$_3$C
N
N
H
H
D
Me
3r-*d*
(thousandths)
40.0
30.0
20.0
10.0
0
48.0
47.0
46.0
45.0
44.0
46.060
45.965
45.755
45.555
X : parts per Million : Carbon13

N-Benzyl-5-(trifluoromethyl)pyridin-2-amines (**3a-*d*** and **3a-*d*$_2$** mixture)		
Signal δ	4.5-4.6 (m, 1H): methylene	6.4 (d, *J*=8.4 Hz, 1H)
Integral value	0.81	1.00
	[**3a-*d***] = 0.81, [**3a-*d*$_2$**] = 1-0.81 = 0.19	

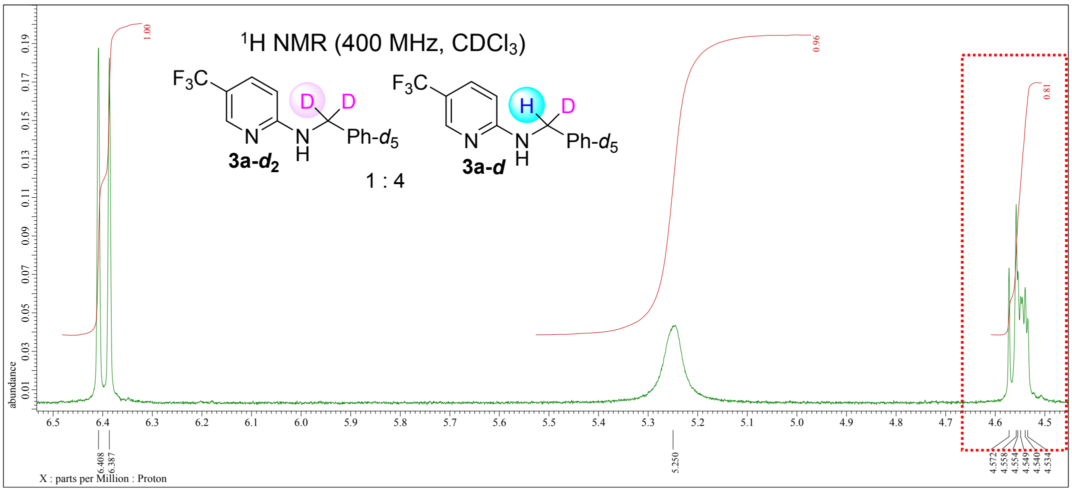

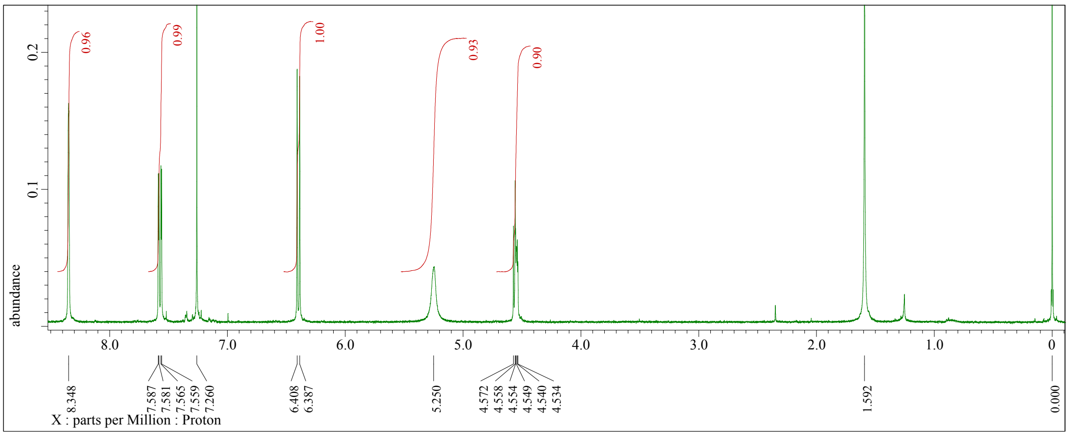

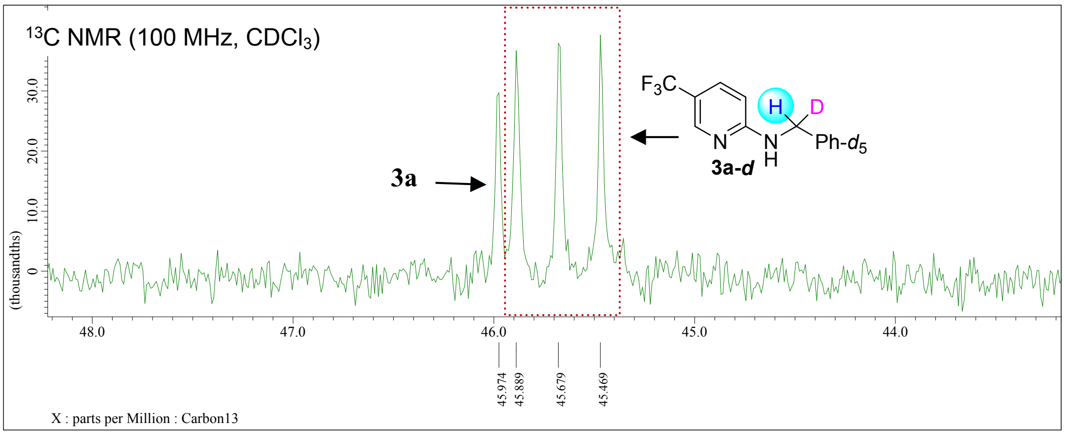

Scheme S6. Kinetic isotope effect.

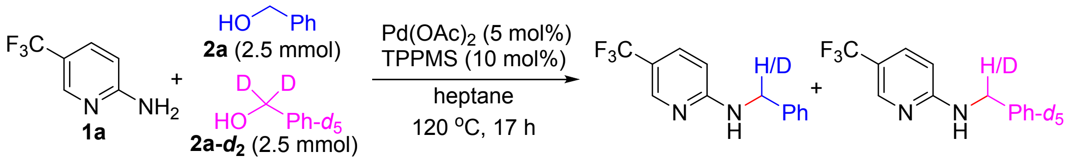

A mixture of 5-(trifluoromethyl)pyridin-2-amine **1a** (162 mg, 1 mmol), $Pd(OAc)_2$ (11 mg, 5 mol%), TPPMS (36 mg, 10 mol%), benzyl alcohol **2a** (271 mg, 2.5 mmol), and benzyl-d_7 alcohol **2a-*d*$_2$** (293 mg, 2.5 mmol) in H_2O (4 mL) was heated at 120 °C for 17 h in a sealed tube under air. After cooling, the reaction mixture was poured into water and extracted with EtOAc. The organic layer was washed with brine, dried over $MgSO_4$ and concentrated in vacuo. The residue was purified by flash column chromatography (silica gel, hexane/EtOAc). The product was analyzed by ^{1}H-NMR spectroscopy.

Signal δ 7.18-7.38 (Phenyl-**5H** and pyridine-**H**: total **6H**), 3.77	6.64 (pyridine-**H**), 1.00
3.77 (Ph-**5H**), 5.00-3.77 = 1.23 (Ph-**5D**), **KIE = 3.77 / 1.23 = 3.1**	

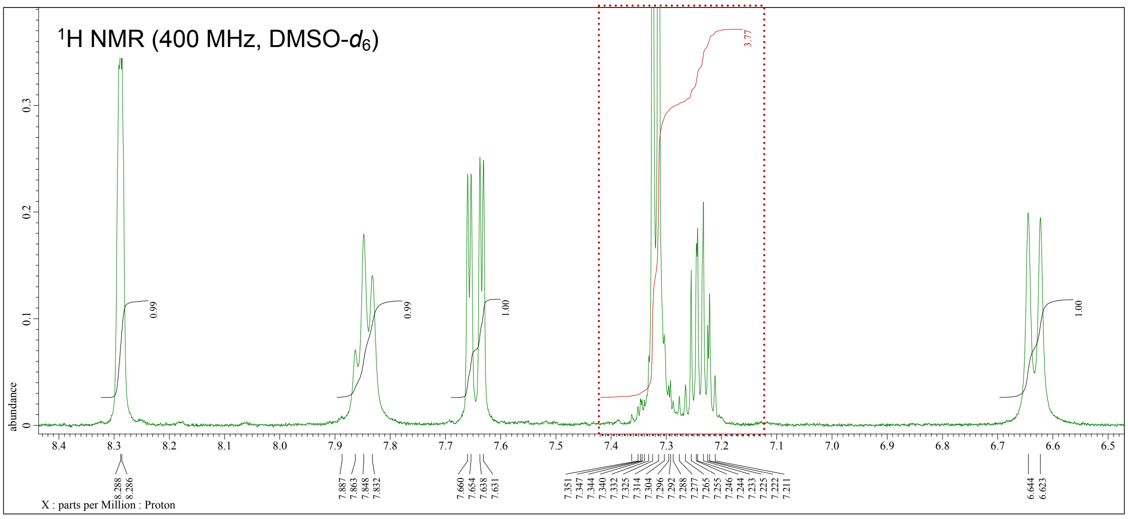

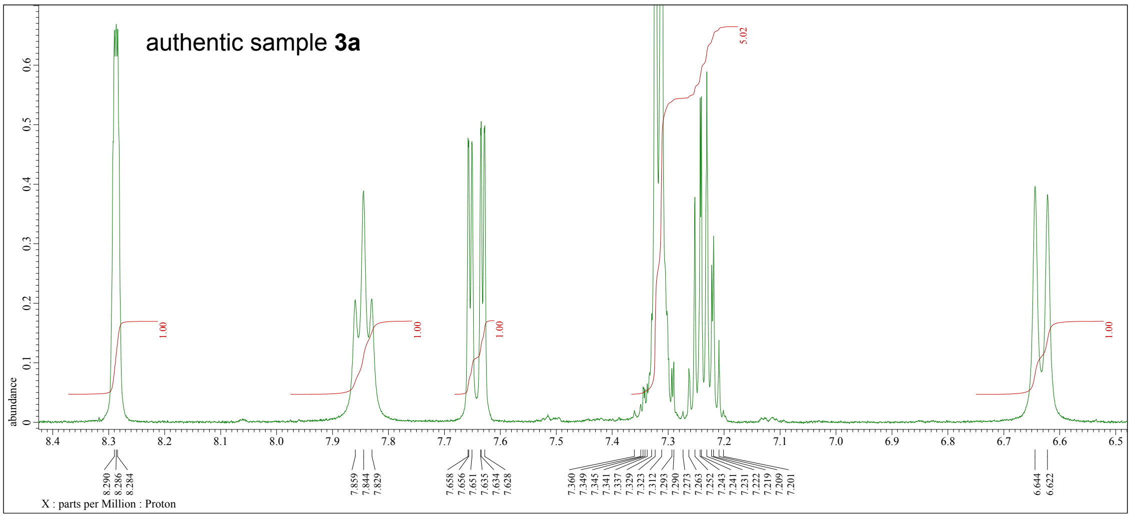

Scheme S7. Dehydrative benzylation of aniline.

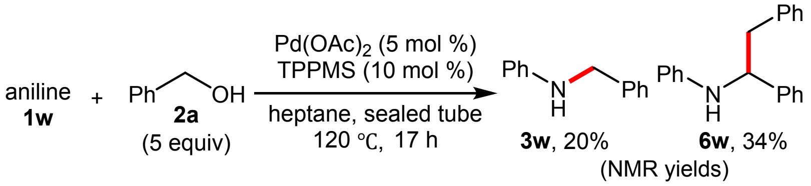

A mixture of aniline (**1w**) (93 mg, 1 mmol), $Pd(OAc)_2$ (11 mg, 0.05 mmol), sodium diphenylphosphinobenzene-3-sulfonate (TPPMS, 36 mg, 0.1 mmol), and benzyl alcohol **2a** (515 µL, 5 mmol), in heptane (4 mL) was heated at 120 °C for 17 h in a sealed tube under air. After the reaction mixture was cooled, 1,3,5-trimethoxybenzene (168 mg, 1 mmol, internal standard) and EtOAc were added to the reaction mixture. The organic layer was concentrated in vacuo. The residue was analyzed by ^{1}H-NMR spectroscopy.

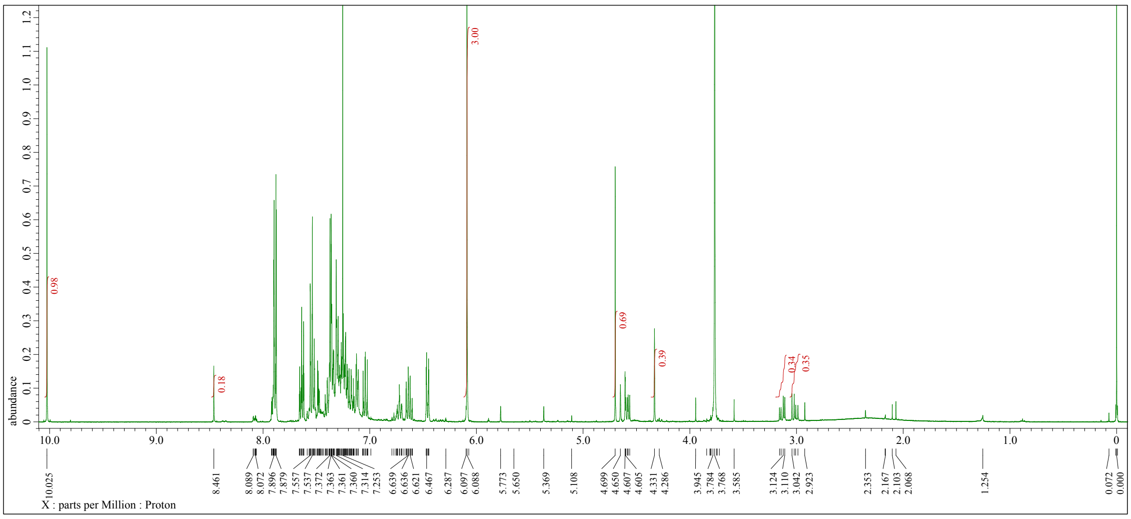

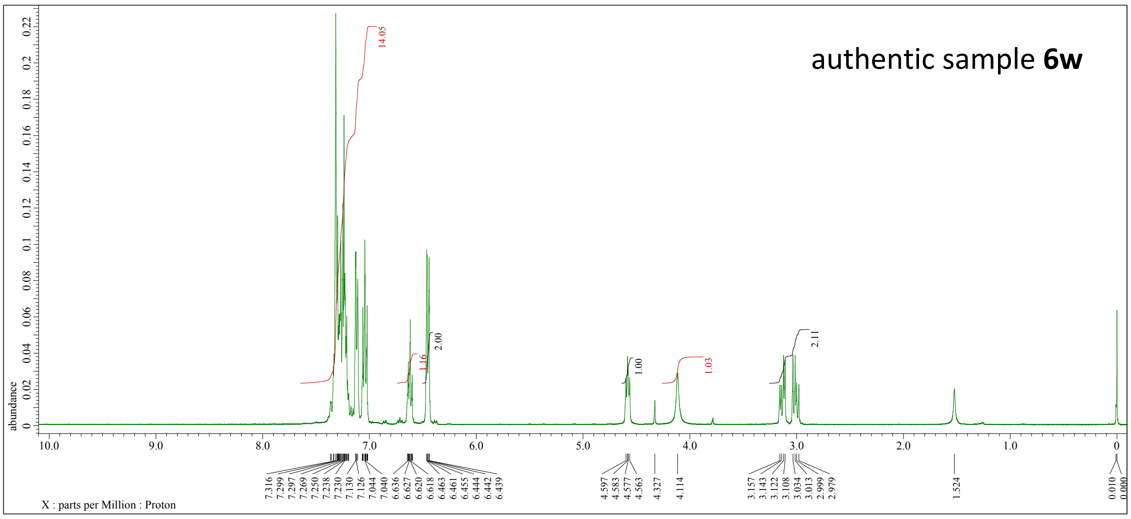

Scheme S8. Deuterium incorporation into the benzylic position.

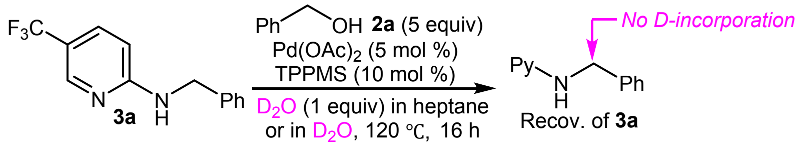

1. *Addition of D_2O (1 mmol) in heptane.*

A mixture of N-benzylated substrate (**3a**) (252 mg, 1 mmol), $Pd(OAc)_2$ (11 mg, 0.05 mmol), sodium diphenylphosphinobenzene-3-sulfonate (TPPMS, 36 mg, 0.1 mmol), and benzyl alcohol **2a** (515 µL, 5 mmol), D_2O (20 mg, 1 mmol) in heptane (4 mL) was heated at 120 °C for 16 h in a sealed tube under air. After the reaction mixture was cooled, the organic layer was concentrated in vacuo. The residue was analyzed by ^{1}H-NMR spectroscopy.

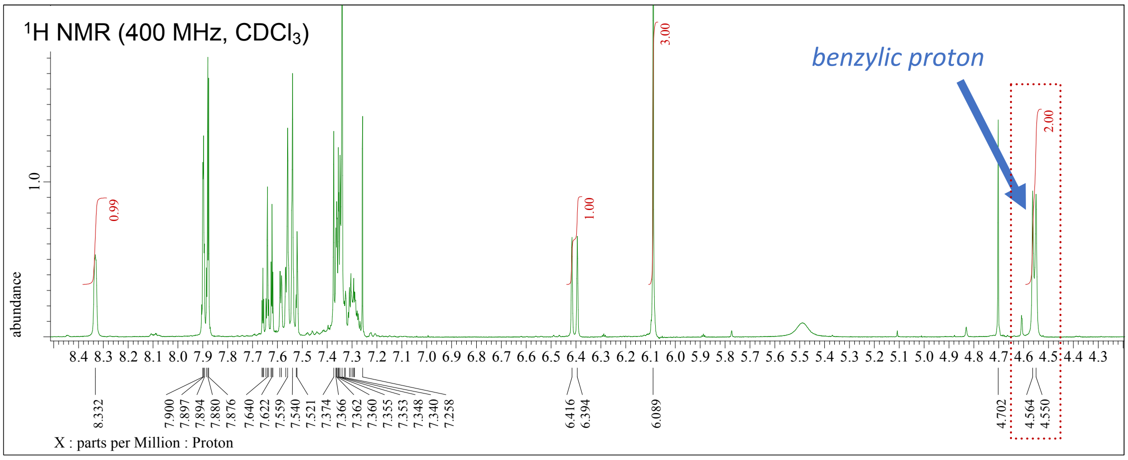

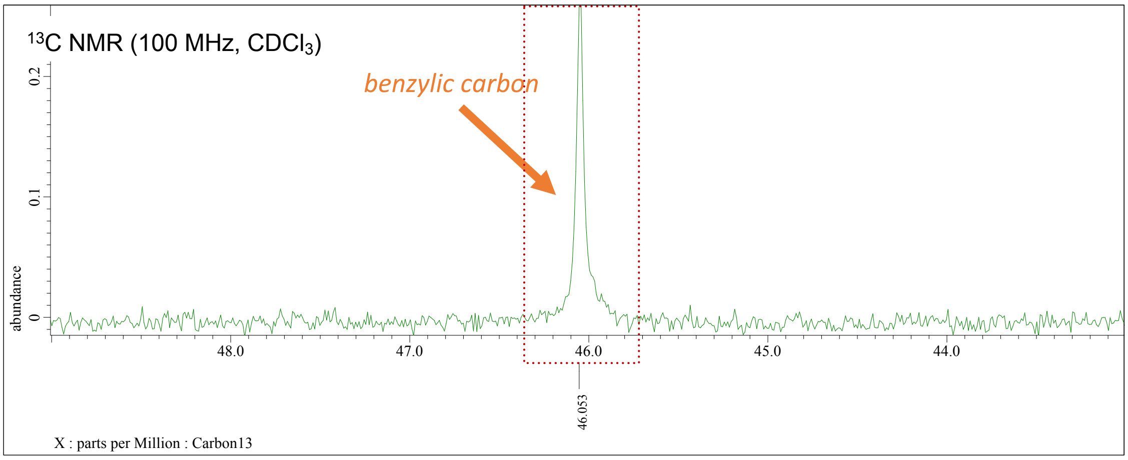

2. *The reaction in D_2O (4 mL) as a solvent.*

A mixture of N-benzylated substrate (**3a**) (252 mg, 1 mmol), $Pd(OAc)_2$ (11 mg, 0.05 mmol), sodium diphenylphosphinobenzene-3-sulfonate (TPPMS, 36 mg, 0.1 mmol), and benzyl alcohol **2a** (515 µL, 5 mmol) in D_2O (4 mL) was heated at 120 °C for 16 h in a sealed tube under air. After the reaction mixture was cooled, the organic layer was concentrated in vacuo. The residue was analyzed by ^{1}H-NMR spectroscopy.

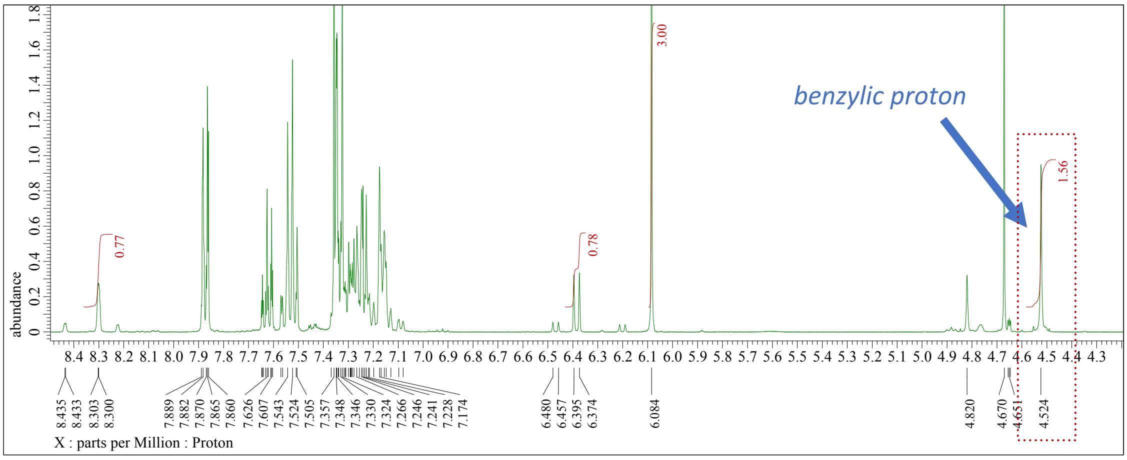

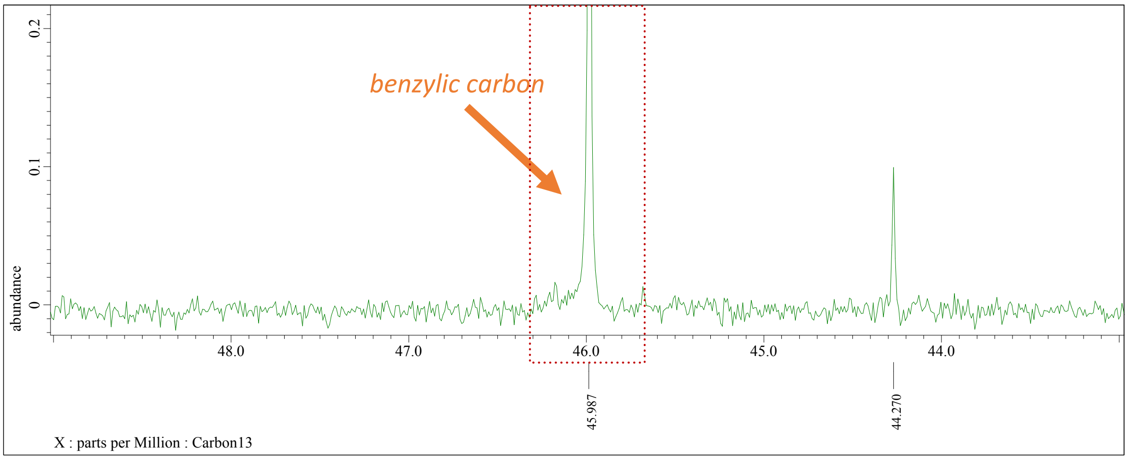

Scheme S9. Scale-up experiment.

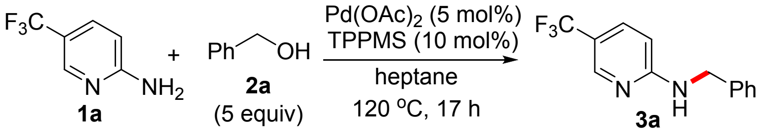

A mixture of 2-amino-5-(trifluoromethyl)pyridine (**1a**) (1.13 g, 7.0 mmol), palladium(II) acetate (78.6 mg, 0.35 mmol), sodium diphenylphosphinobenzene-3-sulfonate (TPPMS, 254.7 mg, 0.7 mmol) and benzyl alcohol (**2a**) (5 mmol) in heptane (28 mL) was heated at 120 °C for 17 h in a sealed tube under air. After cooling, the reaction mixture was poured into water and extracted with EtOAc. The organic layer was washed with brine, dried over $MgSO_4$ and concentrated in vacuo. The residue was triturated with hexane to give desired product **3a** (1.56 g, 6.2 mmol, 88%).

N-Benzyl-5-(trifluoromethyl)pyridin-2-amine 3a

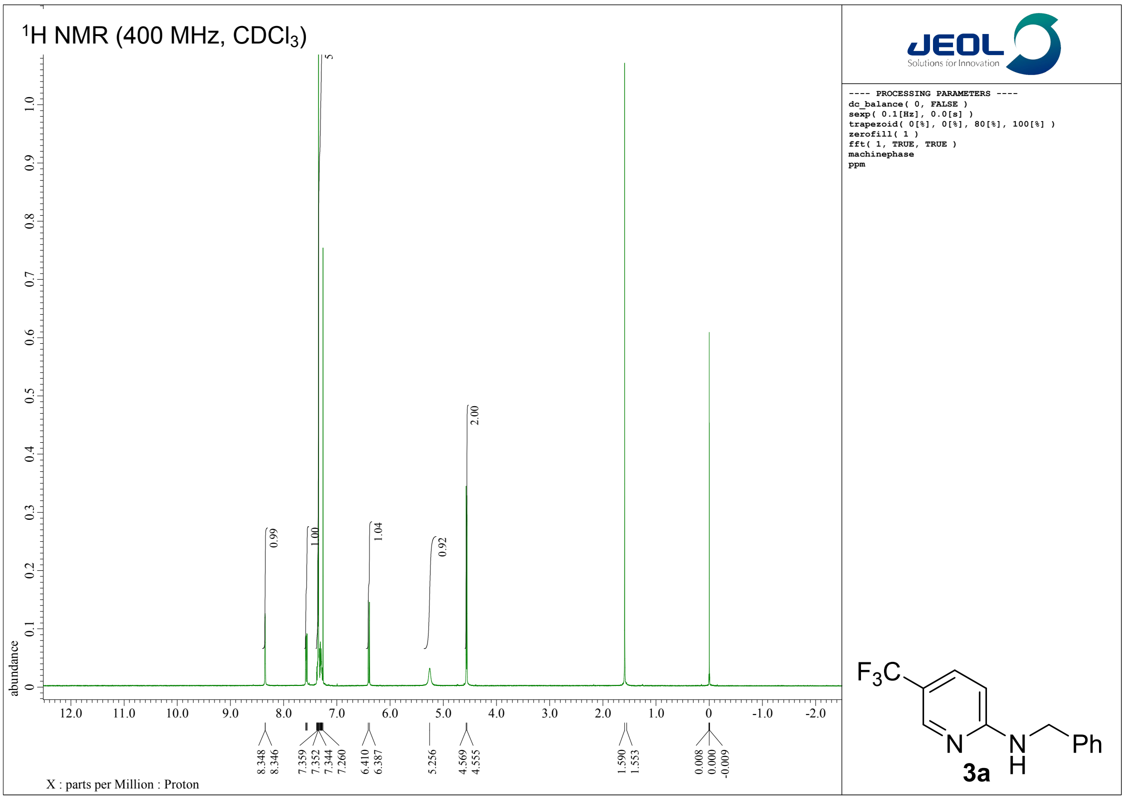

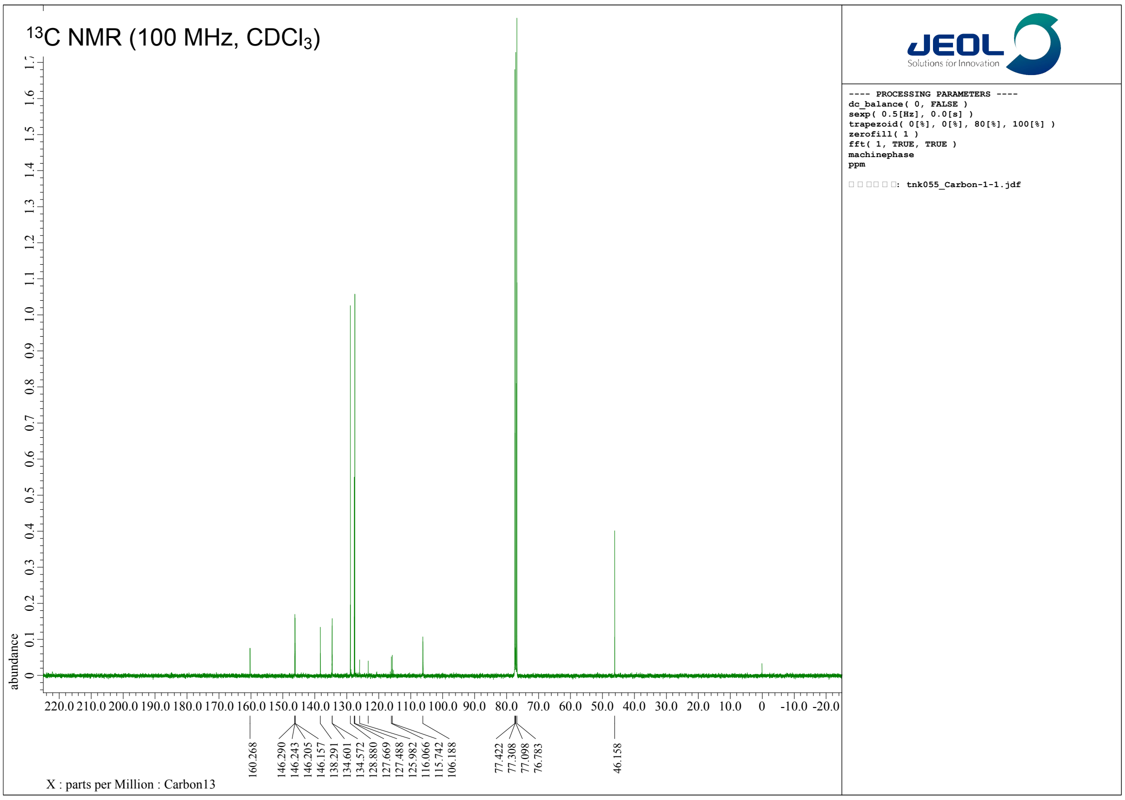

N-Benzyl-5-fluoropyridin-2-amine 3b

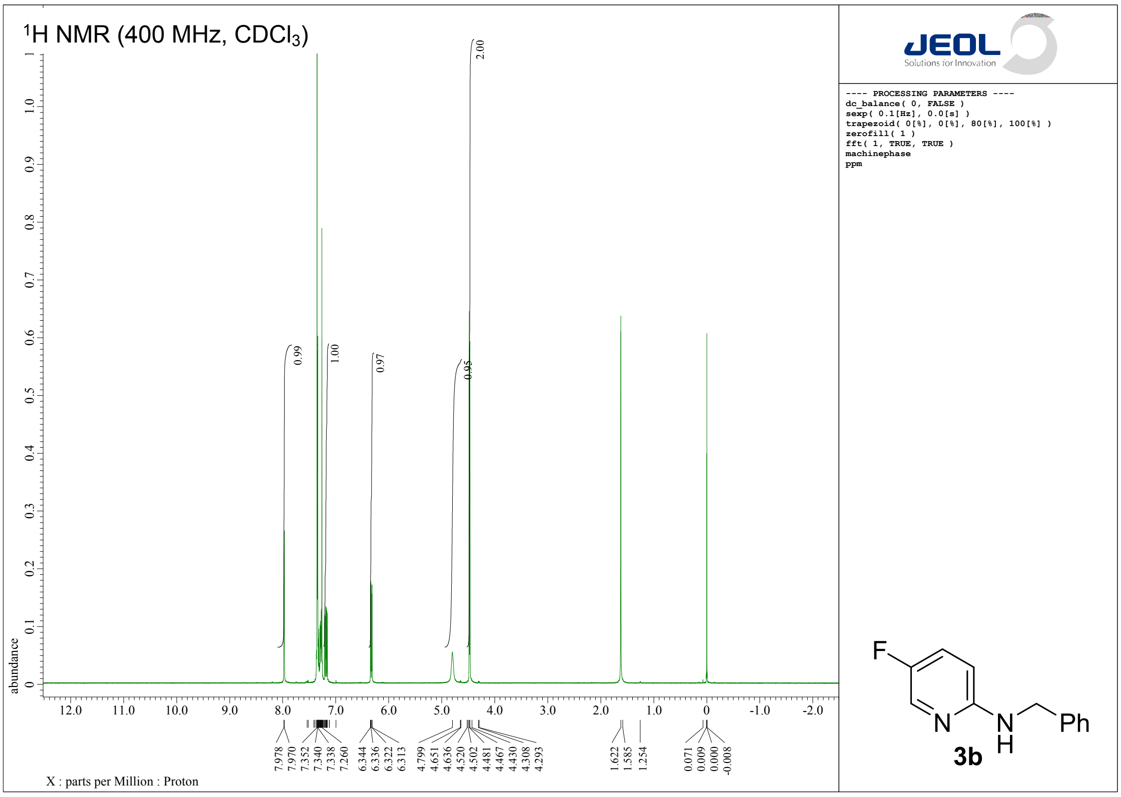

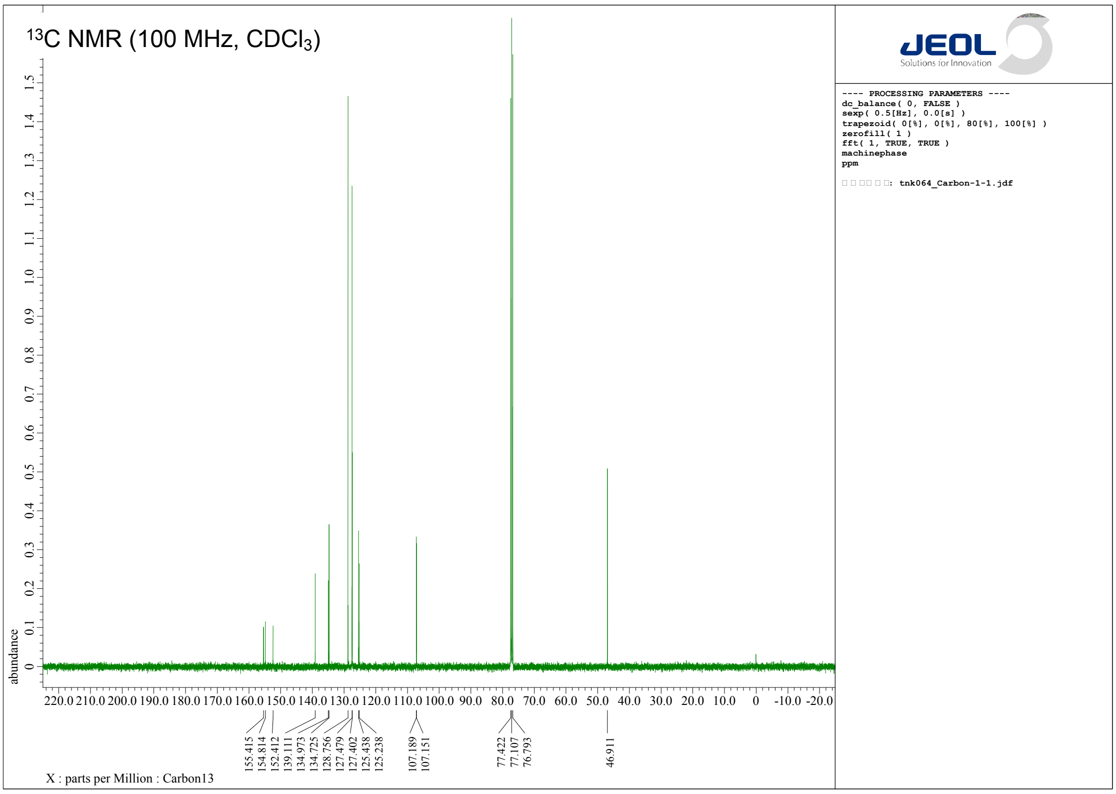

Methyl-6-(benzylamino)nicotinate 3c

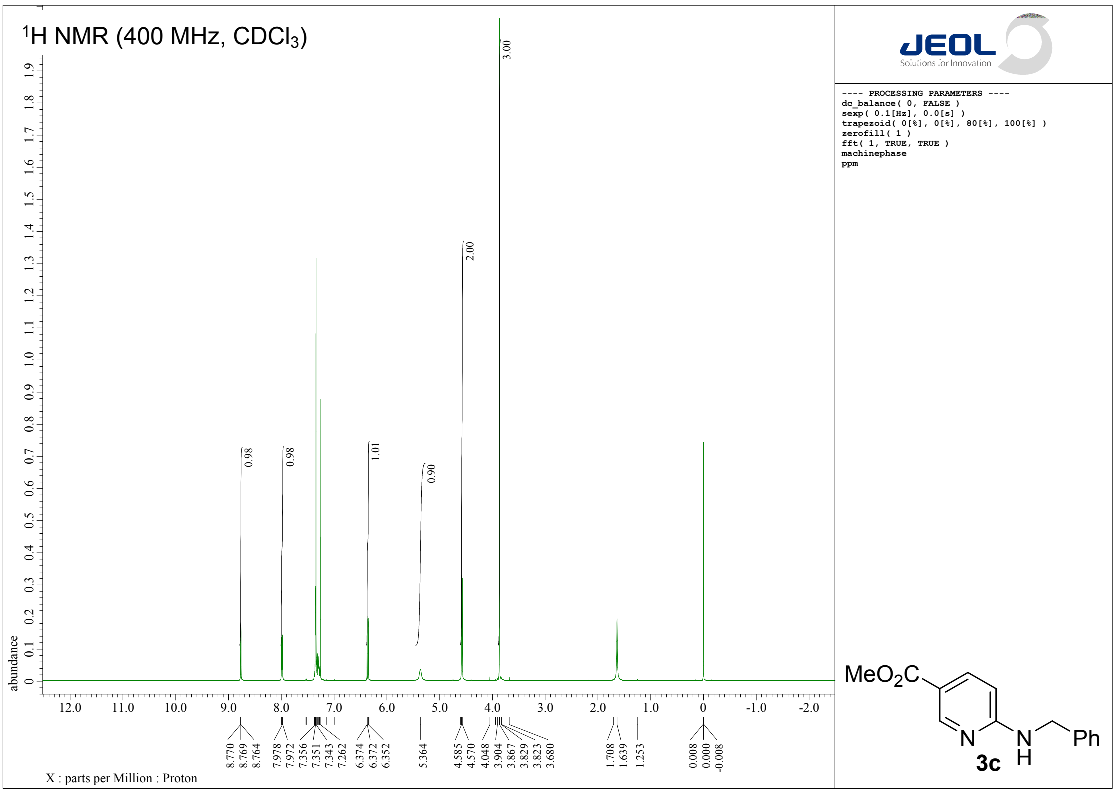

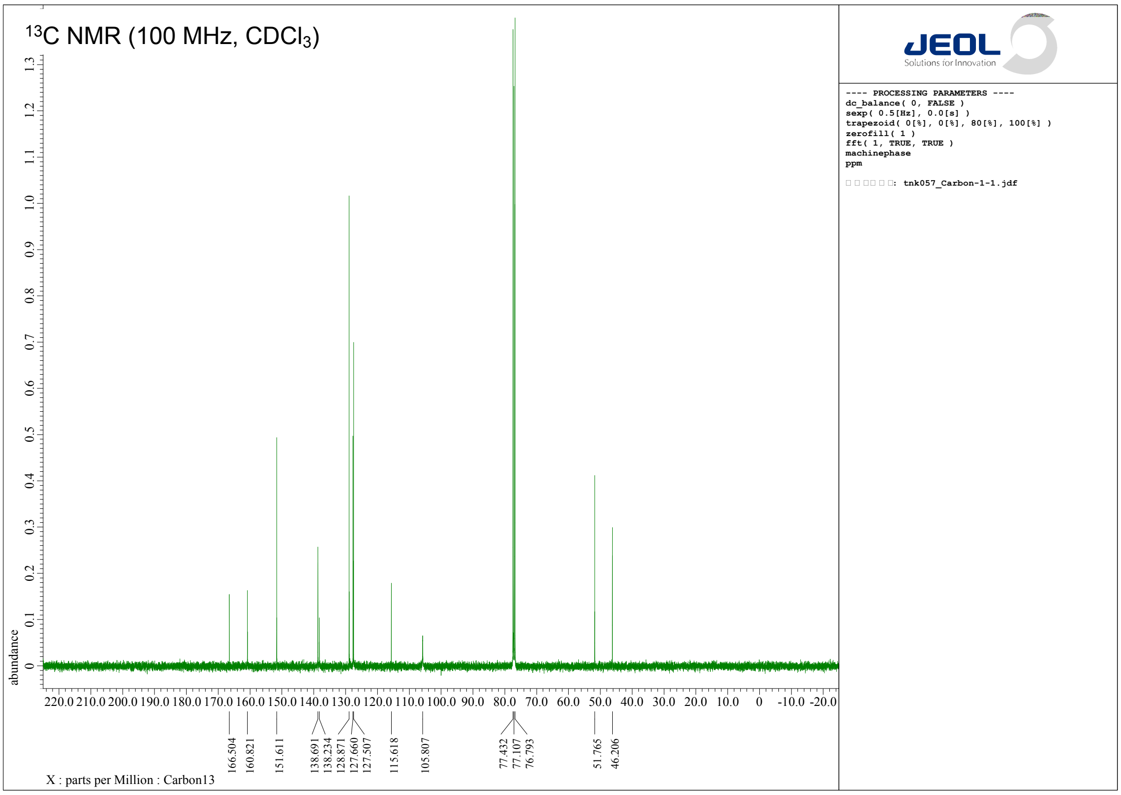

N-Benzyl-5-methylpyridin-2-amine 3d

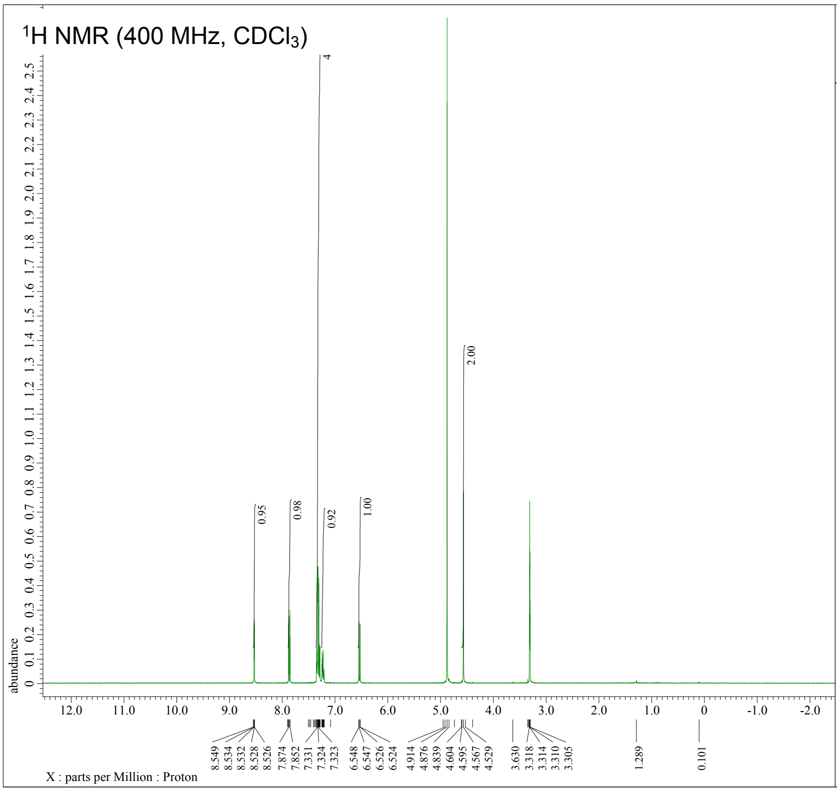


```
---- PROCESSING PARAMETERS ----
dc_balance( 0, FALSE )
sexp( 0.1[Hz], 0.0[s] )
trapezoid( 0[%], 0[%], 80[%], 100[%] )
zerofill( 1 )
fft( 1, TRUE, TRUE )
machinephase
ppm
```

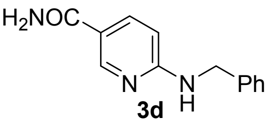

3d

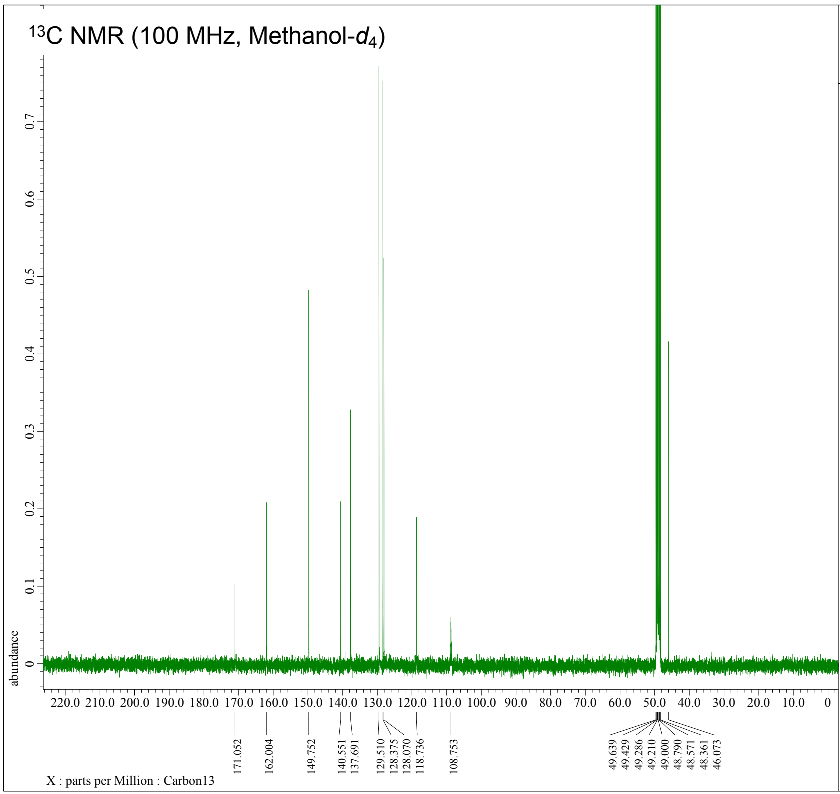


```
Filename          = tnk065_Carbon-1-2.jdf
Author            = delta
Experiment        = carbon.jxp
Sample_Id         = tnk065
Solvent           = METHANOL-D4
Actual_Start_Time = 26-JUN-2018 22:15:30
Revision_Time     =  8-DEC-2020 09:59:54

Comment           = single pulse decoupled ga
Data_Format       = 1D COMPLEX
Dim_Size          = 26214
X_Domain          = Carbon
Dim_Title         = Carbon13
Dim_Units         = [ppm]
Dimensions        = X
Site              = JNM-ECS400
Spectrometer      = DELTA2_NMR

Field_Strength    = 9.389766[T] (400[MHz])
X_Acq_Duration    = 1.04333312[s]
X_Domain          = 13C
X_Freq            = 100.52530333[MHz]
X_Offset          = 100[ppm]
X_Points          = 32768
X_Prescans        = 4
X_Resolution      = 0.95846665[Hz]
X_Sweep           = 31.40703518[kHz]
X_Sweep_Clipped   = 25.12562814[kHz]
Irr_Domain        = Proton
Irr_Freq          = 399.78219838[MHz]
Irr_Offset        = 5[ppm]
Clipped           = FALSE
Scans             = 1024
Total_Scans       = 1024

Relaxation_Delay  = 2[s]
Recvr_Gain        = 60
Temp_Get          = 22.4[dC]
X_90_Width        = 9.36[us]
X_Acq_Time        = 1.04333312[s]
X_Angle           = 30[deg]
X_Atn             = 3.3[dB]
X_Pulse           = 3.12[us]
Irr_Atn_Dec       = 20.394[dB]
Irr_Atn_Noe       = 20.394[dB]
Irr_Noise         = WALTZ
Irr_Pwidth        = 0.115[ms]
Decoupling        = TRUE
Initial_Wait      = 1[s]
Noe               = TRUE
Noe_Time          = 2[s]
Repetition_Time   = 3.04333312[s]
```

6-(Benzylamino)nicotinamide 3e

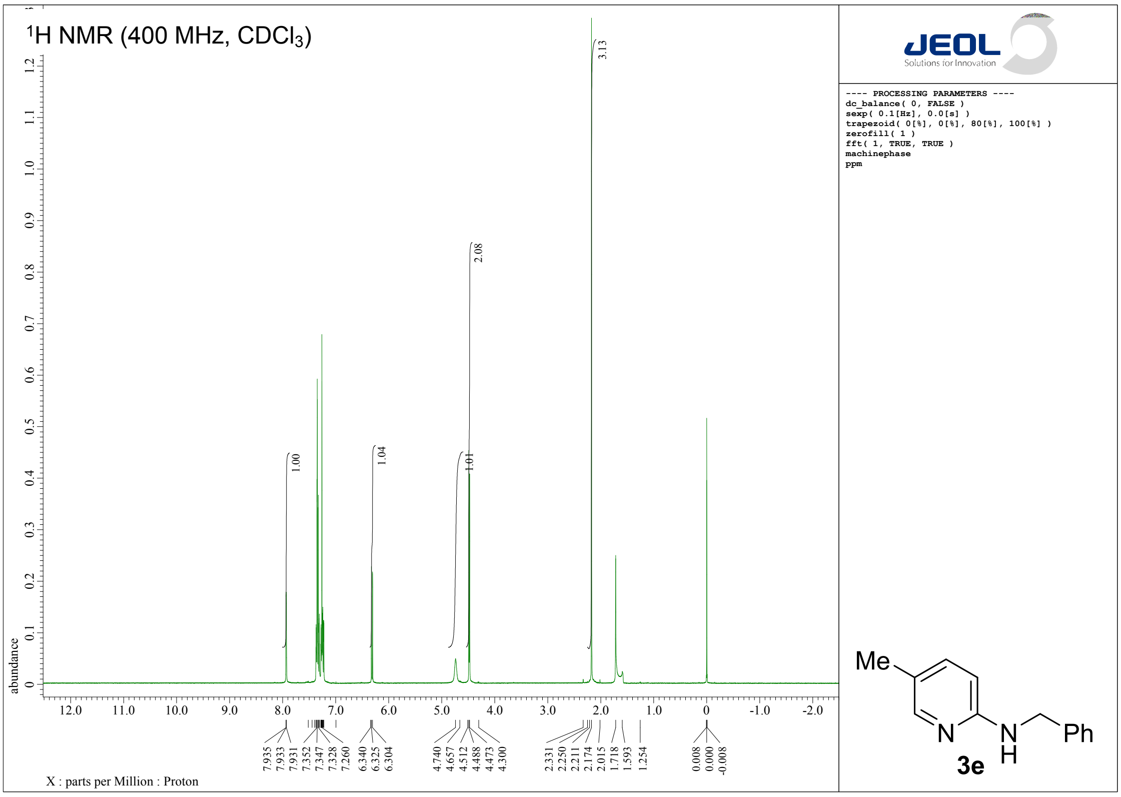

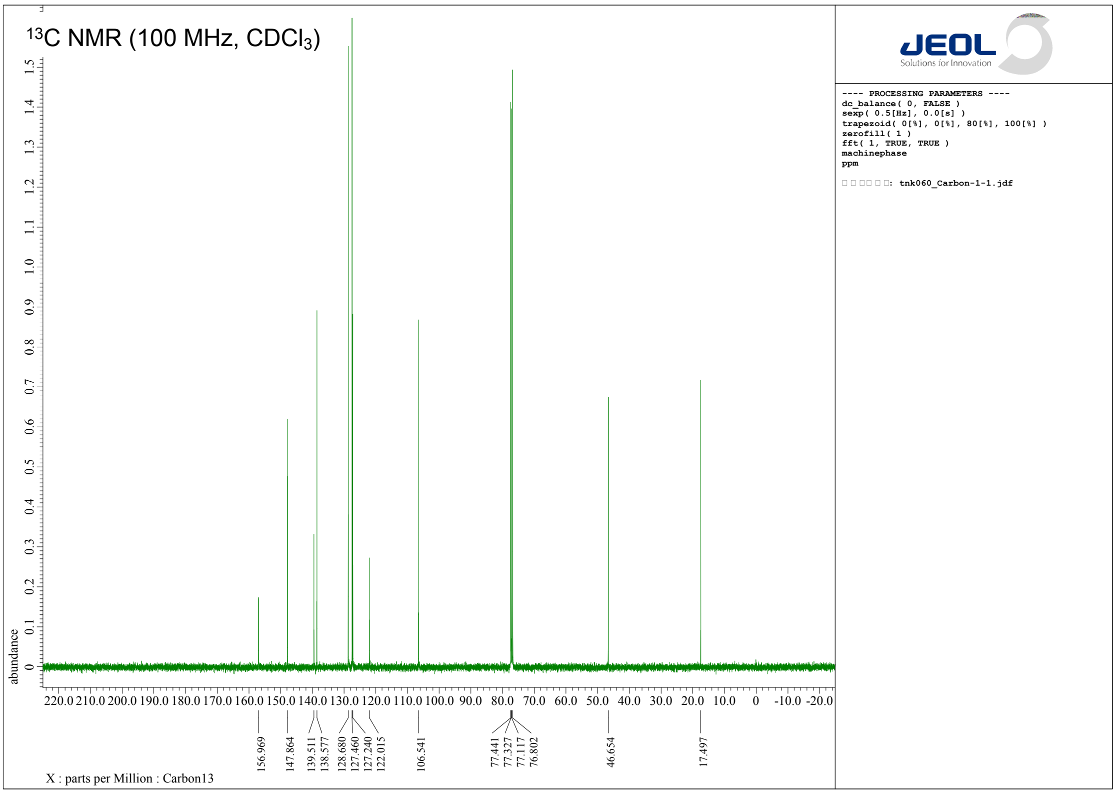

N-Benzylpyridin-2-amine 3f

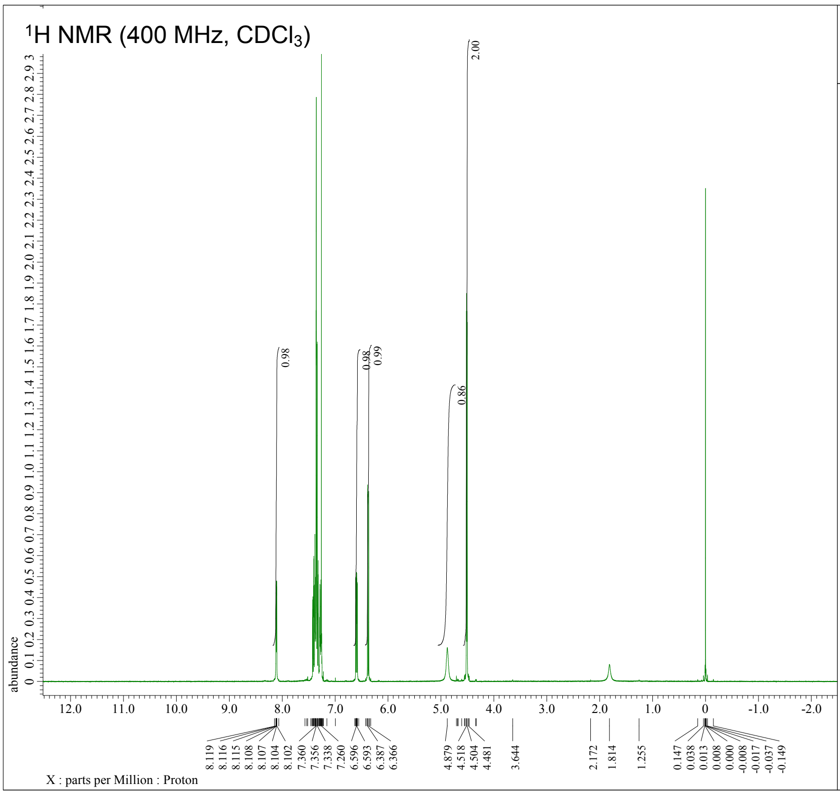

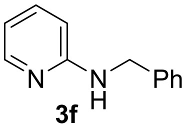

3f

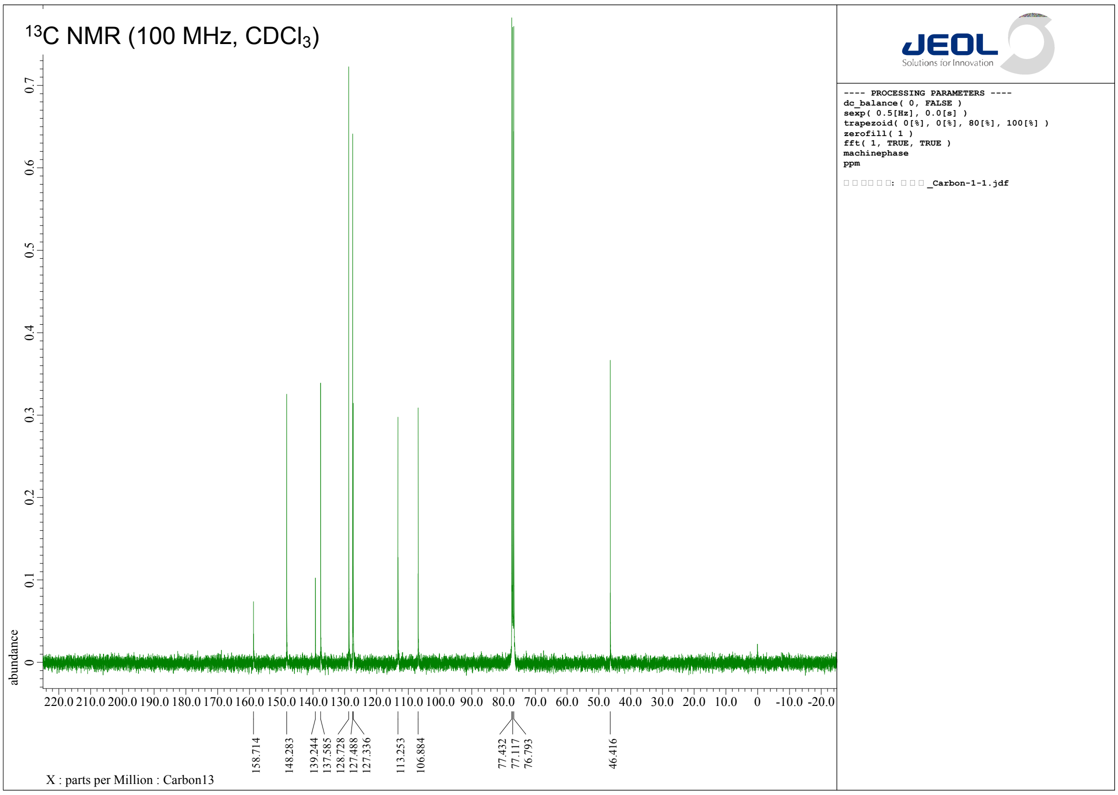

_N_-Benzyl-5-nitropyridin-2-amine 3g

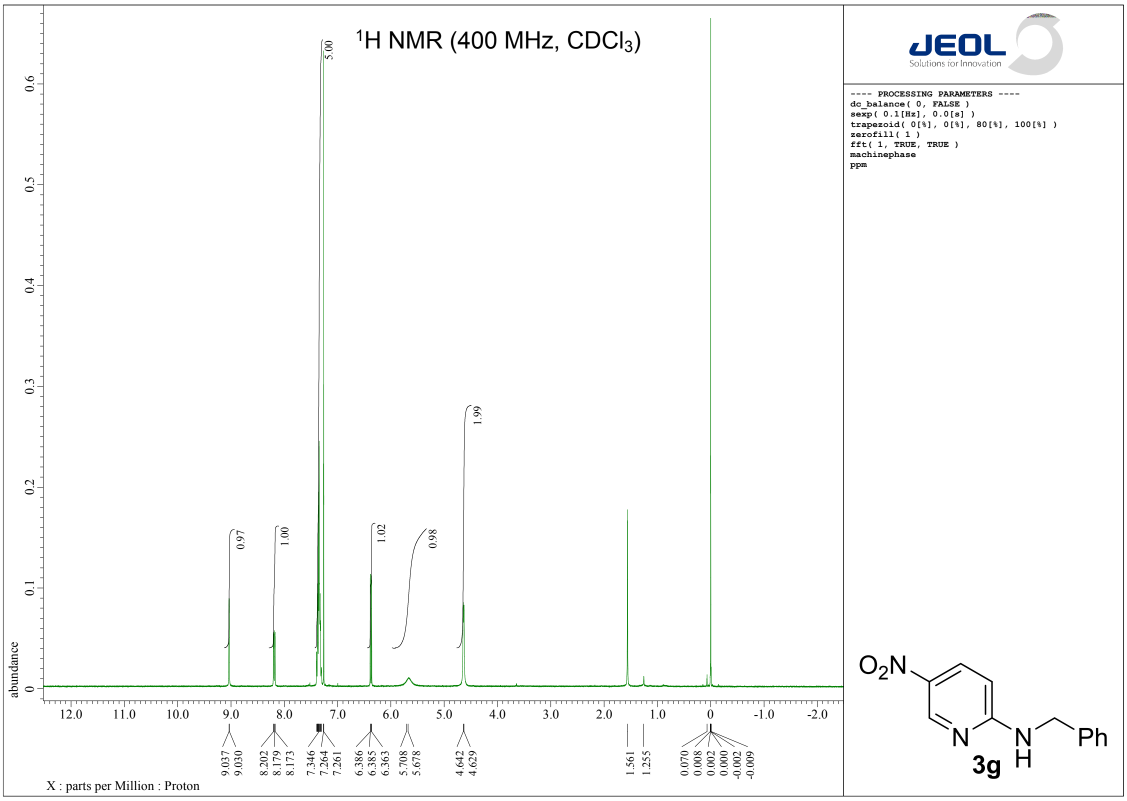

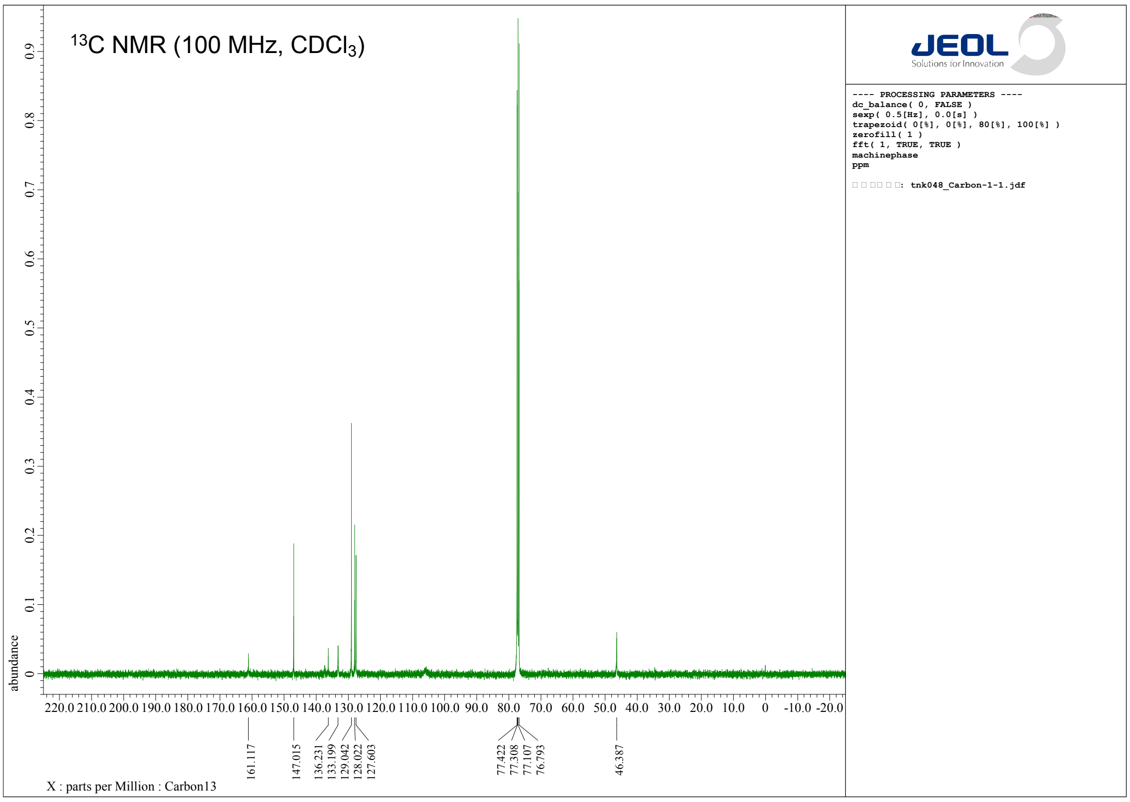

6-Benzylaminonicotinicacid 3h

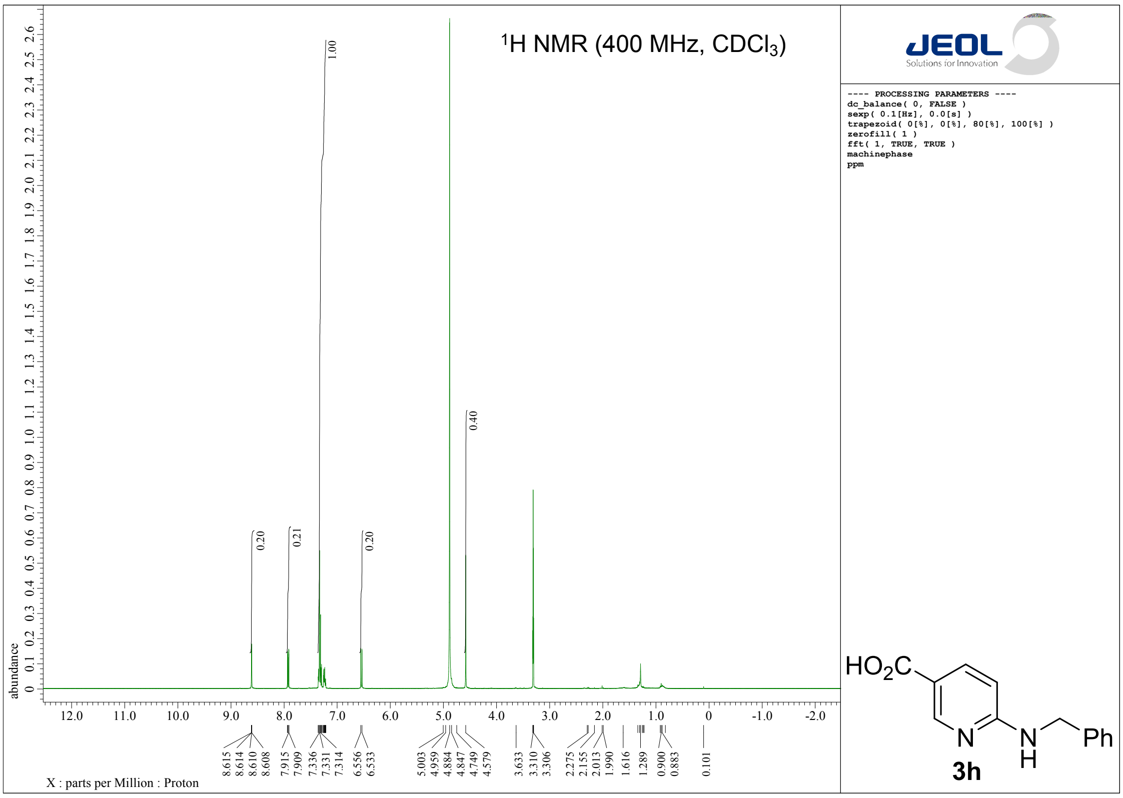

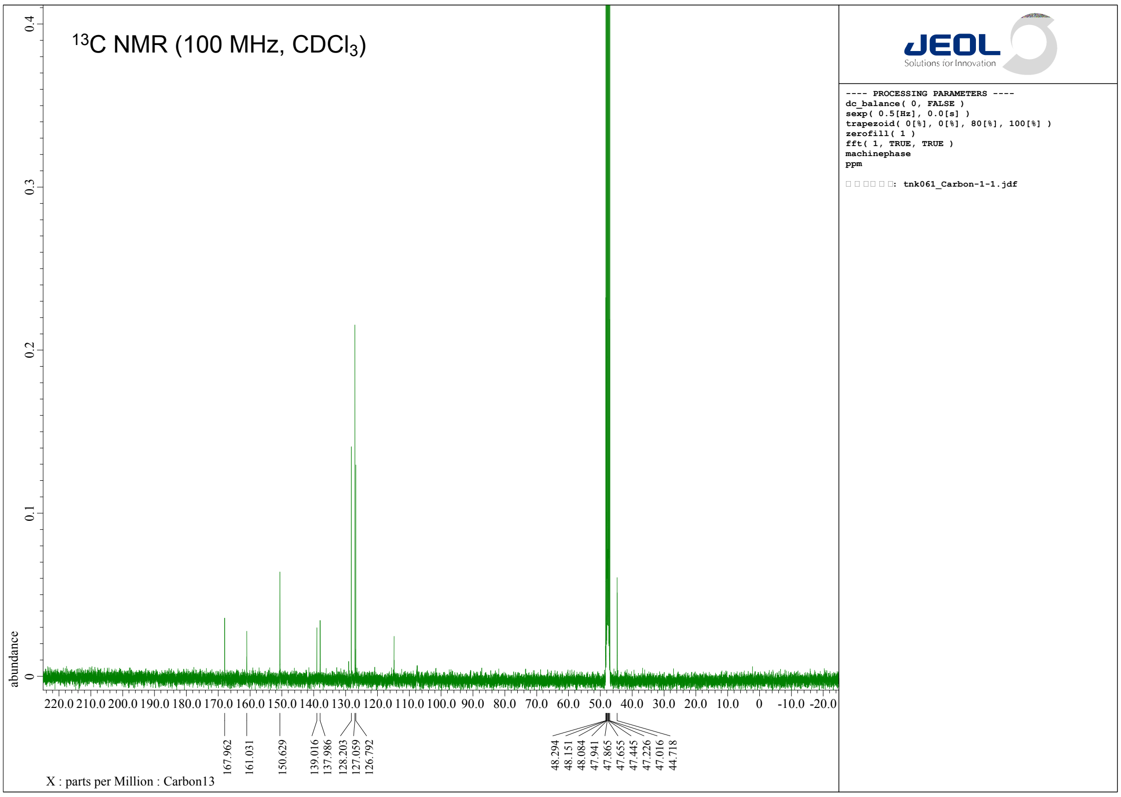

N-Benzylpyridin-3-amine 3i

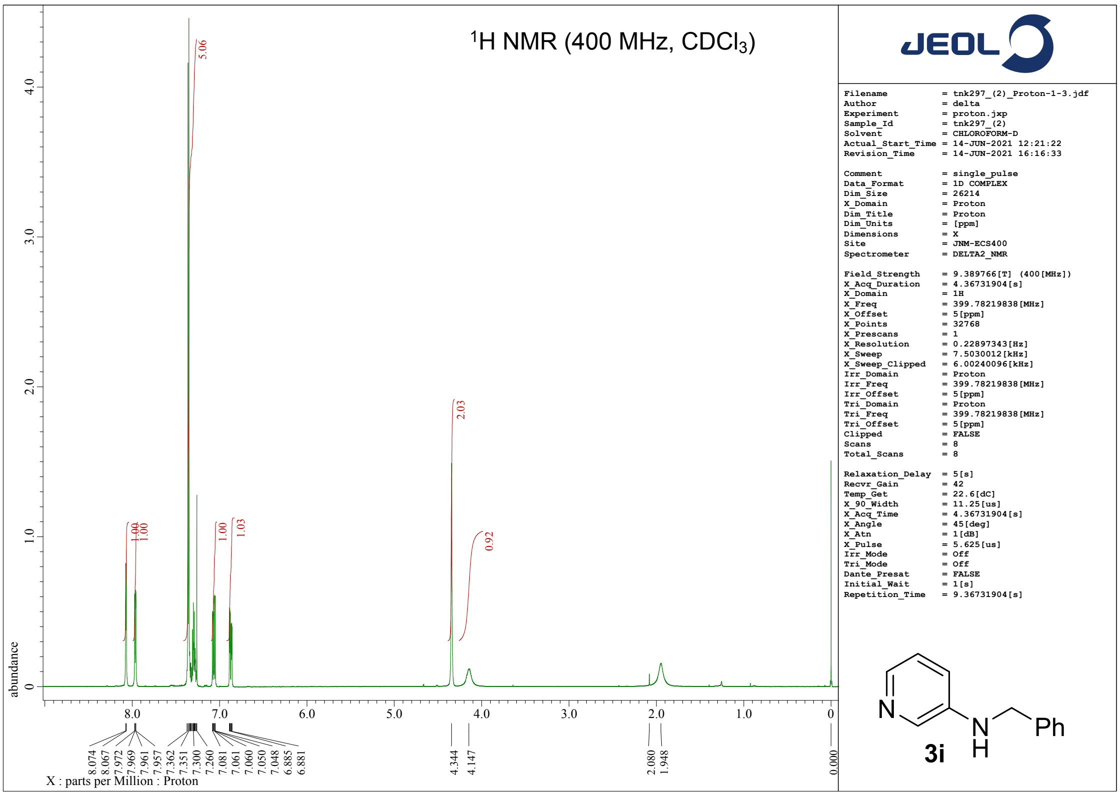

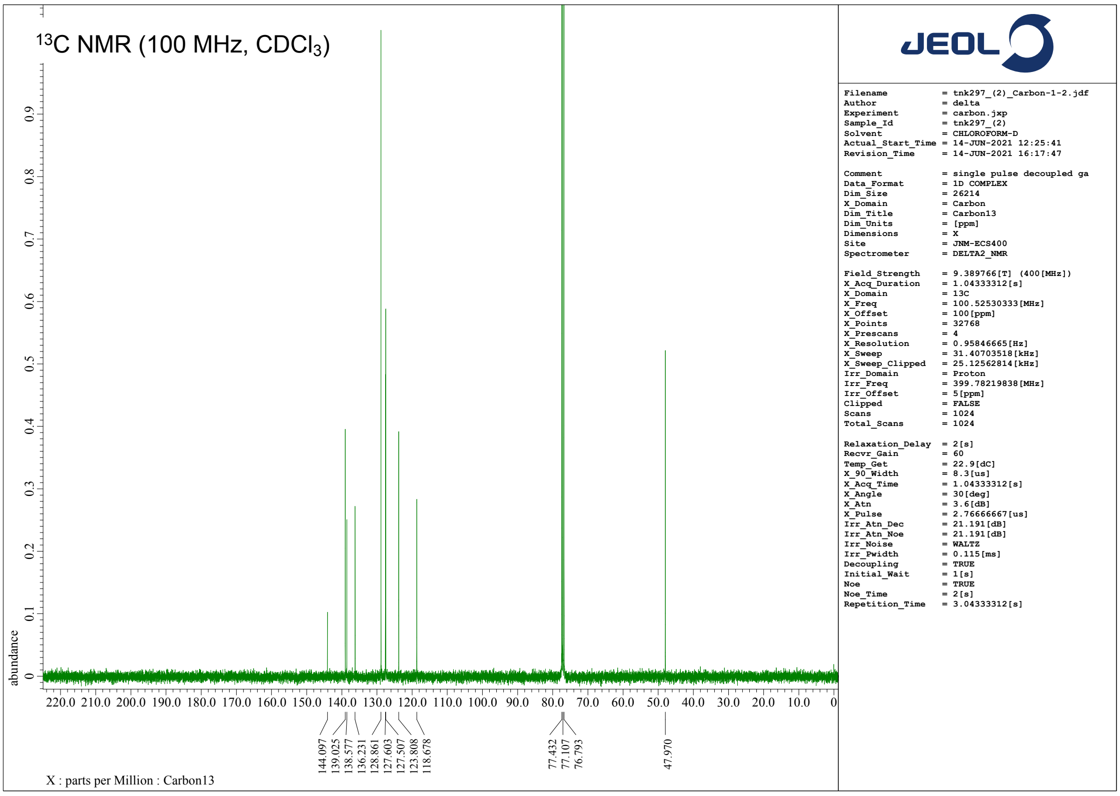

N-Benzylquinolin-2-amine 3j

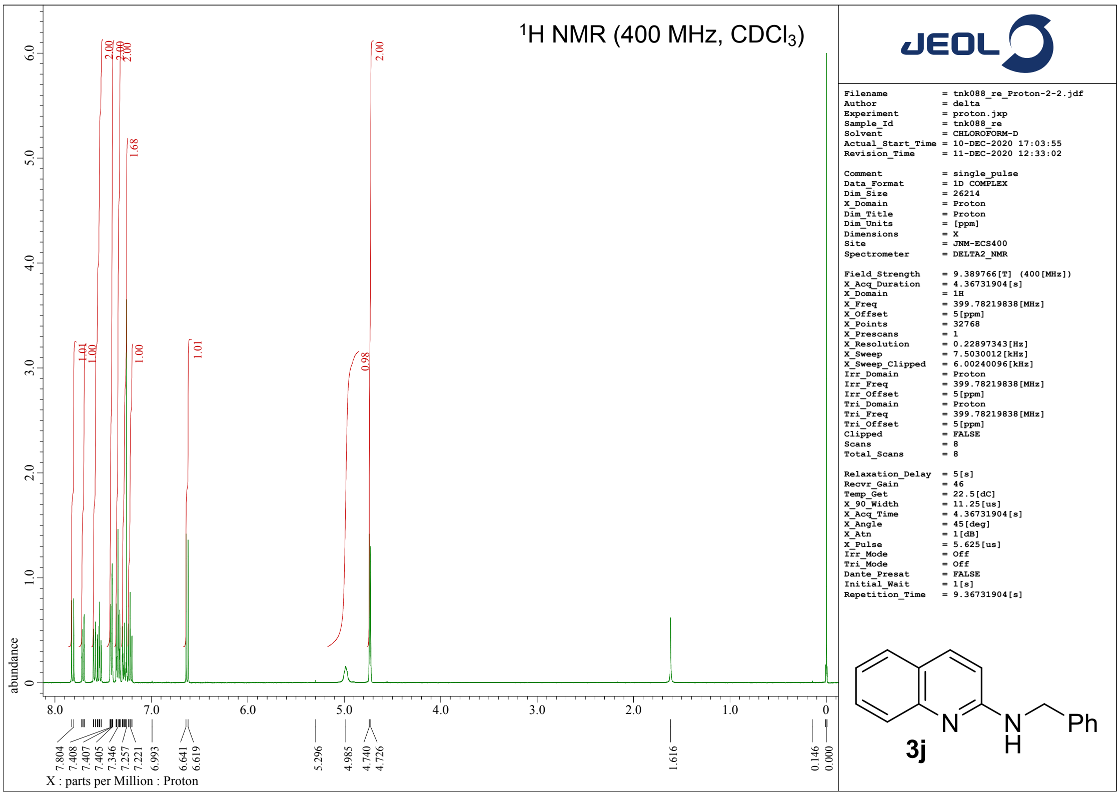

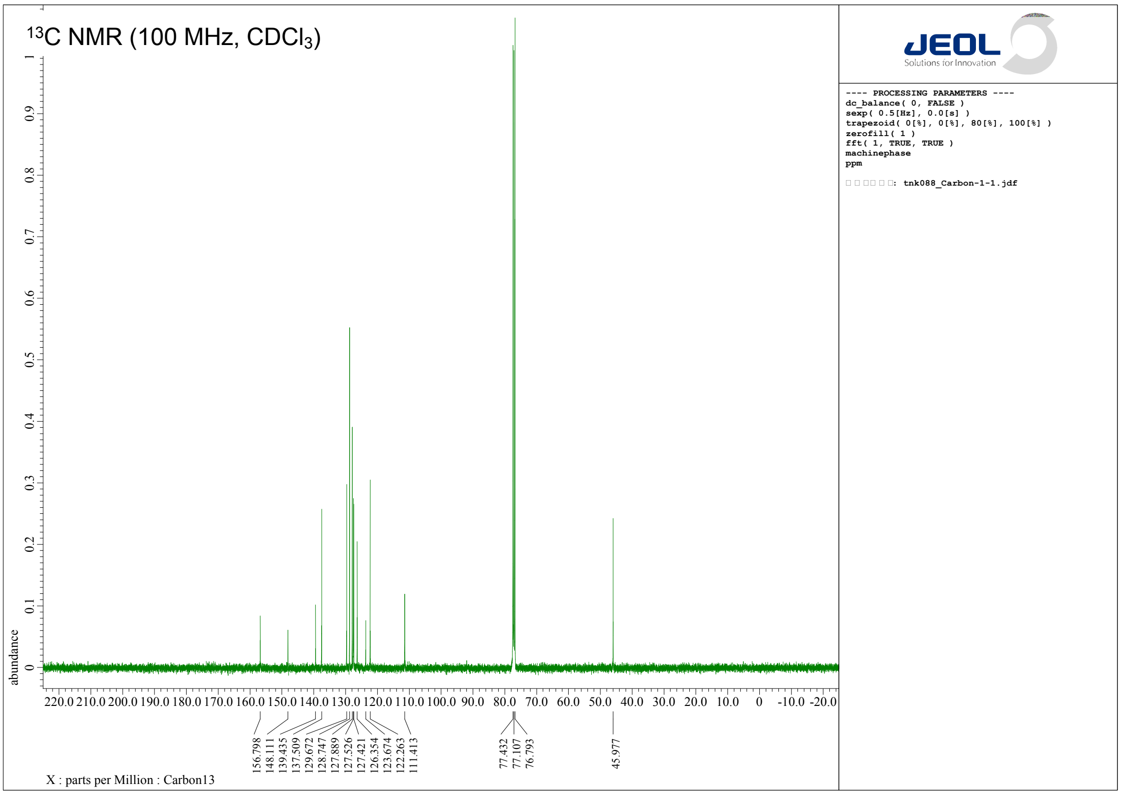

N-Benzylnicotinamide 3k

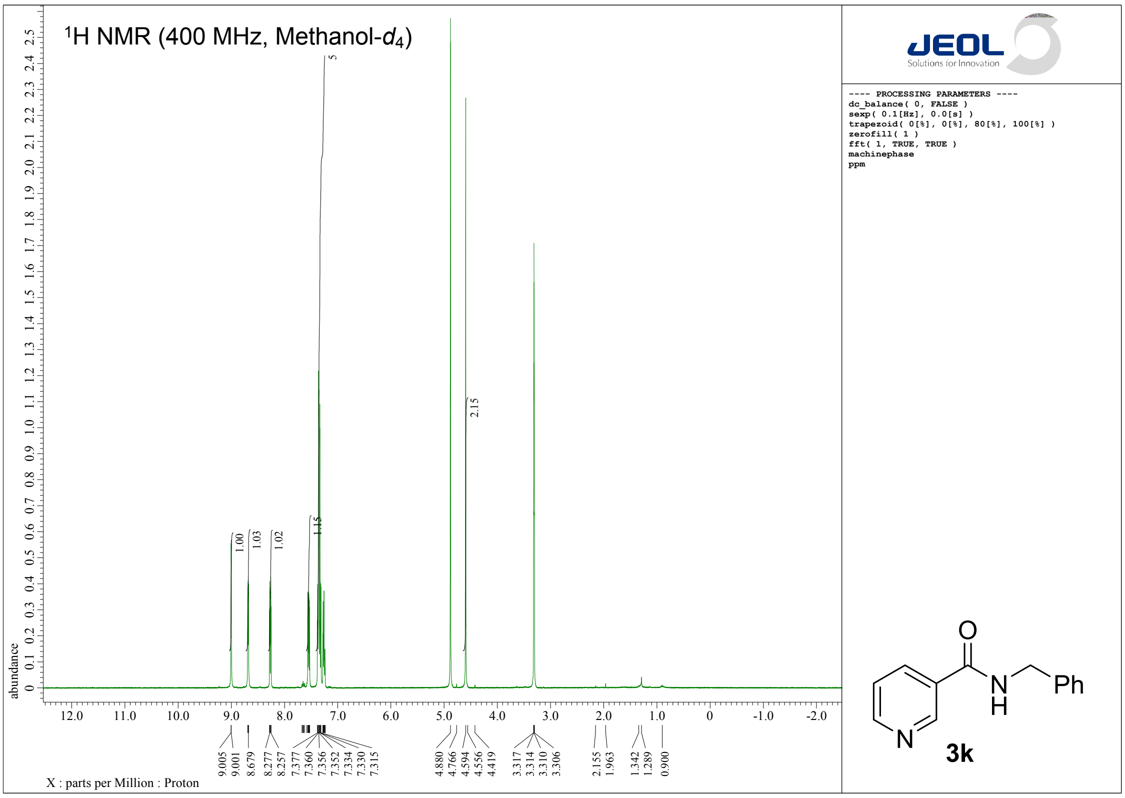

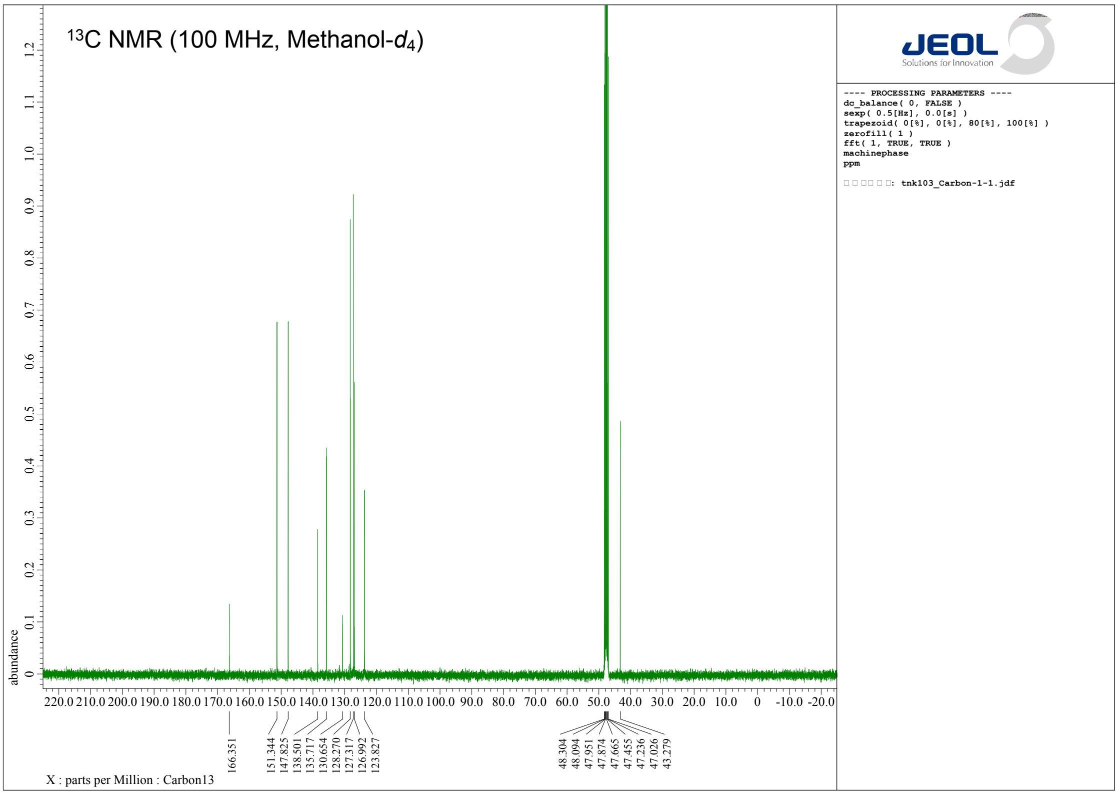

N-(4-Methoxybenzyl)-5-(trifluoromethyl)pyridin-2-amine 3l

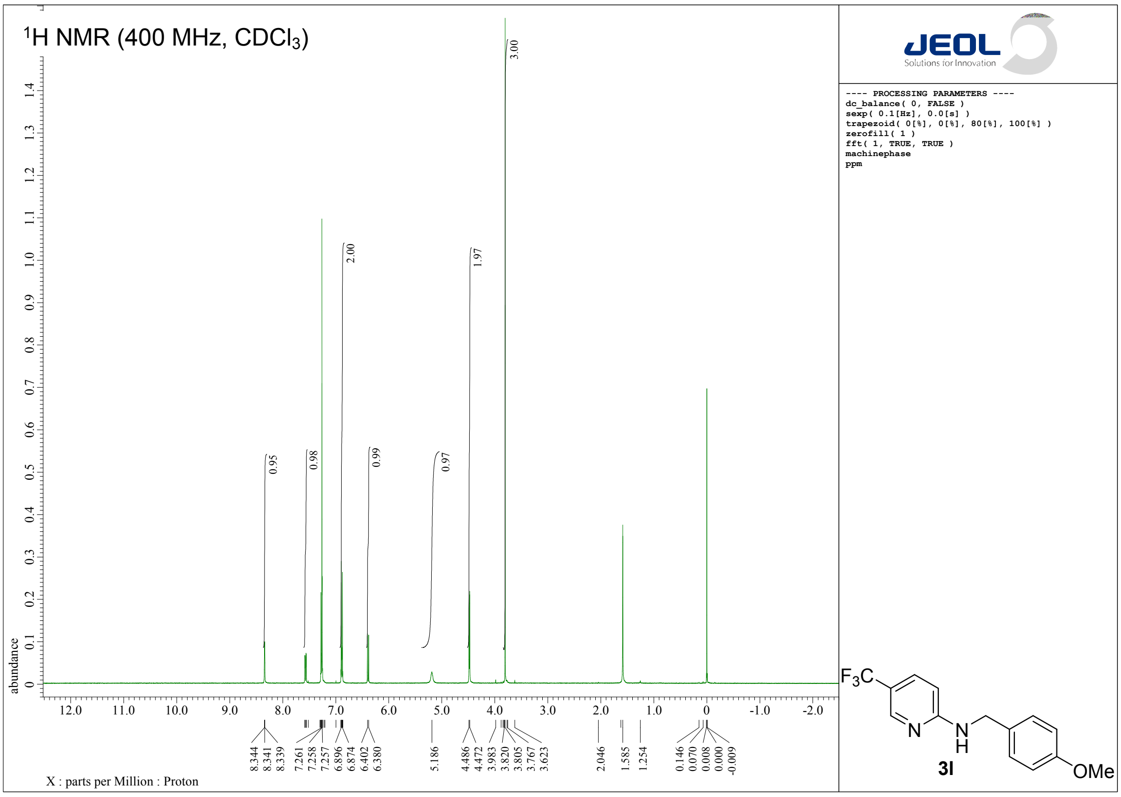

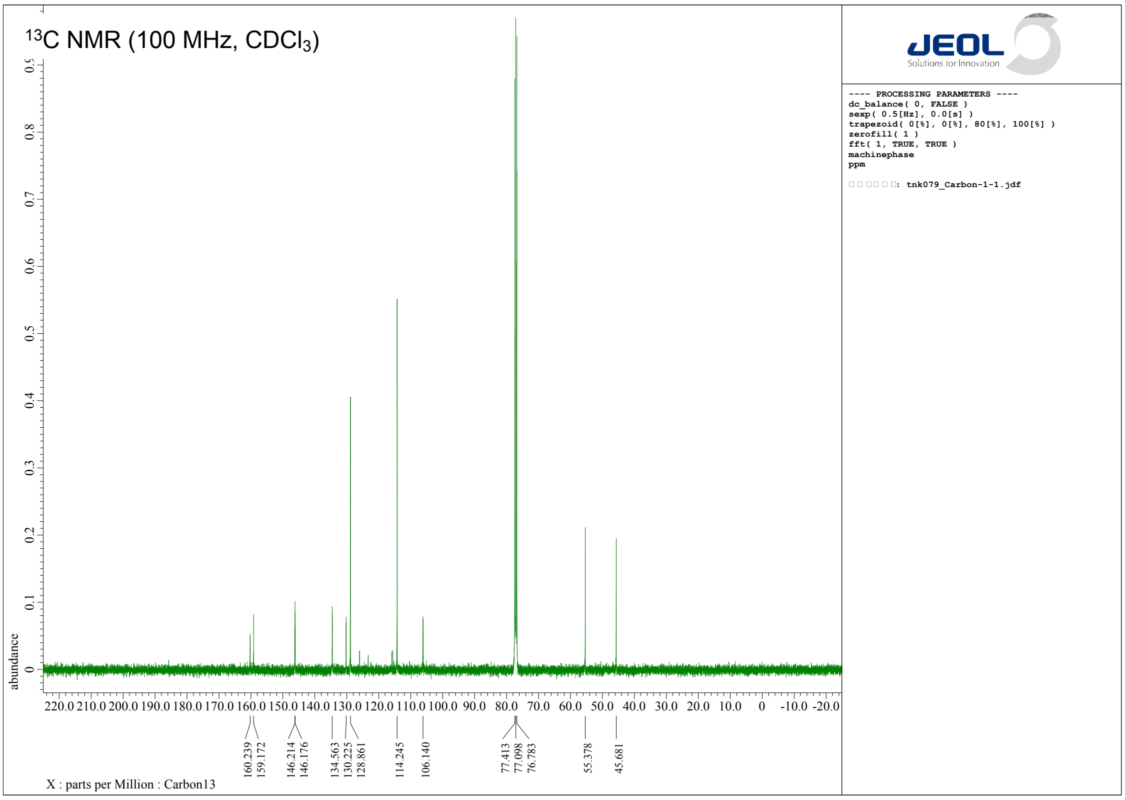

Methyl-6-(4-methoxybenzylamino)nicotinate 3m

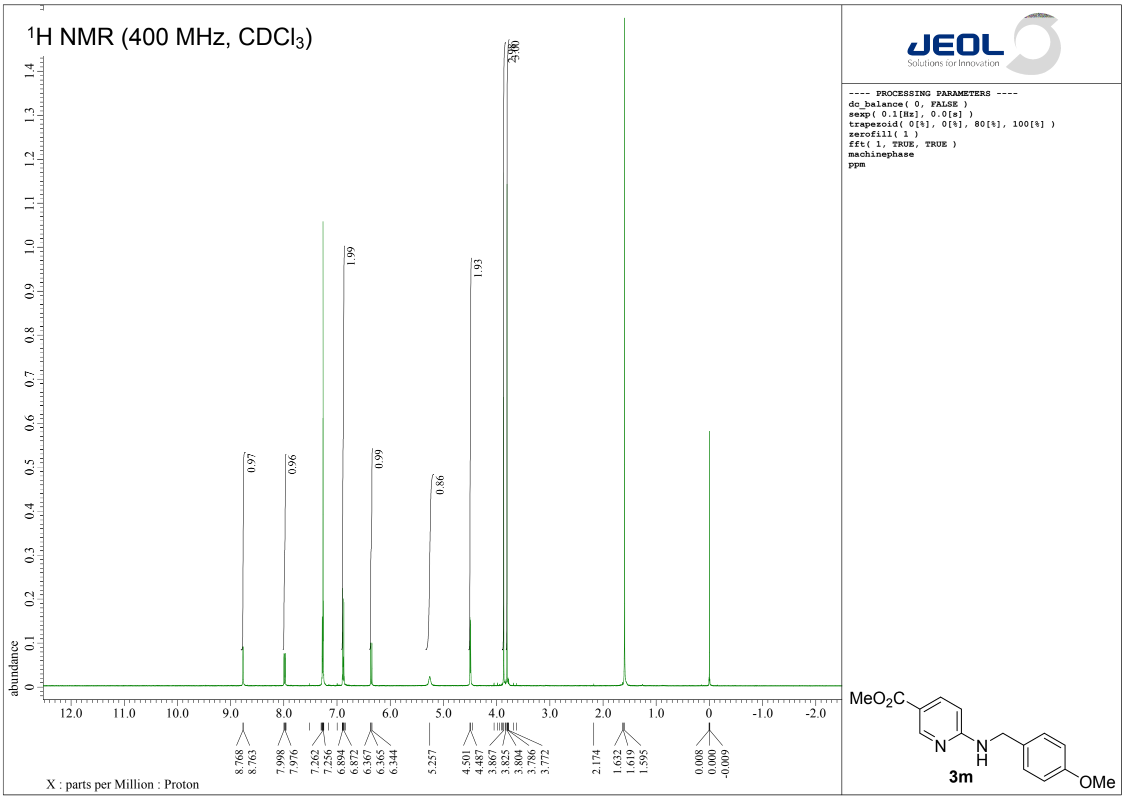

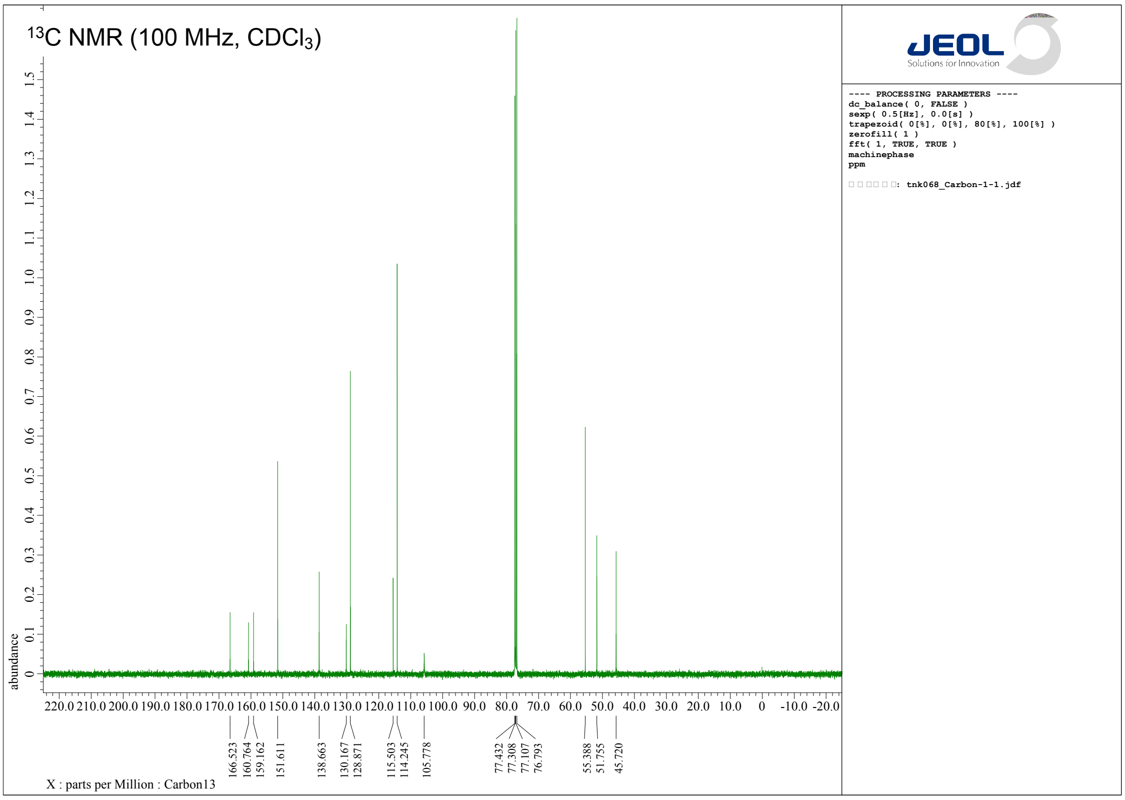

N-(4-Methoxybenzyl)pyridin-2-amine 3n

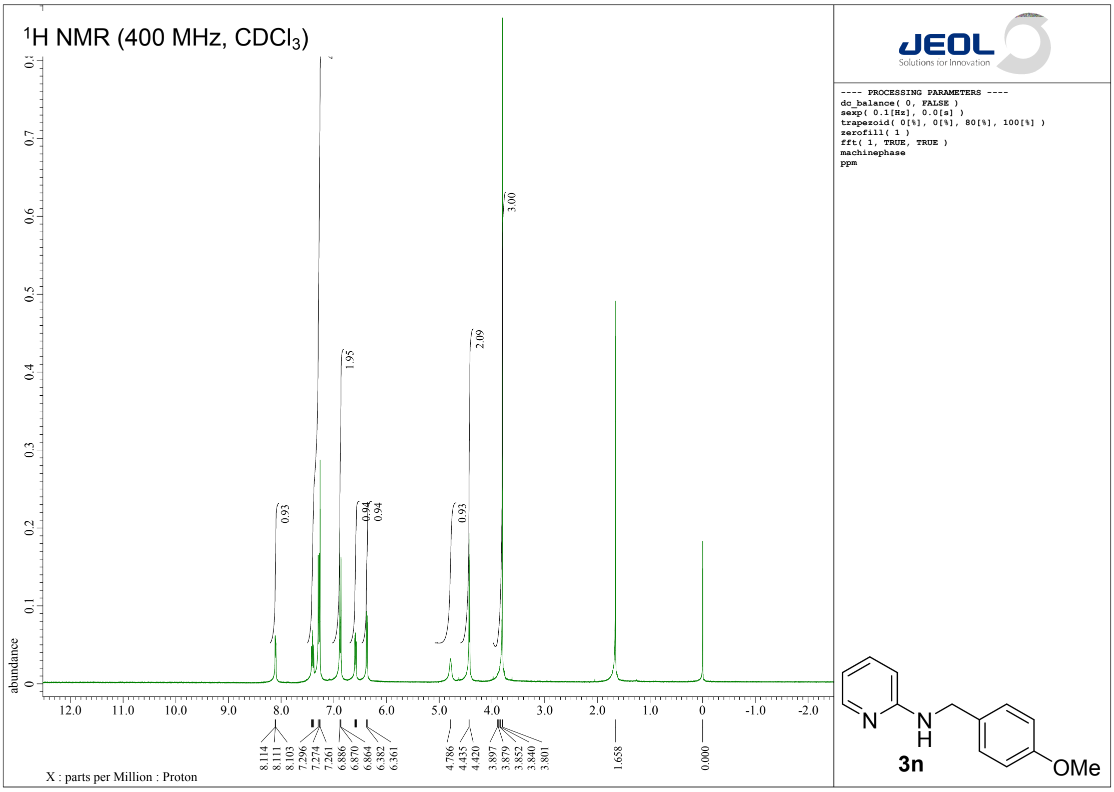

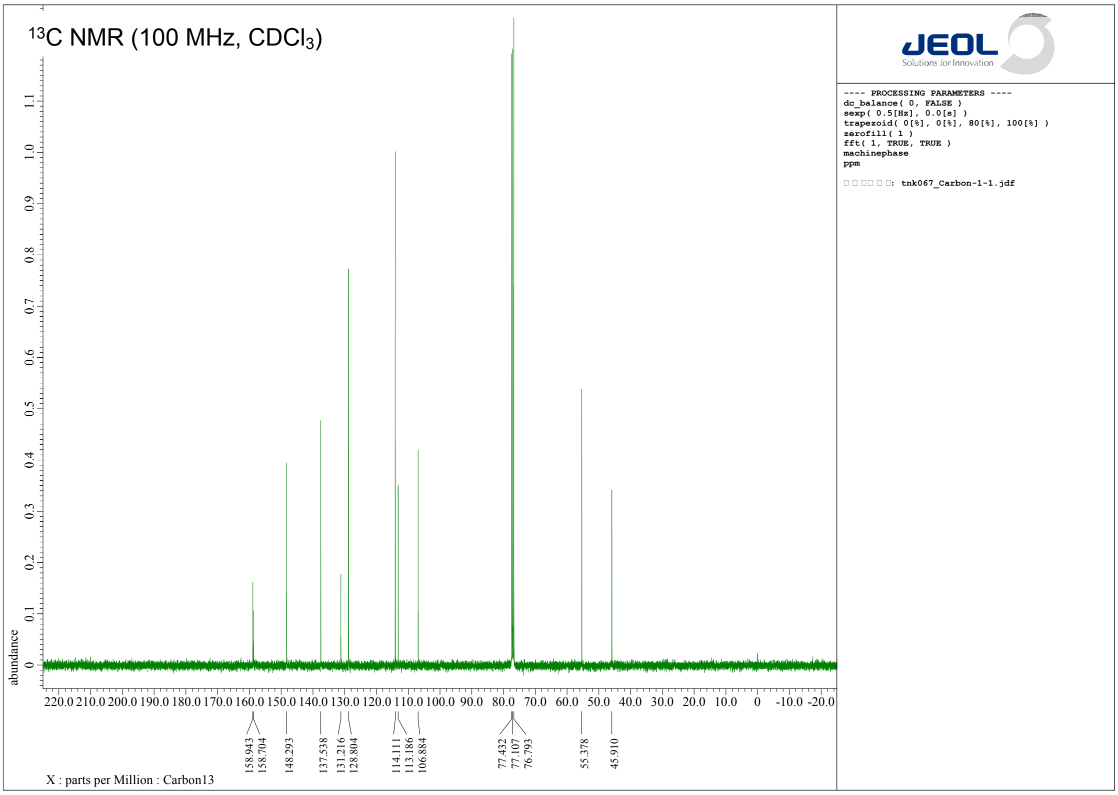

N-(4-Methoxybezyl)-5-methylpyridin-2-amine 3o

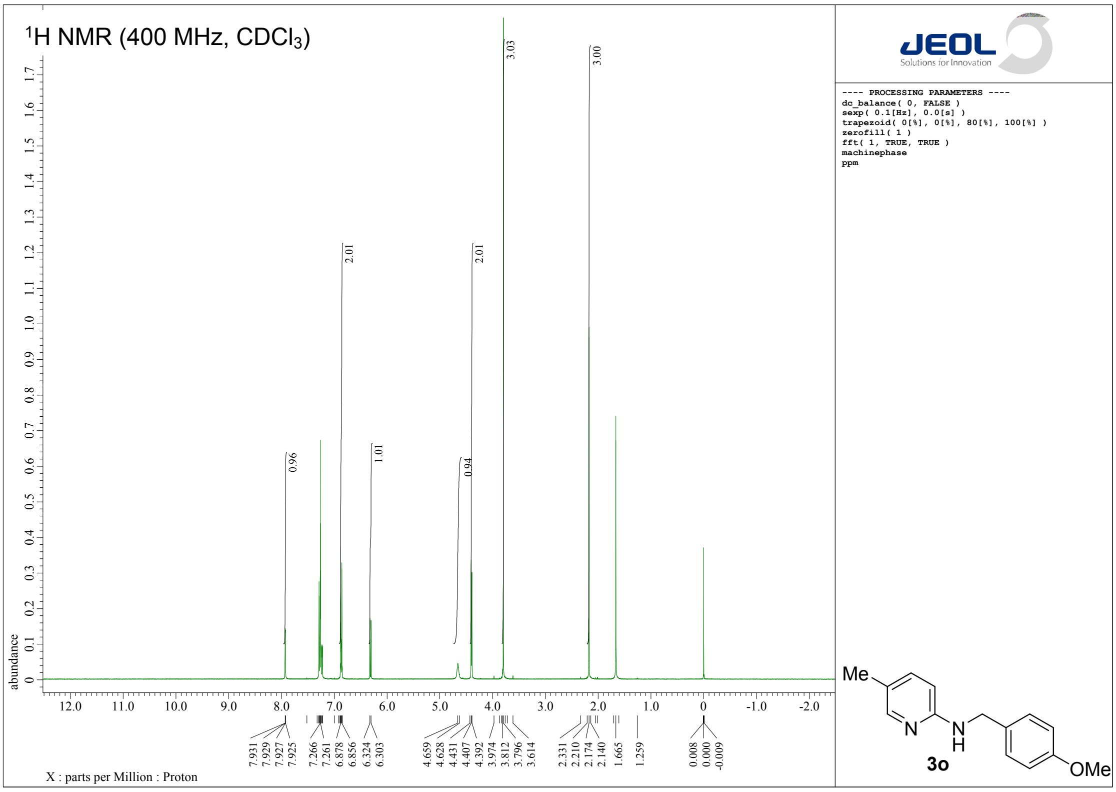

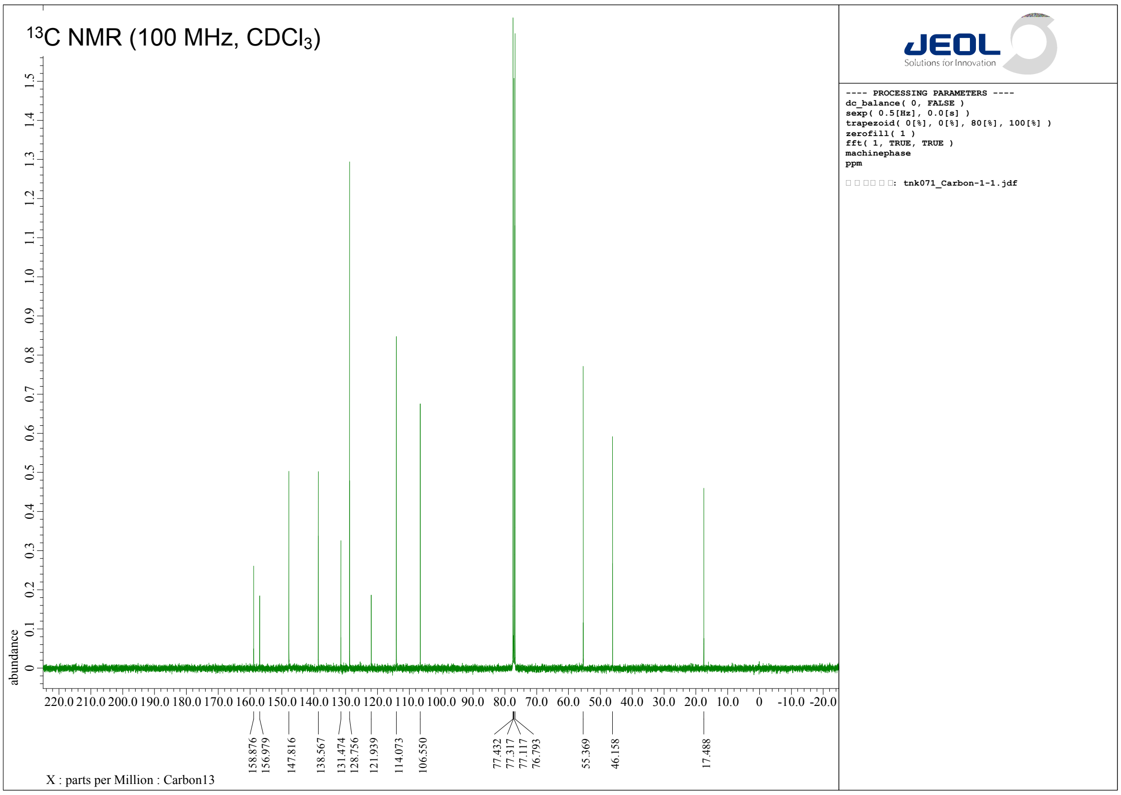

6-(4-Methoxybenzylamino)nicotinamide 3p

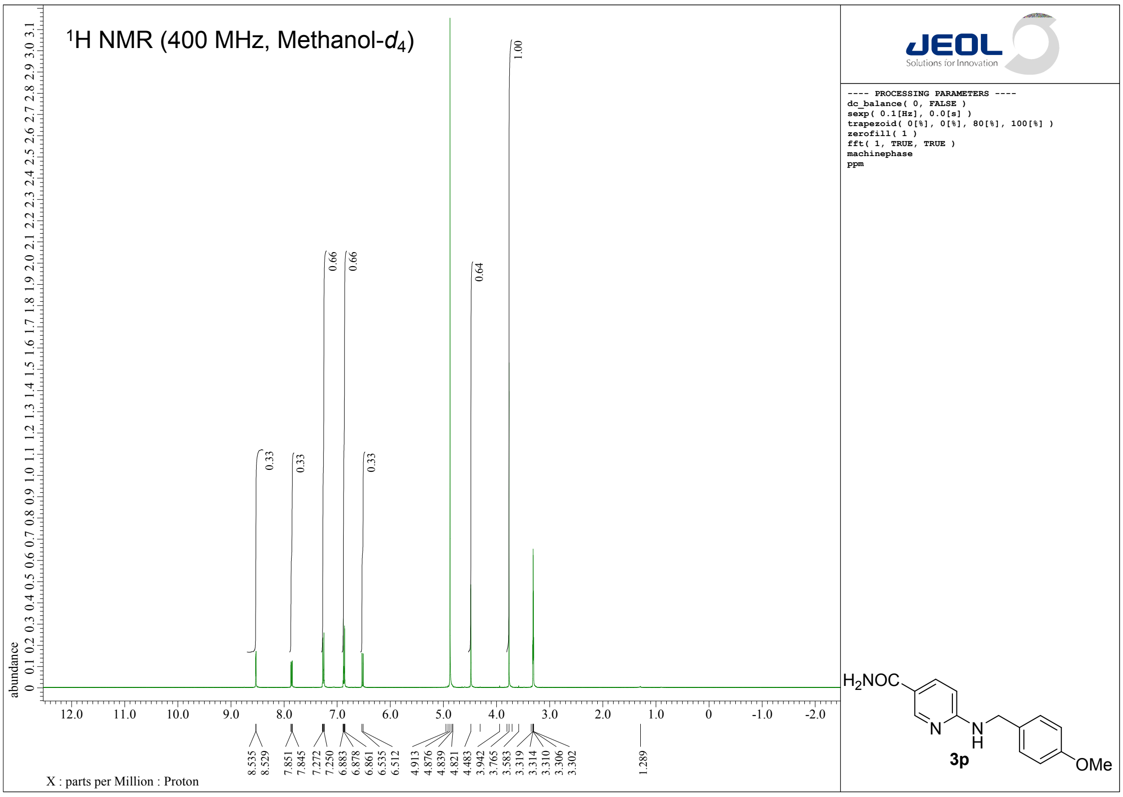

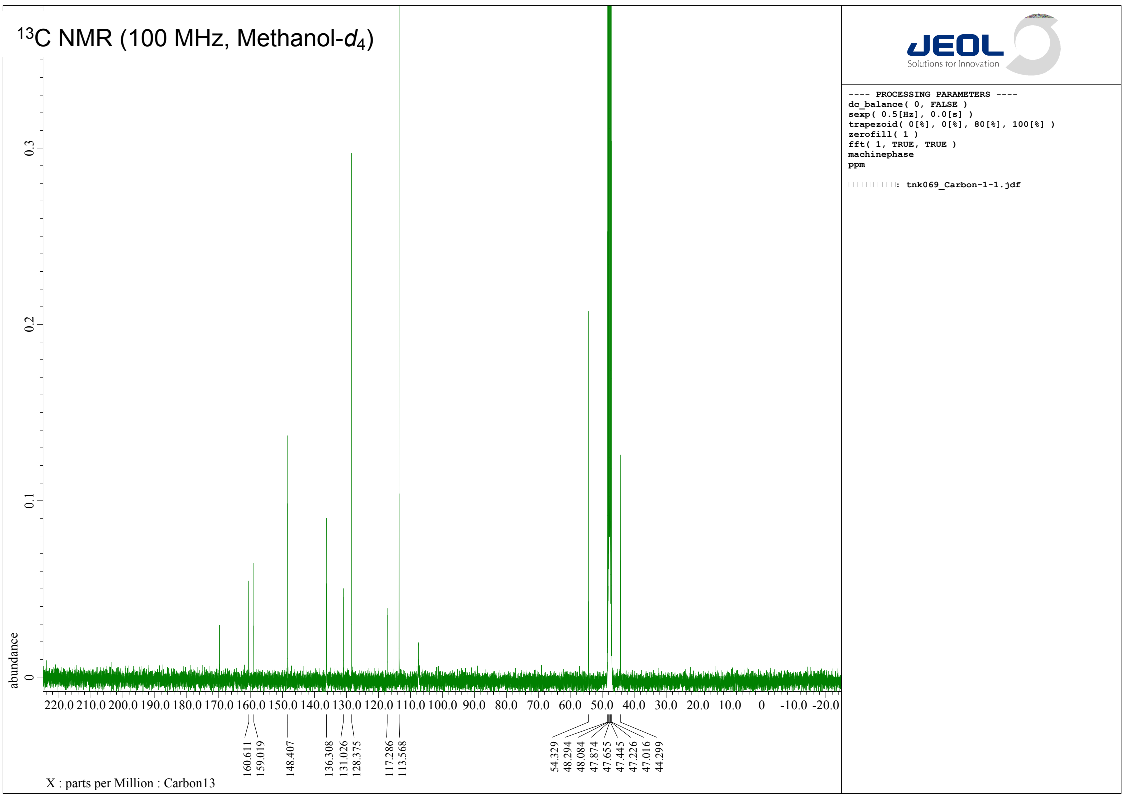

N-(4-Methylbenzyl)-5-(trifluoromethyl)pyridin-2-amine 3q

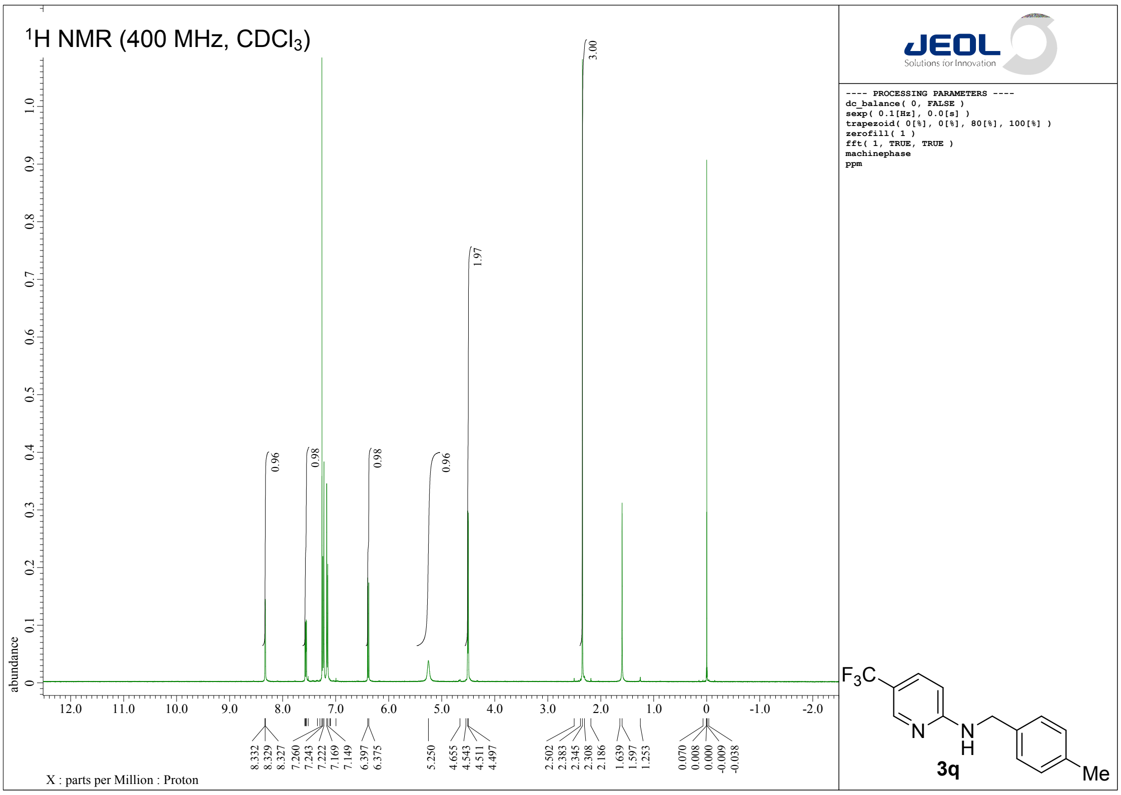

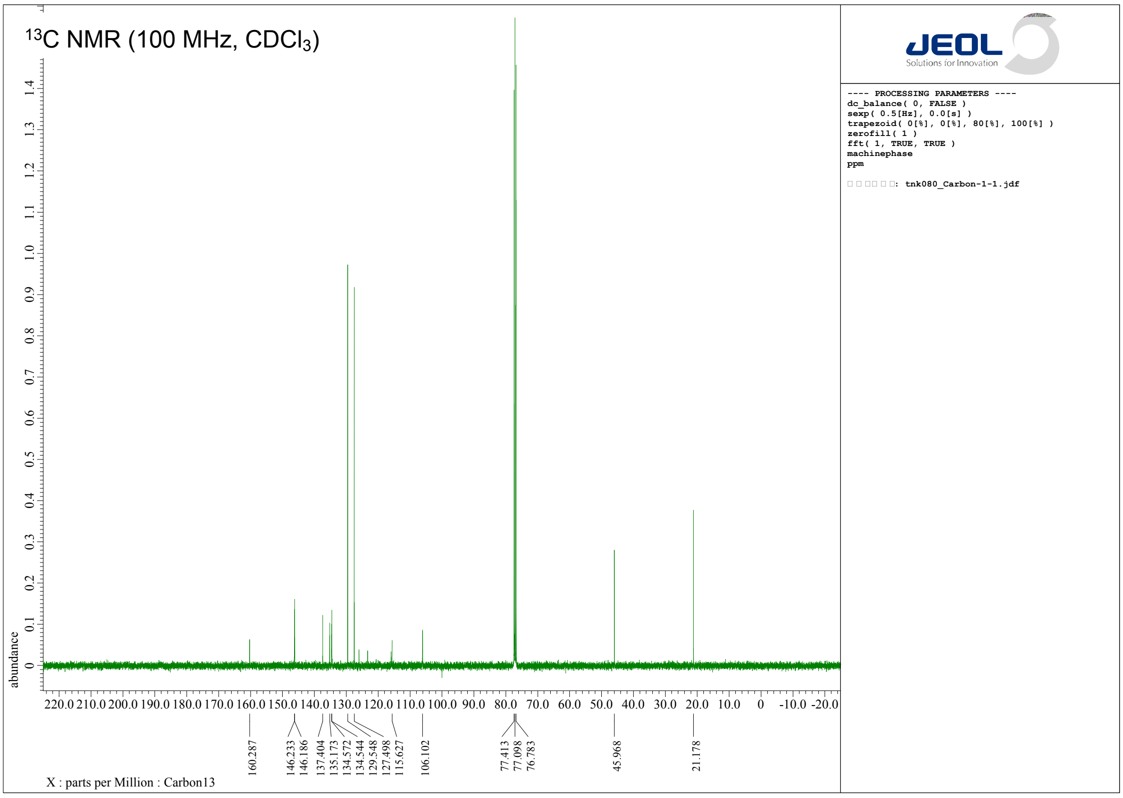

N-(3-Methylbenzyl)-5-(trifluoromethyl)pyridin-2-amine 3r

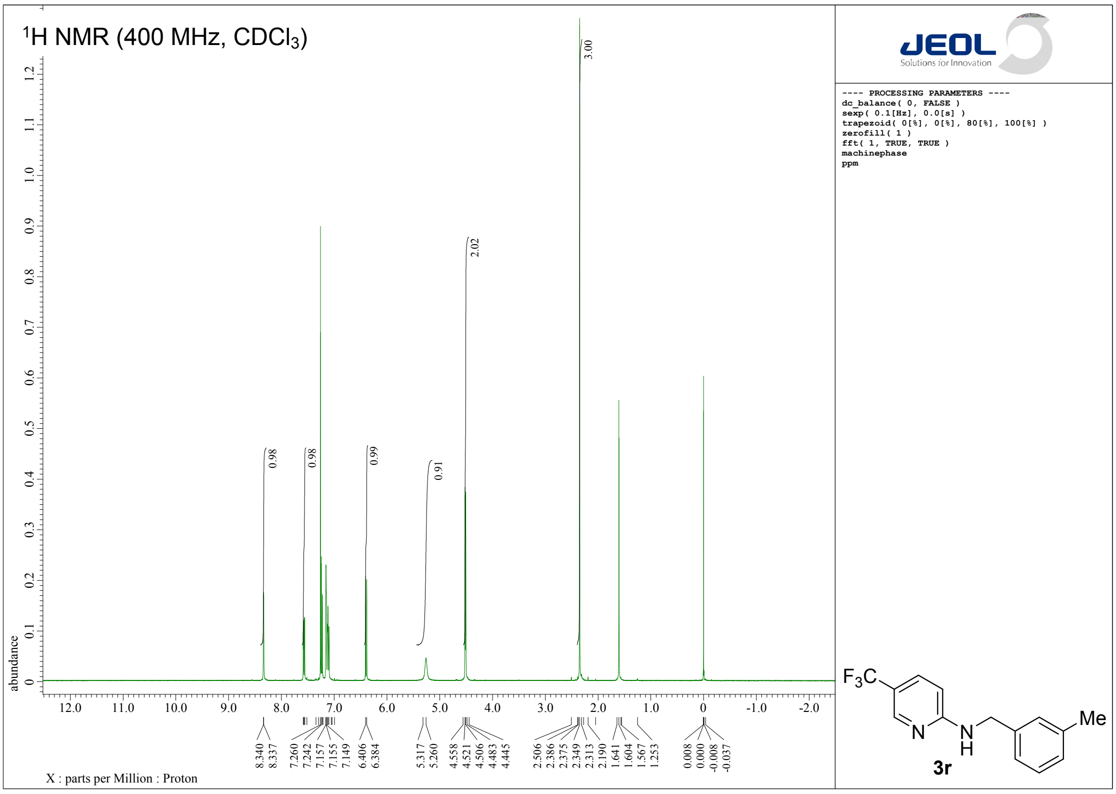

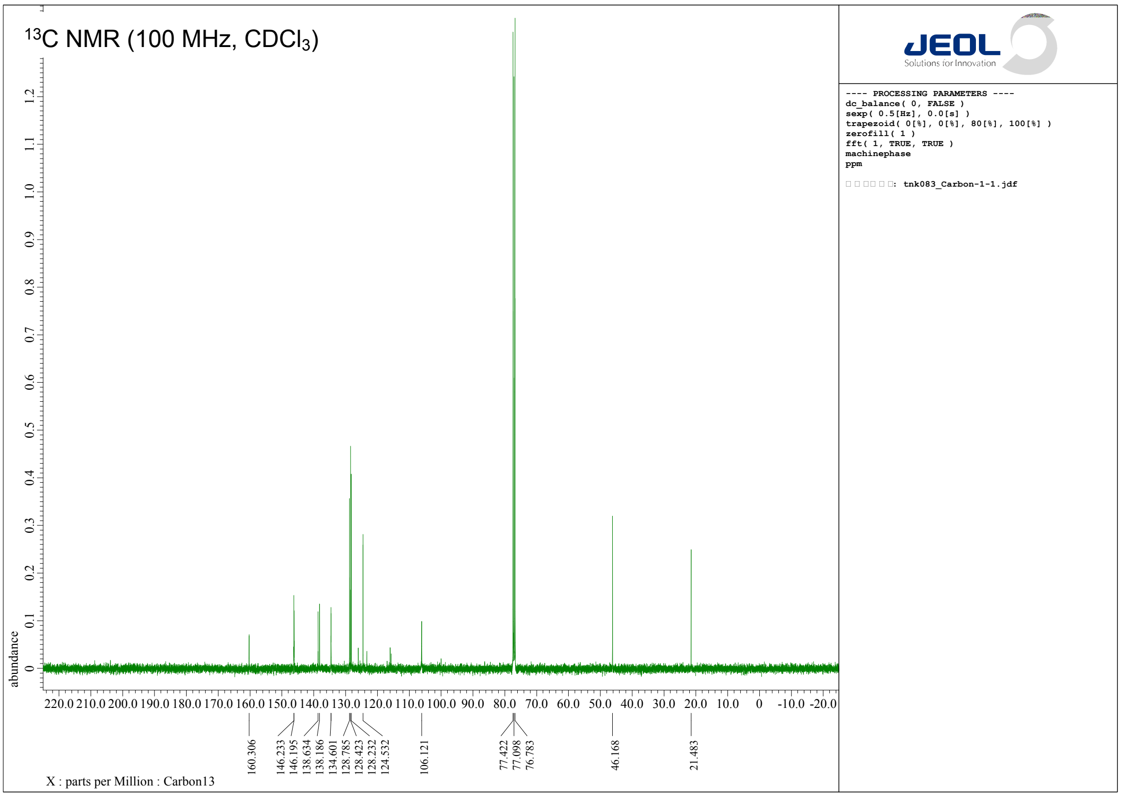

N-(2-Methoxybenzyl)-5-(trifluoromethyl)pyridin-2-amine 3s

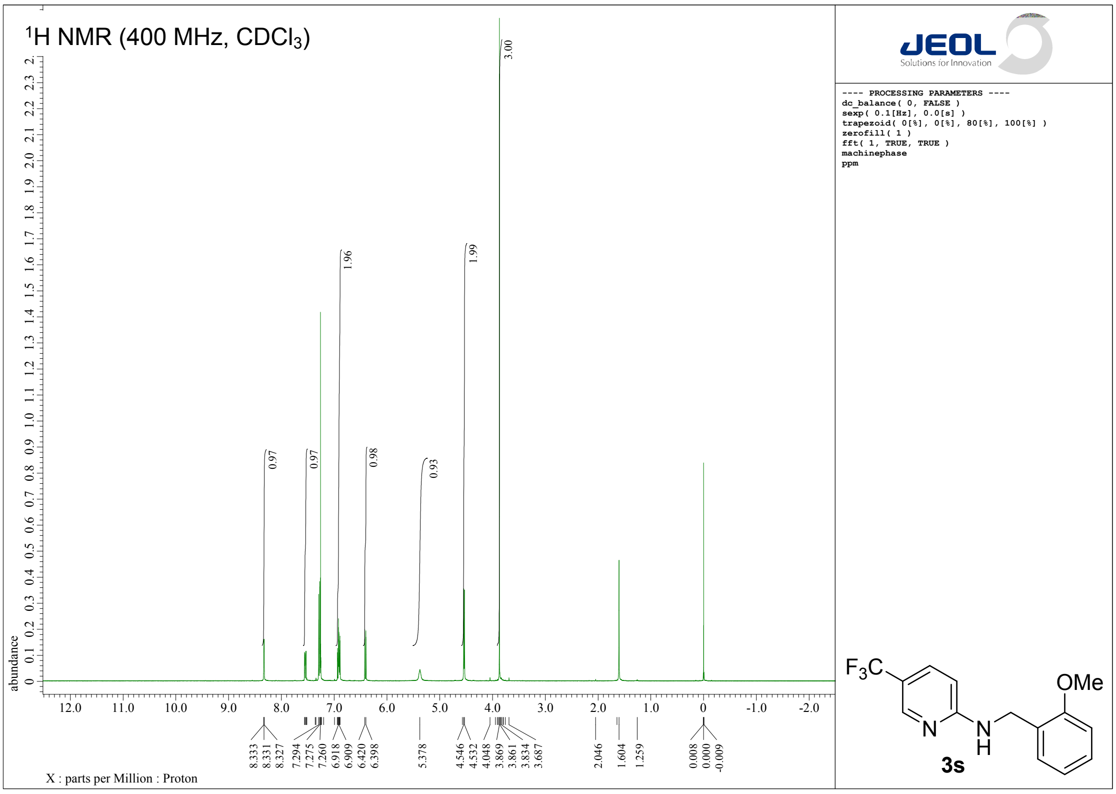

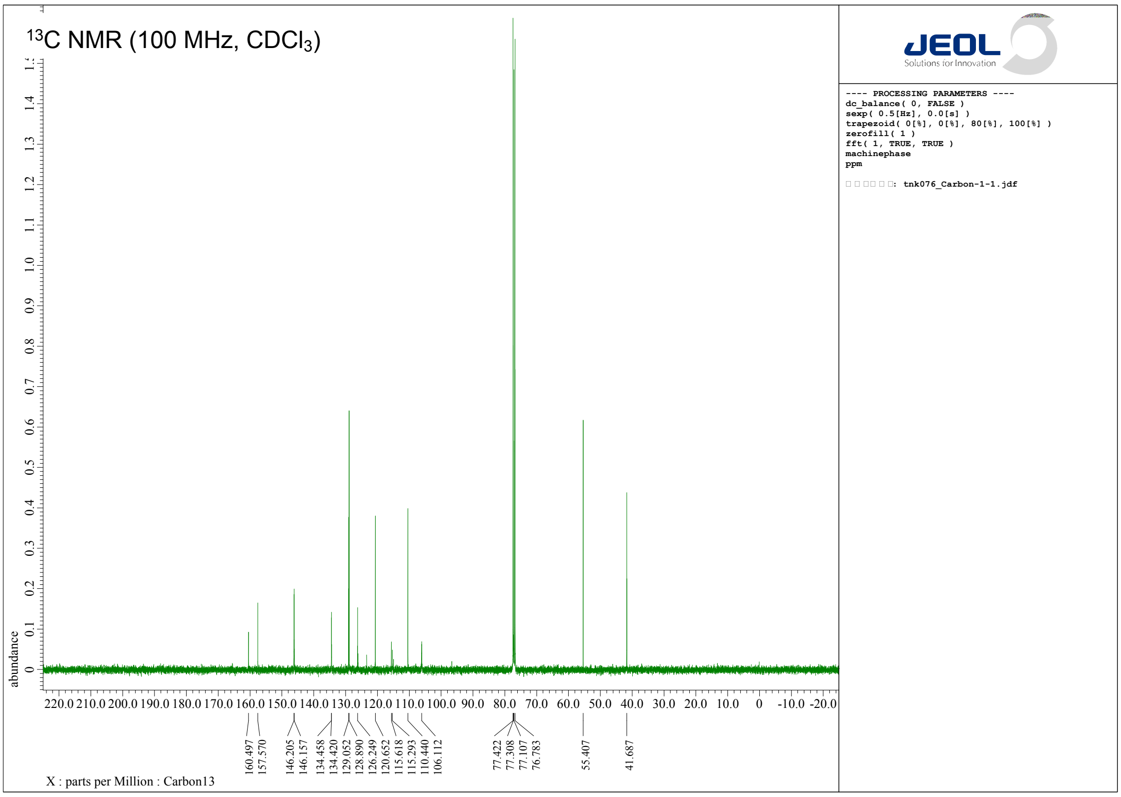

N-(4-Fluorobenzyl)-5-(trifluoromethyl)pyridin-2-amine 3t

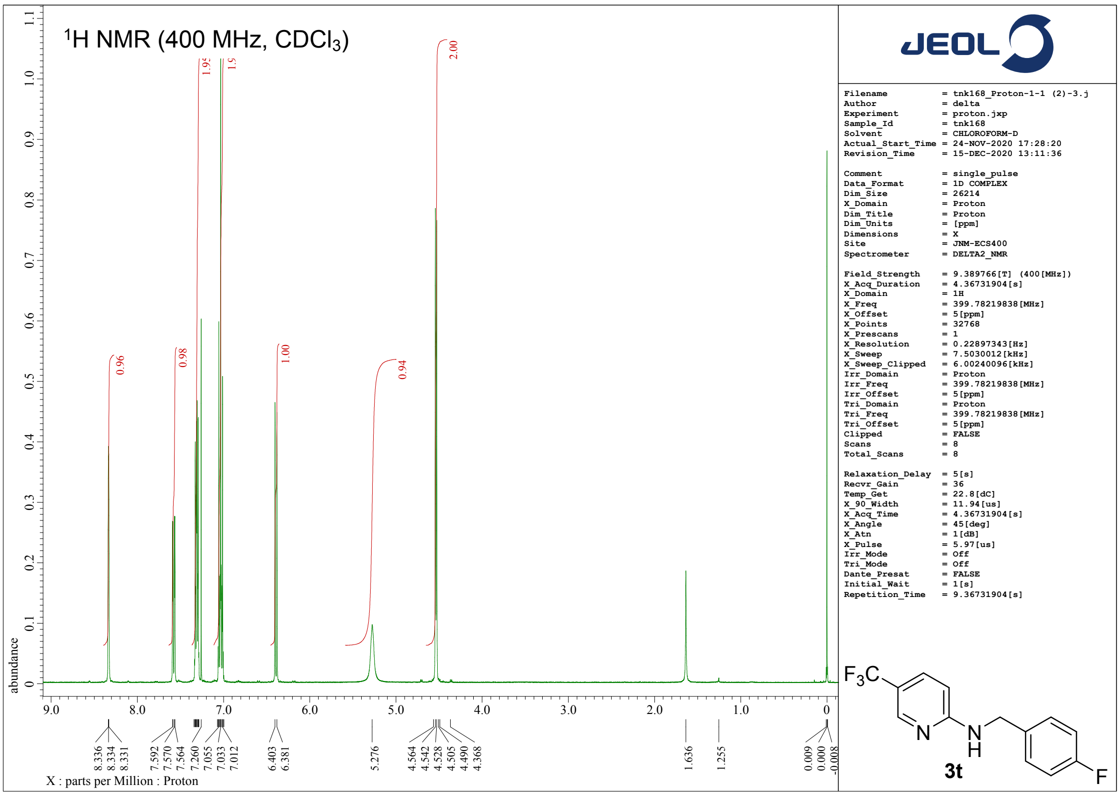

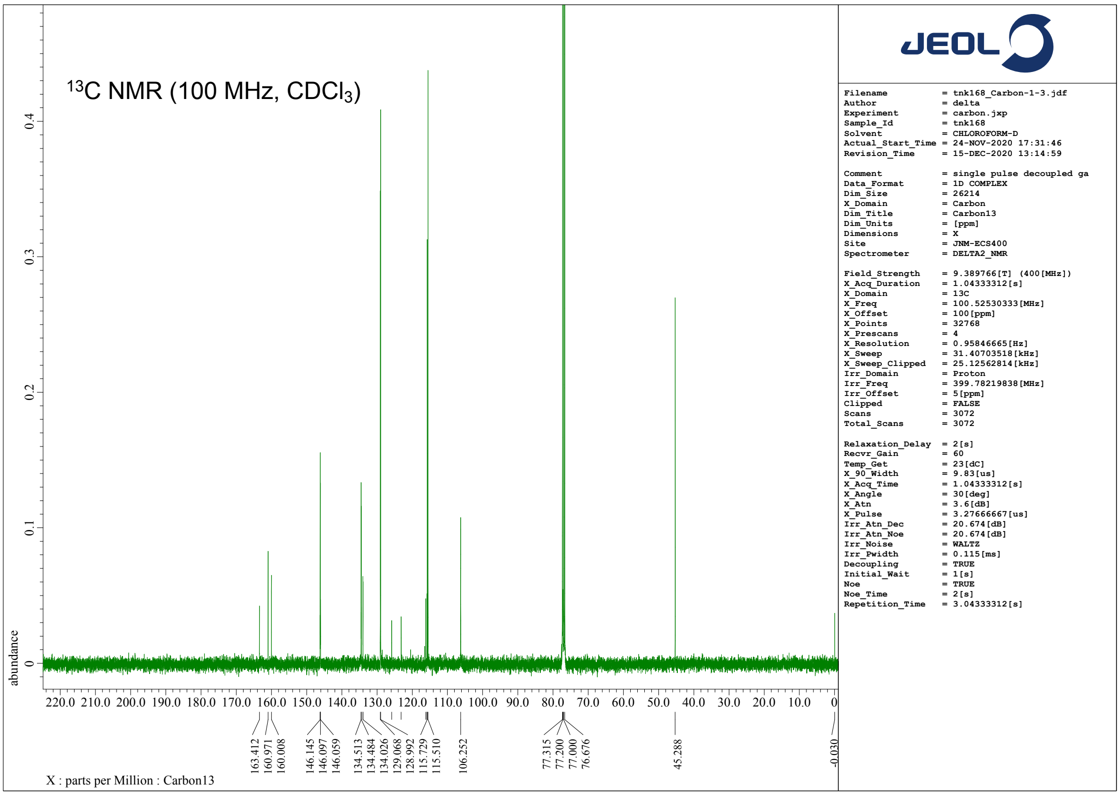

N-(3-Fluorobenzyl)-5-(trifluoromethyl)pyridin-2-amine 3u

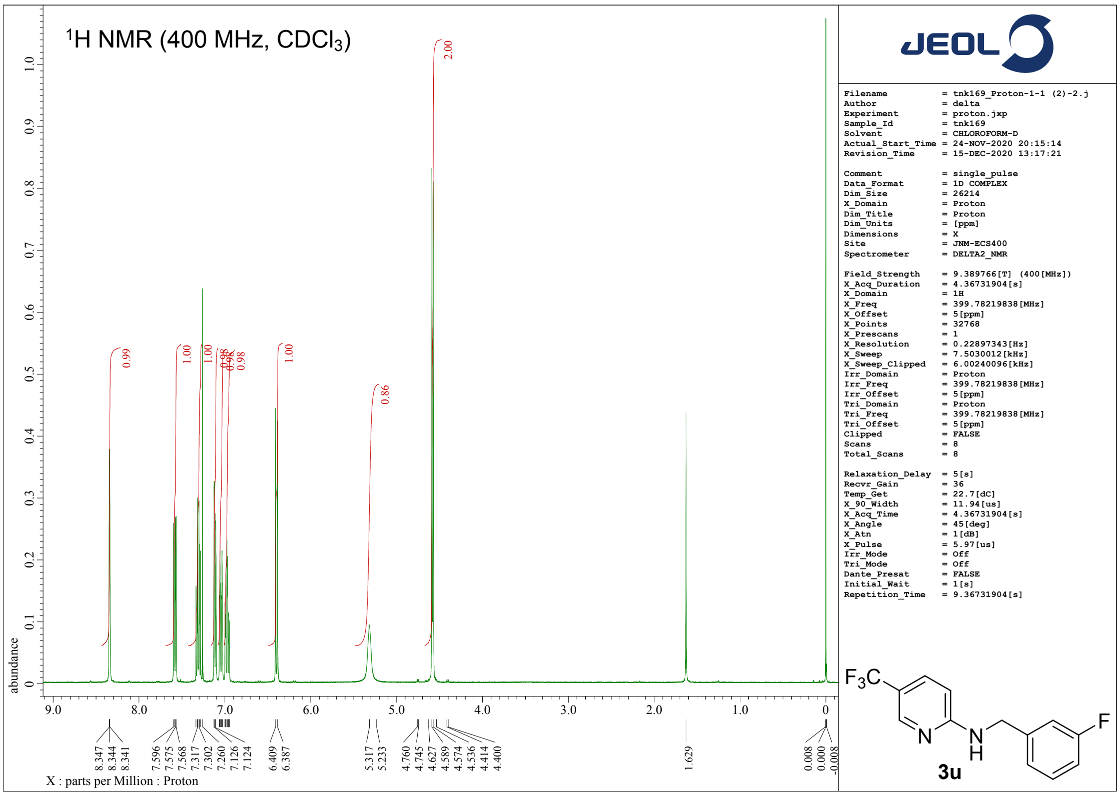

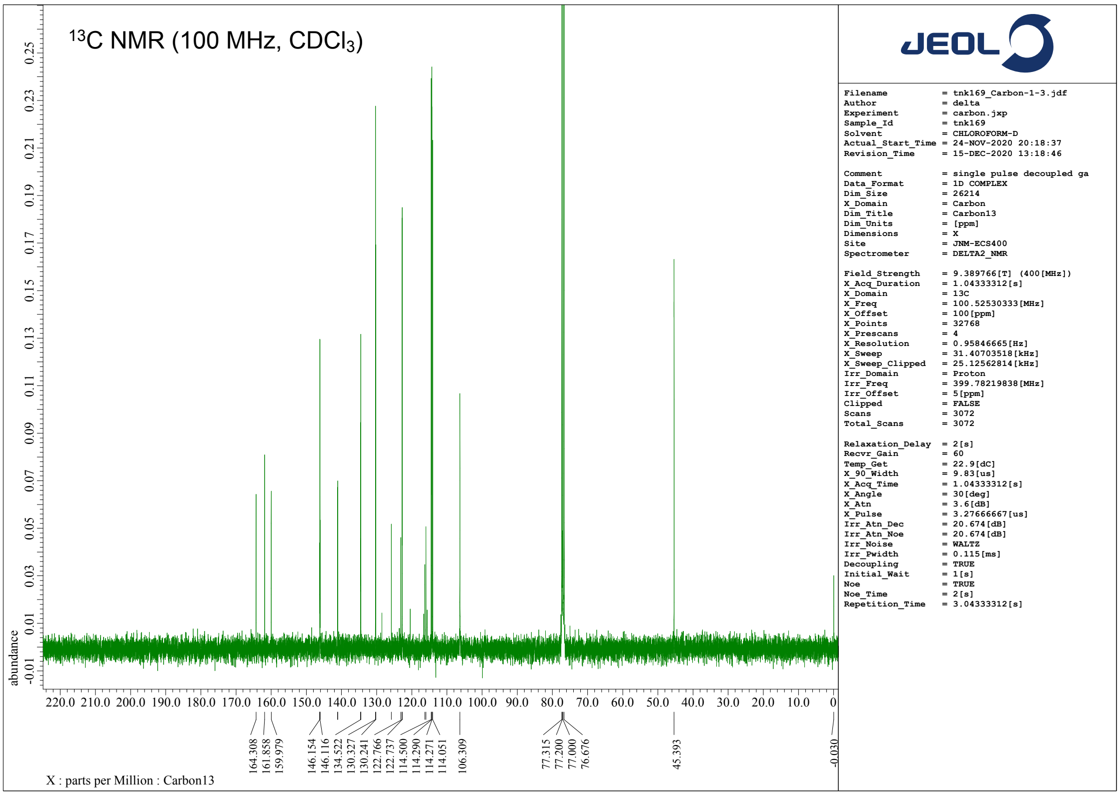

5-Fluoro-N-(4-fluorobenzyl)pyridin-2-amine 3v

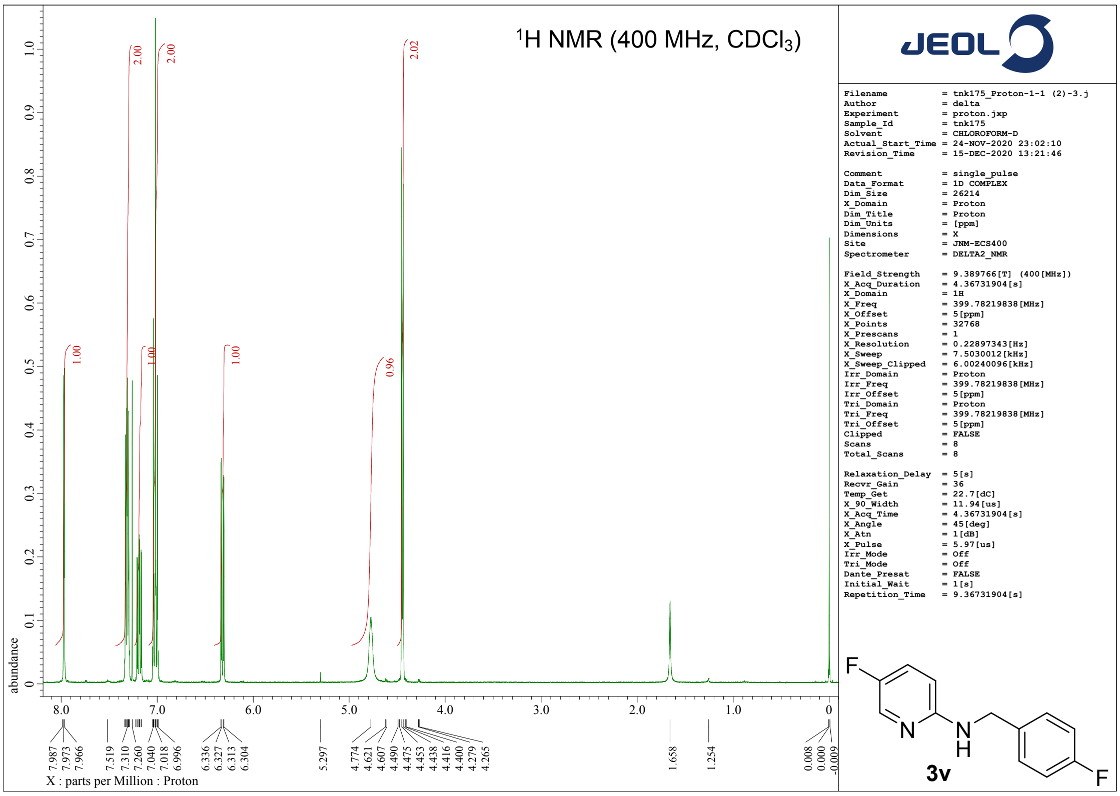

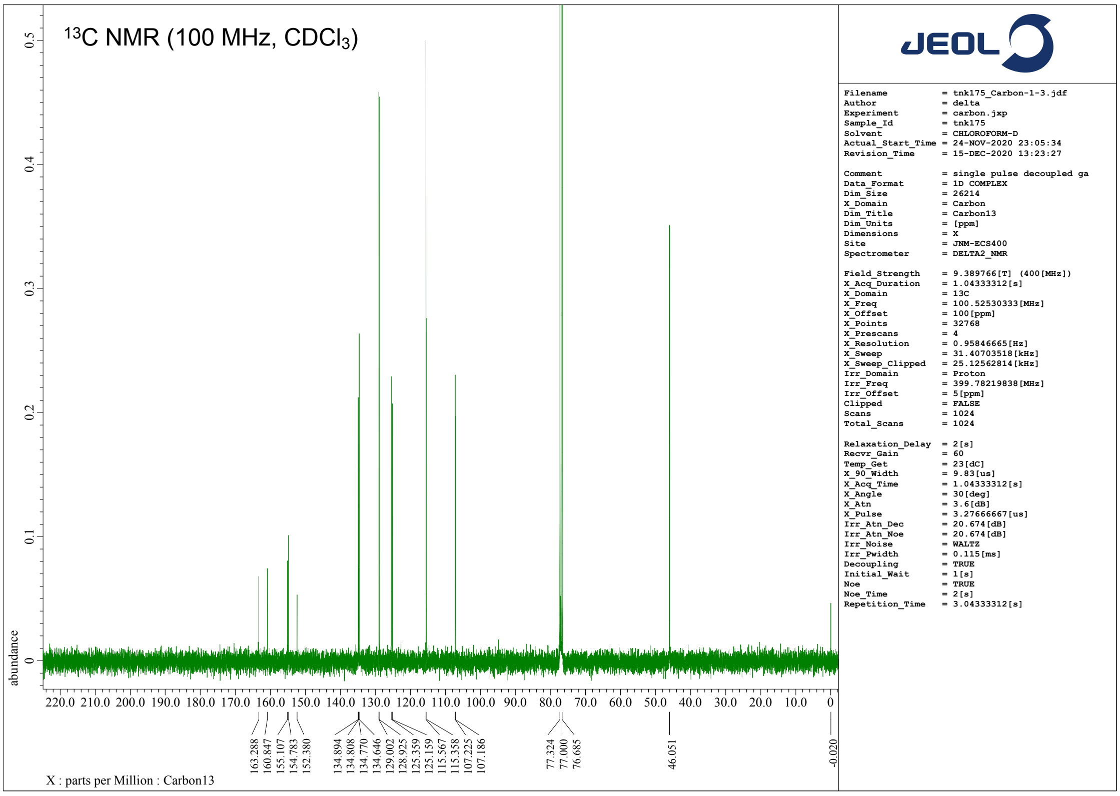

Supplement: RA-011-D1RA04118E-s001 [file RA-011-D1RA04118E-s001.pdf]
